# DNA methylation affects photoperiodic tuberization in potato (*Solanum tuberosum* L.) by mediating the expression of genes related to the photoperiod and GA pathways

**DOI:** 10.1038/s41438-021-00619-7

**Published:** 2021-09-01

**Authors:** Yanjun Ai, Shenglin Jing, Zhengnan Cheng, Botao Song, Conghua Xie, Jun Liu, Jun Zhou

**Affiliations:** 1 Key Laboratory of Potato Biology and Biotechnology, Ministry of Agriculture and Rural Affairs, Huazhong Agricultural University, Wuhan, Hubei 430070 China; 2 Key Laboratory of Horticultural Plant Biology (HZAU), Ministry of Education, Wuhan, Hubei 430070 China; 3 College of Life Sciences and Technology, Huazhong Agricultural University, Wuhan, Hubei 430070 China; 4 College of Horticulture and Forestry Sciences, Huazhong Agricultural University, Wuhan, Hubei 430070 China; 5 Hubei Vocational College of Bio-Technology, Wuhan, Hubei 430070 China

**Keywords:** Epigenomics, Light responses

## Abstract

Overcoming short-day-dependent tuberization to adapt to long-day conditions is critical for the widespread geographical success of potato. The genetic pathways of photoperiodic tuberization are similar to those of photoperiodic flowering. DNA methylation plays an important role in photoperiodic flowering. However, little is known about how DNA methylation affects photoperiodic tuberization in potato. Here, we verified the effect of a DNA methylation inhibitor on photoperiodic tuberization and compared the DNA methylation levels and differentially methylated genes (DMGs) in the photoperiodic tuberization process between photoperiod-sensitive and photoperiod-insensitive genotypes, aiming to dissect the role of DNA methylation in the photoperiodic tuberization of potato. We found that a DNA methylation inhibitor could promote tuber initiation in strict short-day genotypes. Whole-genome DNA methylation sequencing showed that the photoperiod-sensitive and photoperiod-insensitive genotypes had distinct DNA methylation modes in which few differentially methylated genes were shared. Transcriptome analysis confirmed that the DNA methylation inhibitor regulated the expression of the key genes involved in the photoperiod and GA pathways to promote tuber initiation in the photoperiod-sensitive genotype. Comparison of the DNA methylation levels and transcriptome levels identified 52 candidate genes regulated by DNA methylation that were predicted to be involved in photoperiodic tuberization. Our findings provide a new perspective for understanding the relationship between photoperiod-dependent and GA-regulated tuberization. Uncovering the epigenomic signatures of these pathways will greatly enhance potato breeding for adaptation to a wide range of environments.

## Introduction

Potato (*Solanum tuberosum* L.) originated in the South American Andes and has been the main food consumed by the local population for over 8000 years^1^. During the 16th and 17th centuries, potato spread to Europe and then around the world^2^. Today, potato is widely grown in more than 150 countries and regions worldwide and has become the third largest food crop consumed after rice and wheat (http://faostar.fao.org). Adaptation to different environments, especially to long-day conditions, plays a critical role in the widespread geographical success of this crop^3^. The tuber formation process (tuberization) is essential for helping potato survive in cold winters and is also the determinant step for economic yield^4^. Therefore, overcoming the short-day dependency for tuberization in Andean potatoes has been considered the most important adaptation to new environments. Modern potato cultivars evolved to tuberize under both short-day and long-day conditions after long-term domestication, but tuber differentiation was still accelerated by shorter day lengths in all potato genotypes.

To date, much progress in understanding the molecular mechanism underlying day-length tuberization control in potato has been made, and several key elements are shared by photoperiodic control of tuberization in potato and photoperiodic flowering in Arabidopsis^5,6^. Briefly, the day length is perceived by the photoreceptor in leaves, and then the signal is transported to underground stolons to induce tuber initiation^5^. In potato, phytochromes are the main photoreceptors for day-length sensing, and phytochromes B and F play key roles in photoperiodic tuberization control by regulating the CO-FT pathway^7,8^. *StSP6A*, an FT homolog, has been identified to be a tuberigen that can be transported from leaves to stolons to induce tuber initiation^9^. Another FT homolog, *StSP5G*, acts as a repressor of tuberization by repressing the expression of *StSP6A* under long-day (LD) conditions^7^. The CO homolog *StCOL1* activates the expression of *StSP5G* under LD conditions and then inhibits the expression of *StSP6A* indirectly^7,10^. *CYCLING DOF FACTOR1* (*StCDF1*) is another key gene associated with the CO-FT tuberization pathway; it promotes tuberization by repressing the expression of *StCOL1* and unblocking the *StSP6A* pathway^10^. In addition, the BEL1 transcription factors *StBEL5*^11^ and *StBEL11/29*^12^ and two microRNAs, *miR172*^13^ and *miR156*^14^, are also involved in tuber formation regulation as long-distance signals. However, further work is still needed to completely understand photoperiodic tuberization in potato.

Gene function is affected not only by genetic variation but also by epigenetic modification. DNA methylation, one of the most important epigenetic modifications, plays essential roles in several important plant development processes, such as flowering^15,16^, fruit ripening^17,18^, and seed germination^19^. It has been reported in different species that DNA methylation could change the photoperiod sensitivity of flowering by regulating the expression of photoperiodic flowering-related genes^15,20^. Furthermore, DNA methylation is also responsible for domestication traits such as flowering time and adaptation to different environments in allotetraploid cotton^21^. In potato, research on epigenetic modifications in tuberization is limited. Recently, Kumar et al. found that *miR156* overexpression reduces belowground tuber yield but stimulates aerial tubers in potato (*Solanum tuberosum* ssp *andigena*) under short-day conditions, and two histone modifiers (*StMSI1* and *StBMI1-1*) regulate miR156 and alter the hormonal response during this process^22^.

Previously, we identified a group of candidate genes potentially associated with photoperiodic tuberization^23^ and found that many of these genes were differentially expressed in photoperiodic-sensitive and photoperiodic-insensitive genotypes; however, we did not find any DNA variation. Considering the influence of DNA methylation on photoperiodic flowering, we hypothesize that DNA methylation might be involved in the regulation of the expression of these genes and affect photoperiodic tuberization in potato. In the present study, we verified the effects of DNA methylation inhibitors on photoperiodic tuberization and compared whole-genome DNA methylation between photoperiod-sensitive and photoperiod-insensitive genotypes. The relationship between DNA methylation and transcripts was clarified, aiming to identify the key candidate photoperiodic tuberization genes regulated by DNA methylation. Our results will shed light on the mechanism by which potato photoperiodic tuberization is epigenetically regulated and provide a new understanding of long-day adaptation in potato.

## Materials and methods

### Plant materials

Three potato clones, E26, E108, and E20, which are progenies of the tetraploid potato cross 393075.54 × 391679.12 (seeds were offered by the International Potato Center), were used in the present research. This population has been characterized for in vitro tuberization phenotypes under short-day (SD, 8 h day/16 h night) and long-day (LD, 16 h day/8 h night) conditions. E26 tuberizes only under SD conditions, E108 forms tubers in both SD and LD conditions, and E20 does not initiate tubers in either condition^24^.

For phenotyping after DNA methylation inhibitor treatment, 40 μM zebularine (Zeb)^25^ was used in the induction medium, and water instead of Zeb was used as a control. In vitro tuberization under SD and LD conditions was performed as described. All plantlets (including stems, leaves, and stolons but not tubers) of the three genotypes were sampled at 21, 28, 35, 42, 49, and 56 days, immediately frozen in liquid nitrogen, and kept at −80 °C until use for RNA extraction and qRT-PCR analysis.

For DNA methylation sequencing and RNA sequencing, single stem nodes (not including the apical meristem) of 4-week-old plantlets, including leaves, were cultured for 3 weeks on tuberization induction medium (MS supplemented with 8% sucrose, 0.7% agar, and 0.2% activated carbon) under LD conditions and then moved to SD conditions for tuberization induction^23^. All plantlets (including stems, leaves, and stolons but not tubers) were sampled at 0, 7, 14, 21, and 28 days under SD conditions, immediately frozen in liquid nitrogen, and kept at −80 °C until used for DNA/RNA extraction. For DNA methylation sequencing, DNA at day 0 was taken as a control and named E26CK, E20CK, and E108CK for each genotype. DNA samples of each genotype at 7, 14, and 21 days were mixed in equal proportions and named E26SD, E20SD, and E108SD. Samples treated with zebularine and the control of E26 were collected at 0, 7, 21, and 28 days for RNA sequencing and named Zeb_0d, Zeb_7d, Zeb_21d, and Zeb_28d; CK_0d, CK_7d, CK_21d, and CK_28d.

### MethylRAD sequencing and DNA methylation data analysis

Genomic DNA was extracted from each of the three genotypes at each timepoint by the cetyltrimethylammonium bromide (CTAB) method^26^; then, MethylRAD library preparation and sequencing were conducted according to the protocol described by Wang et al.^27^. PE sequencing was performed on the Illumina HiSeq X-Ten platform. DNA samples at 7, 14, and 21 days under SD conditions were mixed in equal proportions to compose a DNA pool of each genotype for MethylRAD library construction. In total, six libraries (E26CK, E20CK, E108CK, E26SD, E20SD, and E108SD) were constructed and then sequenced with three technological replicates.

After QC and filtering of the original reads and removal of the sequences with linkers, low-quality sequences (more than 5 bases with a quality lower than 10), and those with Ns (unidentified bases), the high-quality clean reads containing the methylated CCGG/CCWGG sites were mapped to the reference sequence (signatures with CCGG/CCWGG sites) of the *Solanum tuberosum* genome PGSC v.4.04 (http://solanaceae.plantbiology.msu.edu/data/potato_dm_v404_all_pm_un.fasta.zip) by the SOAP program (version 2.21, parameter: -M 4 -v 2 -r 0). Sites covered by at least three reads were regarded as reliable DNA methylation sites. Then, the number of methylated sites and the depth of signature coverage of each sample were calculated. The methylation levels of a site (CCGG/CCWGG) could be reflected by the sequencing depth of the methylated signature. The unit of the quantitative value of site methylation was RPM (reads per million), which means that the quantitative value of the methylation level of a site was equal to the coverage at that site in number of reads/the number of high-quality reads in the library multiplied by 1,000,000. Furthermore, the distributions of the methylated CCGG/CCWGG sites on different elements of the genome, especially on the different regions of genes, were evaluated by SnpEff software (version: 4.1g)^28^ and bed tools software (v2.25.0)^29^. Then, the DNA methylation levels of the genes were evaluated by summing the methylation levels of sites that were localized in the gene region^30^. The differential DNA methylation levels of the sites and genes were identified by using the R package DESeq^31^, with the thresholds |log_2_(fold-change)| >1 and corrected *p*-value ≤0.05. Finally, the genes with different methylation levels in different samples were further analyzed based on Gene Ontology (GO) enrichment by AgriGO (http://bioinfo.cau.edu.cn/agriGO/analysis.php) and Kyoto Encyclopedia of Genes and Genomes (KEGG, http://www.genome.jp/kegg/) enrichment using the hypergeometric distribution test with the threshold FDR ≤ 0.05. Methylome data for the DNA methylation of CCGG and CCWGG sites detected in the SD and CK samples of E26, E20, and E108 (6 samples) have been uploaded as an attachment (Data sets 1 and 2).

### RNA sequencing and data analysis

The gene expression profiles of the zebularine treatment group (Zeb_0d, Zeb_7d, Zeb_21d, and Zeb_28d) and control group (CK_0d, CK_7d, CK_21d, and CK_28d) of E26 were determined by UMI-RNA-seq (SeqHealth Tech, China) as described by Kivioja et al.^32^. Briefly, 2 μg of total RNA was used for stranded RNA sequencing library preparation using the KC-Digital^TM^ Stranded mRNA Library Prep Kit for Illumina^®^ (Catalog NO. DR08502, Wuhan SeqHealth Co., Ltd., China) by following the manufacturer’s instruction. The kit eliminates duplication bias in PCR and sequencing steps by using unique molecular identifiers (UMIs) of 8 random bases to label the preamplified cDNA molecules. The library products corresponding to 200–500 bp were enriched, quantified, and finally sequenced on an Illumina HiSeq X-Ten sequencer.

After raw data cleaning (filtering out the adaptor and low-quality reads) by Trimmomatic (version 0.36)^33^ and UMI deduplication of clean reads (in-house software)^34^, the deduplicated consensus sequences were used for standard RNA-seq analysis. These sequences were mapped to the *Solanum tuberosum* genome PGSC v.4.04 (http://solanaceae.plantbiology.msu.edu/data/potato_dm_v404_all_pm_un.fasta.zip) using STAR (version 2.5.3a) with default parameters^35^. The mapped reads were analyzed by featureCounts^36^, and then RPKMs (reads per kilobase of transcript per million mapped reads) were calculated. Differential expression was estimated with the edgeR package^37^, and the DEGs (differentially expressed genes) were identified with the threshold FDR ≤ 0.05 and |log_2_(fold-change)| ≥2. Pathway analysis of DEGs was conducted with Gene Ontology (GO) enrichment by AgriGO (http://bioinfo.cau.edu.cn/agriGO/analysis.php) and Kyoto Encyclopedia of Genes and Genomes (KEGG, http://www.genome.jp/kegg/) enrichment using the hypergeometric distribution test with the threshold FDR ≤ 0.05. The complete raw RNA-seq data have been uploaded to NCBI (PRJNA700857).

### RNA extraction and qRT-PCR analysis

RNA isolation from the frozen samples was performed using a Total RNApure Kit (ZOMANBIO, http://zomanbio.com). Reverse transcription into cDNA was carried out using 5× All-in-One RT MasterMix (ABM, http://www.abmgood.com). Quantitative RT-PCR was performed with the Bio-Rad CFX Connect^TM^ Real-Time System (Bio-Rad, http://www.bio-rad.com) and EvaGreen 2X qPCR MasterMix (ABM, http://www.abmgood.com). The potato *ef1α* gene (GenBank accession: AB061263) was used as a control gene for normalization of expression^38^. Gene expression levels were calculated via the 2^−Δcq^ method described by Bio-Rad (http://www.bio-rad.com/zh-cn/applications-technologies/real-time-pcr-experimental-design). All primer sequences for qRT-PCR analysis are detailed in Supplementary Table S1.

## Results

### A DNA methylation inhibitor promoted in vitro tuberization in a photoperiod-sensitive genotype

**Fig. 1:**
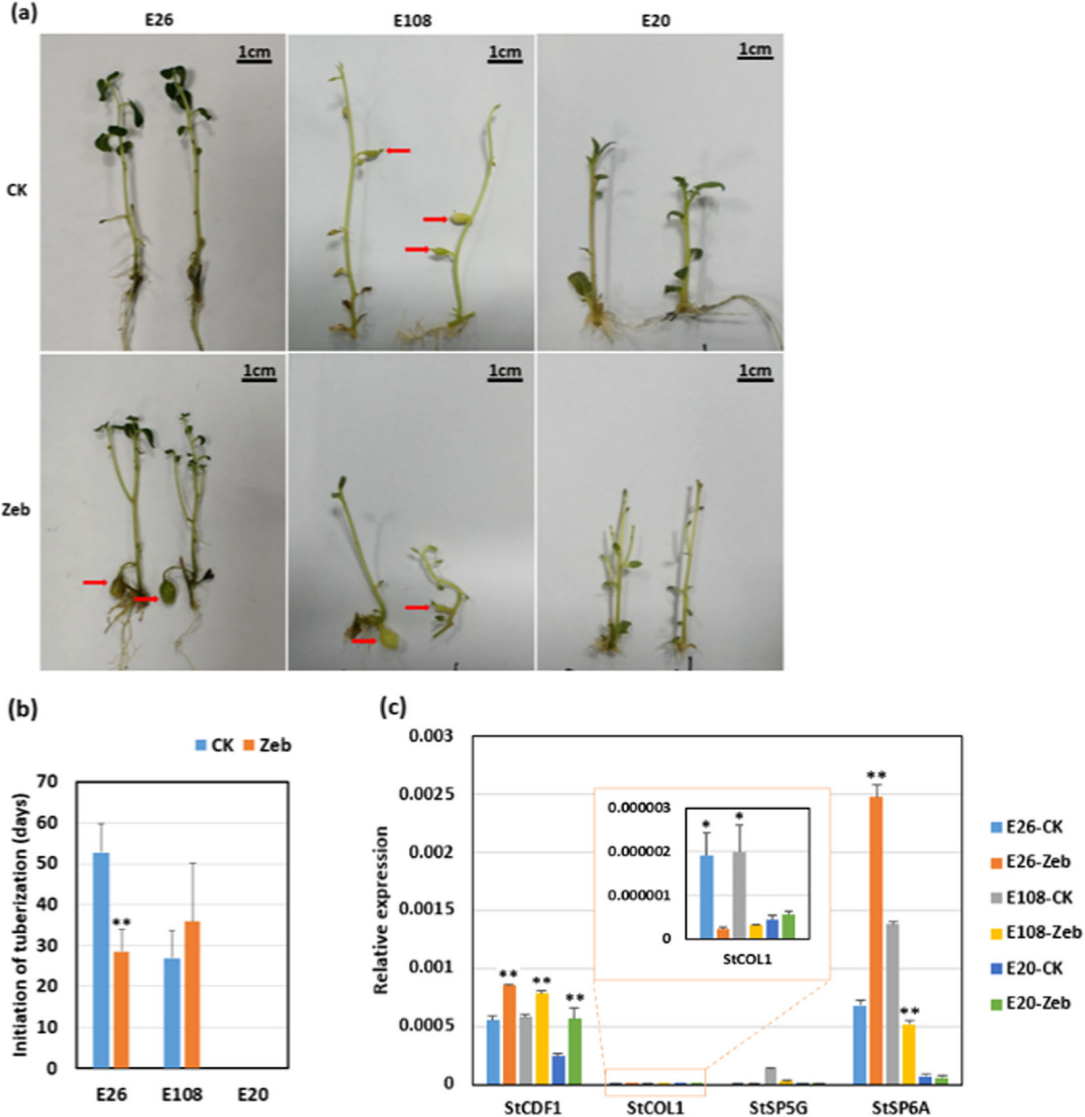
Application of a DNA methylation inhibitor promoted the initiation of in vitro tuber formation in the photoperiod-sensitive genotype. **a** Tuberization phenotypes of E26, E108, and E20 grown under SD conditions (8-h light/16-h dark) for 28 days and treated with the DNA methylation inhibitor (Zeb) or water (CK). Tuberization of the photoperiod-sensitive genotype E26 usually occurred after 40 days of growth under SD conditions, while Zeb-treated plantlets formed tubers before 28 days, and no tubers formed in the control. However, there were no effects of Zeb on the tuberization of the photoperiod-insensitive genotypes E108 and E20. Red arrows indicate microtubers. **b** Average tuberization time in the treatment (Zeb) and control (CK) groups of E26 and E108 under SD conditions. **c** Expression of the genes involved in the CO-FT pathway in the treatment (Zeb) and control (CK) groups grown under SD conditions for 28 days. The *ef1*α gene was used as an endogenous control. **P ≤ 0.01. Error bars represent the standard error of three biological replicates

To test whether changes in genome methylation might participate in the regulation of tuberization, we used a DNA methyltransferase inhibitor (zebularine) to treat the plantlets and evaluated their tuberization performance under inductive (SD) and noninductive (LD) conditions. In the photoperiod-sensitive genotype E26 (tuberized under strict SD conditions), tuber formation was observed in the treatment group several weeks before the control group under SD conditions (Fig. 1a, b), but under LD conditions, there was no tuber formation in either the treatment or control. In the other two photoperiod-insensitive genotypes, E108 (tuberized well under both SD and LD conditions) and E20 (could not tuberize under either photoperiod), there were no significant changes between the treatment and control under either short-day (Fig. 1a, b) or long-day conditions.

Furthermore, the expression of the genes involved in the CO-FT pathway in the treatment and control groups for the above three genotypes grown under SD conditions for 28 days confirmed that the tuberization marker gene StSP6A was significantly upregulated in the E26 treatment group compared to the control, but it was downregulated in E108 and nearly not expressed in E20 (Fig. 1c). These results indicated that DNA methylation inhibitors promoted tuber initiation in strict short-day genotypes by regulating the photoperiodic tuberization pathway.

### The photoperiod-sensitive genotype showed a higher DNA methylation ratio than the photoperiod-insensitive genotypes

To further investigate the role of DNA methylation in the photoperiodic tuberization of potato, we used MethylRAD sequencing to analyze the DNA methylation at CCGG and CCWGG (W = T or A) sites in the whole genomes of the short-day induced (SD) and control (CK) groups of the three genotypes E20, E26, and E108. A total of 12.91–16.45 million high-quality reads were produced in the 6 samples, 24.10–36.04% of which were mapped to unique positions in the reference genome (Supplementary Table S2). In E20, 108,153 CCGG and 91,432 CCWGG methylation sites were found in the SD group, with average methylation coverage of 24.48 and 26.51, respectively. In E26, 108,760 CCGG and 96,230 CCWGG methylation sites were found in the SD group, with average methylation coverages of 21.29 and 24.48, respectively. In E108, 86,651 CCGG and 77,907 CCWGG methylation sites were found in the SD group, with average methylation coverages of 16.45 and 18.58, respectively (Supplementary Table S3).

**Fig. 2:**
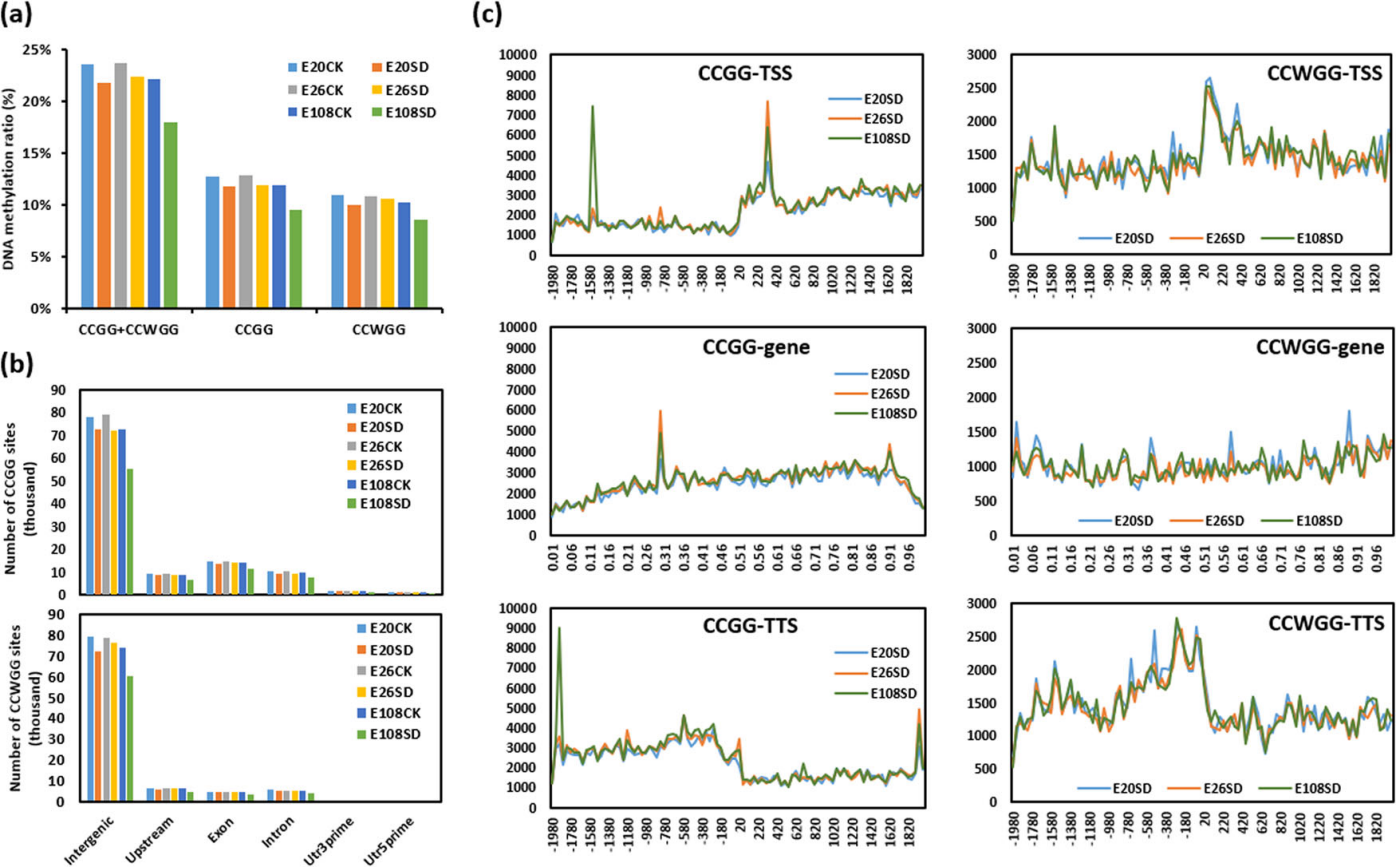
Overview of the DNA methylation ratio and levels and the distribution of CCGG and CCWGG sites in the three genotypes. **a** The genomic DNA methylation ratio at CCGG and CCWGG sites in the short-day induced (SD) and control (CK) groups of E20, E26 and E108. **b** The distributions of CCGG and CCWGG sites on different functional genic components. Upstream, exon, and intron indicate the regions 2000 bp upstream of the transcription start site (TSS), the whole exons of genes, and the whole introns of genes, respectively. Utr3prime and Utr5prime indicate the regions at the 3′ end and 5′ end of a mature transcript that are not translated into a protein. “Intergenic” indicates the intergenic regions. **c** DNA methylation levels at the CCGG and CCWGG sites in different regions of genes. The *X*-axis shows the ±2000 bp transcription start site (TSS), the relative position of the gene body, and the ±2000 bp transcription termination site (TTS). The *Y*-axis shows the relative DNA methylation level

The total DNA methylation ratios (methylated CCGG and CCWGG sites/total CCGG and CCWGG sites) were 21.78% and 23.52% in E20SD and E20CK, 22.37% and 23.60% in E26SD and E26CK, and 17.96% and 22.07% in E108SD and E108CK, respectively (Fig. 2a, Supplementary Table S4). These data indicated that E26 had a slightly higher total DNA methylation ratio than the other two genotypes in both the SD and CK groups, and SD conditions decreased the total DNA methylation ratio in all genotypes (Fig. 2a). Although there were more CCGG methylation sites than CCWGG sites in all 6 samples (Supplementary Table S3) and the DNA methylation ratio at the CCGG sites was higher than that of the CCWGG sites in each sample (Fig. 2a, Supplementary Table S4), the distribution patterns of CCGG and CCWGG methylation sites at the different elements of the genome were similar in all 6 samples. Both the CCGG and CCWGG methylated sites were concentrated in the intergenic regions, followed by the gene body (exon and intron) and upstream (2000 bp upstream of transcription start sites) regions (Fig. 2b, Supplementary Table S5). Among them, the changes in the DNA methylation levels of the gene regions, including the transcription start site (TSS), the gene coding region, and the transcription termination site (TTS), were larger at CCGG sites than at CCWGG sites, and the variation between genotypes was more significant than that between SD and CK for each genotype (Fig. 2c, Supplementary Fig. S1–3).

### Different potato genotypes with distinct photoperiod responses in tuberization show diverse methylation modes

**Fig. 3:**
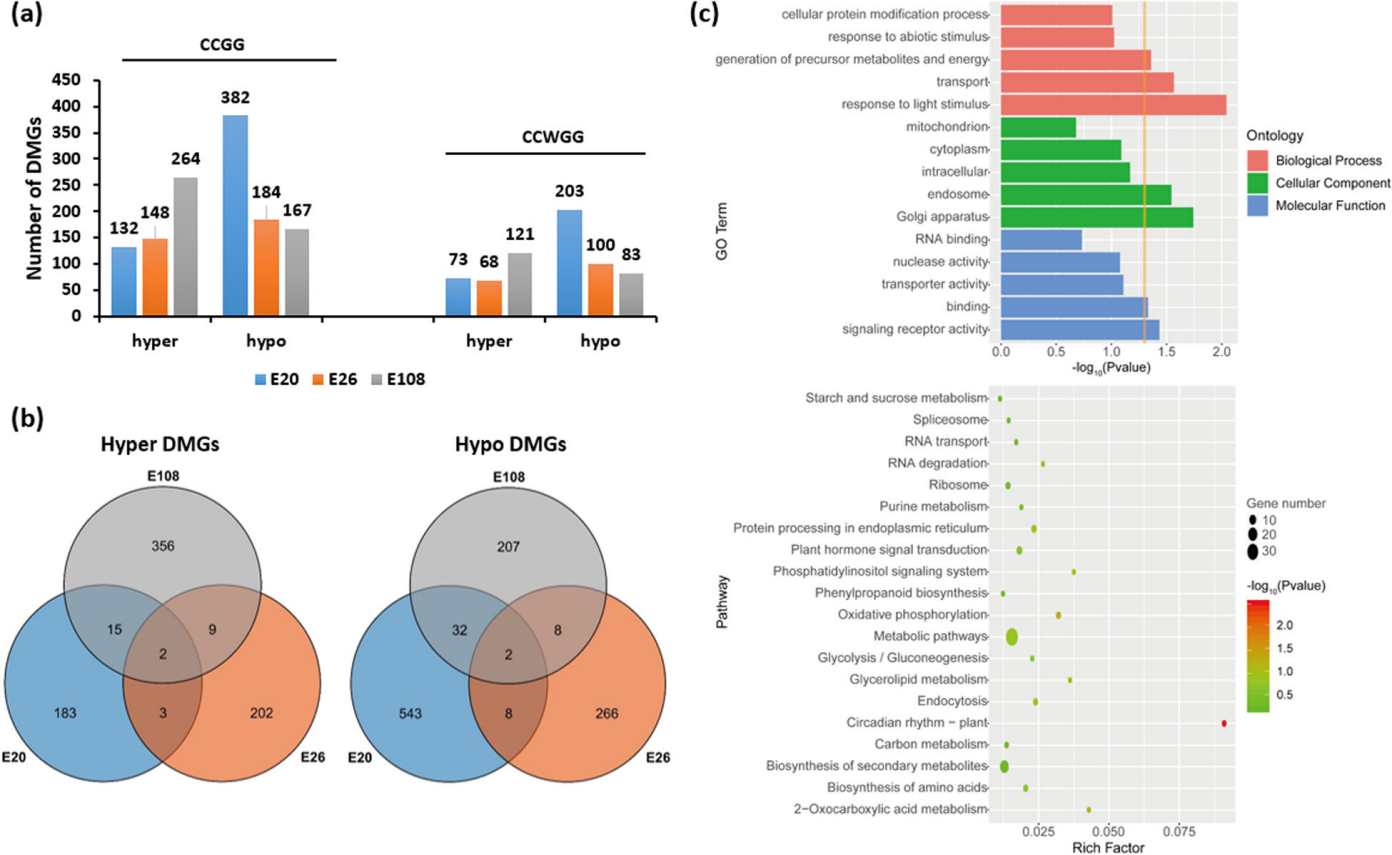
Comparative analysis of the differentially methylated genes (DMGs) in genotypes E20, E26, and E108 and GO and KEGG pathway enrichment analysis of DMGs in E26. **a** Histogram showing the numbers of DMGs in the SD group relative to the CK group of each genotype at the CCGG and CCWGG sites. **b** Venn diagrams showing overlaps among the hypermethylated (Hyper) and hypomethylated (Hypo) DMGs. **c** Gene ontology classification and KEGG pathway enrichment of DMGs in E26. For the GO classification, the *X*-axis is the −log_10_ (*P* value), and a larger value represents a higher significance of the enrichment. The *Y*-axis shows GO terms, including biological process, cellular component, and molecular function. In the KEGG pathway enrichment, the *X-*axis “rich factor” is an enrichment score; the larger the bubble is, the more genes it contains. The color of the bubbles changes from green to red, indicating an increase in the −log_10_ (*P* value), and the concentration is indicated by the −log_10_ (*P* value) and the enrichment score. The *Y*-axis shows the cell signal pathways

The DNA methylation levels of genes were evaluated by summing the methylation levels of sites localized in the gene regions. An analysis of differentially methylated genes (DMGs) was conducted for the SD and CK groups for each genotype; 785 (202 hypermethylated and 583 hypomethylated genes in the SD group), 500 (216 hypermethylated and 284 hypomethylated genes in the SD group), and 631 (382 hypermethylated and 249 hypomethylated genes in the SD group) DMGs were identified in E20, E26, and E108, respectively (Supplementary Table S6). These data indicated that in the process of SD-induced tuberization, most of the DMGs in E20 (74.3%) and E108 (60.5%) were hypomethylated, while this value was 43.2% in E26. In addition, there were more DMGs at the CCGG sites than at the CCWGG sites in all three genotypes, and the proportion of hyper- and hypomethylated DMGs in each genotype was similar between the CCGG and CCWGG sites (Fig. 3a, Supplementary Table S6). A comparison of the DMGs among genotypes with distinct photoperiod responses in tuberization showed that very few genes were shared, including photoperiodically regulated factors that are essential to tuberization (Fig. 3b, Supplementary Table S12). This suggests that these factors have different responses to the photoperiodic tuberization process in gene methylation.

To understand the potential roles of DNA methylation in tuberization, we performed Gene Ontology (GO) and KEGG enrichment analysis of all DMGs in E20, E26, and E108 (Supplementary Fig. S4–6). In E26, genes involved in the biological processes “response to light stimulus”, “transport”, and “generation of precursor metabolites and energy” and genes involved in the molecular functions “signaling receptor activity” and “binding” were enriched. Importantly, genes involved in the pathway “circadian rhythm” (*Phytochrome F, Phytochrome B2, Flowering locus T JHL23J11.9 protein*, and *Transcription factor HY5*) were significantly enriched (*P* ≤ 0.01); this pathway has been reported to be associated with photoperiodic tuberization (Fig. 3c). In E20, genes involved in “DNA metabolic process”, “cell cycle” and “metabolic process” were enriched, and 9 pathways involved in the biosynthesis and metabolism of amino acids were enriched. In E108, only one GO term, “DNA metabolic process”, was enriched, and 7 pathways were enriched, “lysine degradation”, “endocytosis”, “nucleotide excision repair”, “inositol phosphate metabolism”, “basal transcription factors”, “2-oxocarboxylic acid metabolism”, and “N-glycan biosynthesis” (Supplementary Fig. S4–6). Our analysis suggests that only the DMGs in the photoperiod-sensitive genotype (E26) were involved in the photoperiodic tuberization pathway.

### Early tuber formation was associated with photoperiodic tuberization and GA pathway-related genes regulated by DNA methylation

**Fig. 4:**
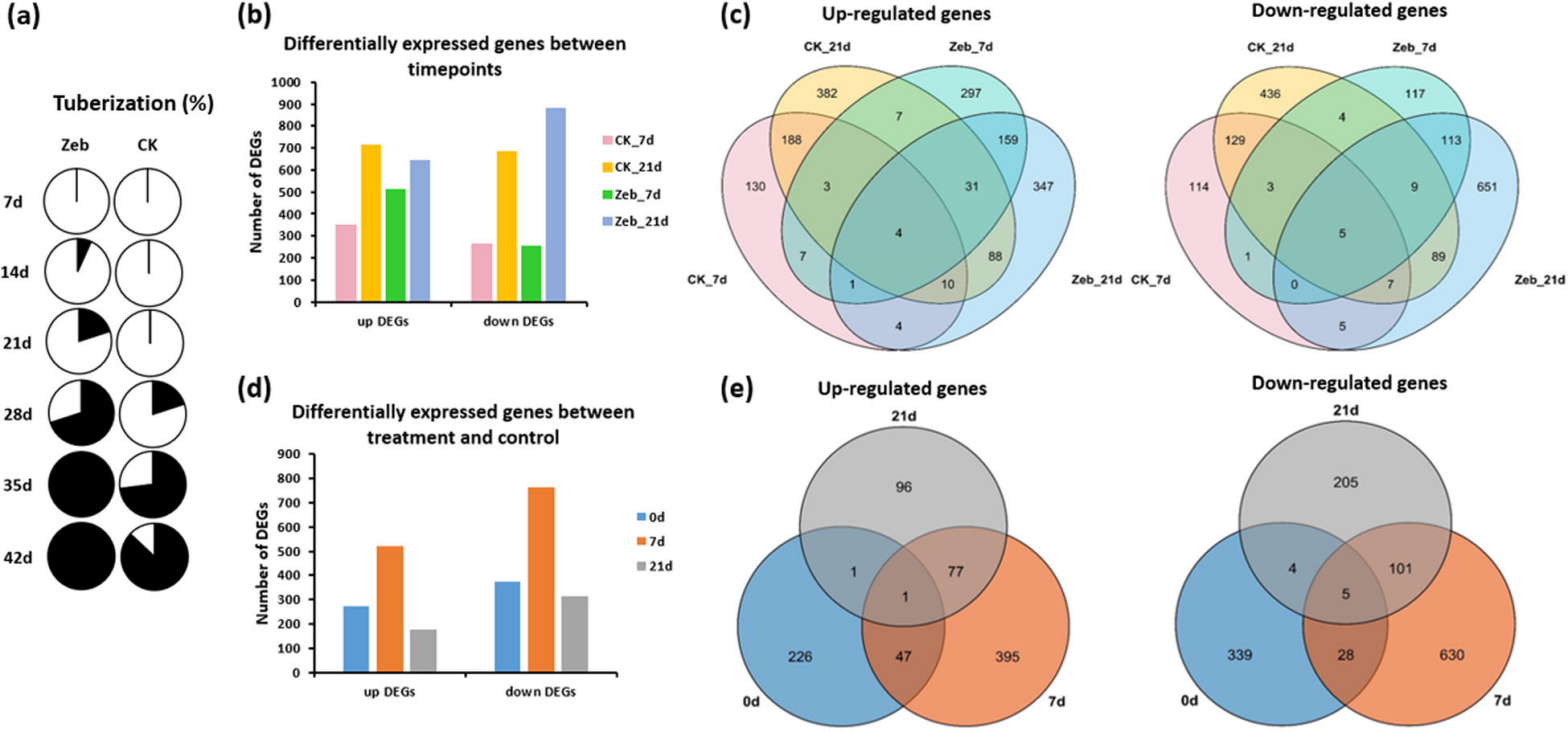
Comparative analysis of differentially expressed genes (DEGs) in the tuberization-induced process of the treatment (Zeb) and control (CK) groups of E26. **a** Proportion of the tuberized samples of the treatment (Zeb) and control (CK) groups at different timepoints (day) grown under short-day conditions. Black shows the percentage of tuberized samples. **b** Histogram showing the numbers of DEGs at 7 and 21 d relative to 0 d of the treatment (Zeb) group and 7 and 21 d relative to 0 d of the control (CK) group. **c** Venn diagrams showing overlaps among the up- and downregulated DEGs represented in (**b**). **d** Histogram showing the numbers of DEGs in the treatment (Zeb) group relative to the control (CK) group at 0, 7, and 21 d. **e** Venn diagrams showing overlaps among the up- and downregulated DEGs represented in (**d**)

To investigate whether DNA methylation affects gene expression during tuberization, we generated transcriptome profiles for the zebularine-treated and control groups of E26, with three biological replicates at four timepoints (0, 7, 21, and 28 d) under SD conditions. The Zeb treatment promoted early tuber formation at 14 d compared to 28 d in the control (Fig. 4a). A paired comparison of the RNA sequencing data was conducted for differentially expressed genes (DEGs) at 7 days (before the initiation of tuberization in the Zeb group) and 21 days (before the initiation of tuberization in the CK group) relative to 0 days. The numbers of up- and downregulated DEGs increased with the extension of SD from 7 to 21 d in both the Zeb and CK groups (Fig. 4b, Supplementary Table S7), suggesting that the global gene expression patterns were gradually altered during development and tuberization. It was further demonstrated that the numbers of up- and downregulated DEGs were obviously increased from 0 to 7 d and then dramatically declined from 7 to 21 d (Fig. 4d, Supplementary Table S7), indicating that the first 7 days could be a vital phase for tuber initiation. Venn diagrams showed that the identified DEGs, either at different timepoints or between different treatments, were greatly different with few overlaps (Fig. 4c, e), implying that DNA methylation may have temporal effects on specific genes during potato tuberization.

**Fig. 5:**
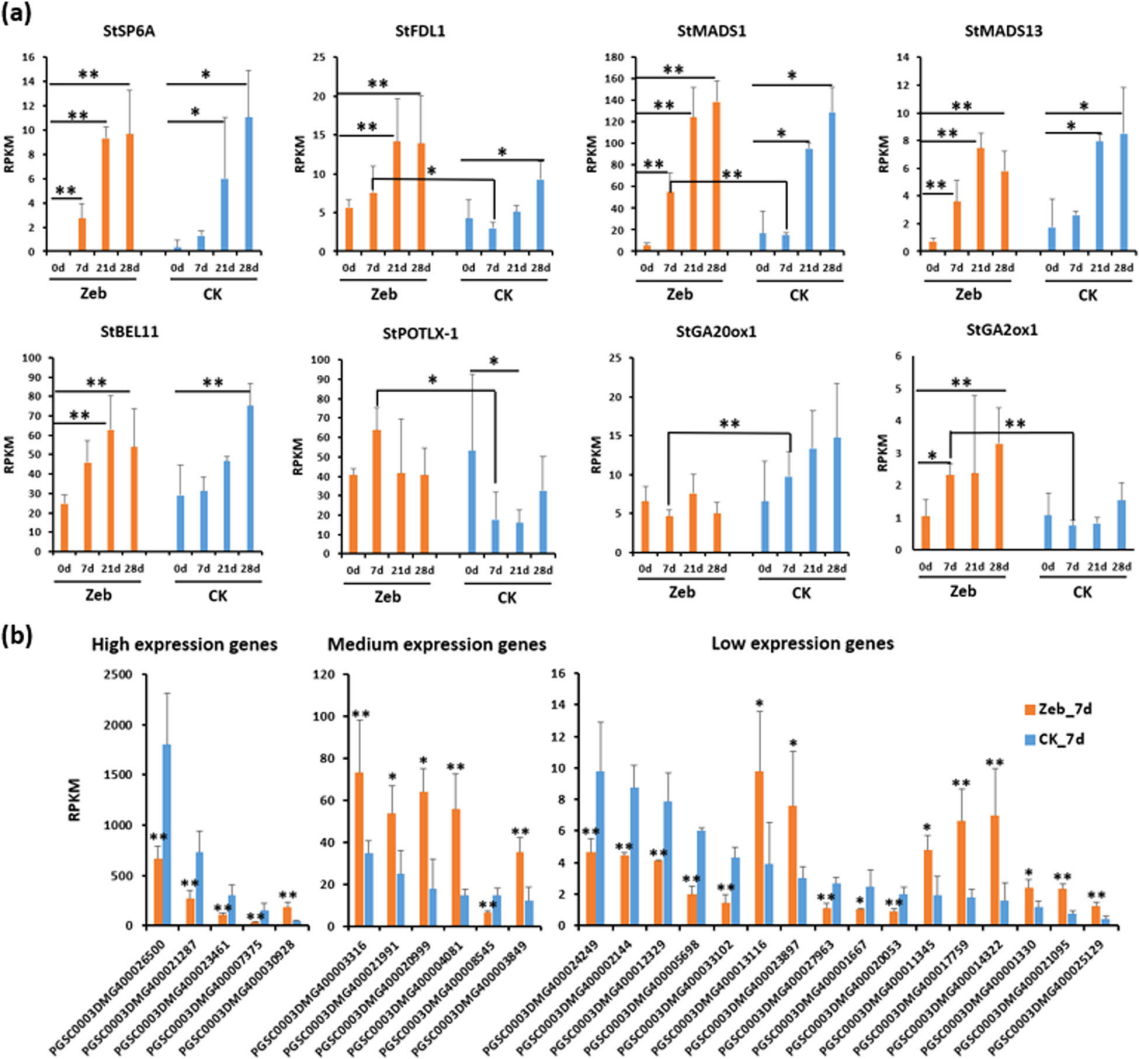
Tuberization-associated genes and photoperiod response genes that are differentially expressed in the treatment and control groups at different timepoints. **a** Transcript levels of reported tuberization-associated genes at 0, 7, 21, and 28 d in the Zeb and CK groups. **b** Transcript levels of photoperiod response genes at 7 d in the Zeb and CK groups. *P ≤ 0.05, ** *P* ≤ 0.01. Error bars indicate the standard error of three biological replicates

Furthermore, we dissected the transcript levels of 21 reported tuberization regulation genes (Supplementary Table S8) and 332 photoperiod response genes^39^ (Supplementary Table S9) at all timepoints in the Zeb and CK groups. The results showed that 8 tuberization regulation genes (*StSP6A, StFDL1, StMADS1, StMADS13, StBEL11, StPOTLX-1, StGA20ox1*, and *StGA2ox1*) were differentially expressed in the Zeb treatment and control groups at different timepoints (Fig. 5a). The tuberigen activation complex (TAC) elements *StSP6A* and *StFDL1*^40^ and their potential downstream target genes *StMADS1* and *StMADS13*^41^ were significantly upregulated starting at 7 d in the Zeb group and 21 d in the CK group (Fig. 5a). This finding is in accordance with the earlier tuber formation in the Zeb treatment group than in the control group (Fig. 4a), indicating that these genes were regulated by DNA methylation and resulted in early tuber formation. In addition, two key genes involved in the GA biosynthetic pathway, *StGA20ox1* (repress/delay tuberization)^42^ and *StGA2ox1* (induce tuberization)^43^, were downregulated and upregulated, respectively, in Zeb_7d in comparison with CK_7d, and the latter was also repressed relative to Zeb_0d (Fig. 5a), suggesting that the GA pathway may also be involved in early tuber initiation promoted by Zeb treatment.

In addition, 68 of the 332 reported photoperiod response genes were differentially expressed at different timepoints (Supplementary Table S10), including all 8 tuberization regulation genes described above. In particular, 27 of these 68 genes were differentially expressed in the vital phase of tuber initiation---Zeb_7d relative to CK_7d (14 upregulated genes and 13 downregulated genes in Zeb_7d) (Fig. 5b), including 2 MADS-box genes (*StMADS1* and MADS-box protein 17), 4 GA oxidase genes (*StGA2ox1*, *StGA20ox1*, GA20 oxidase, and gibberellin 3-oxidase), and other photoperiodic regulation genes (i.e., cryptochrome 1b, phytochrome kinase substrate, BEL14 protein, and flowering promoting factor-like 1) (Supplementary Table S10). These results further confirm that many photoperiodic regulatory genes respond to DNA methylation changes that may result in early tuber formation of the photoperiod-sensitive genotype.

### Correlation between DNA methylation and gene expression during photoperiodic tuberization

**Fig. 6:**
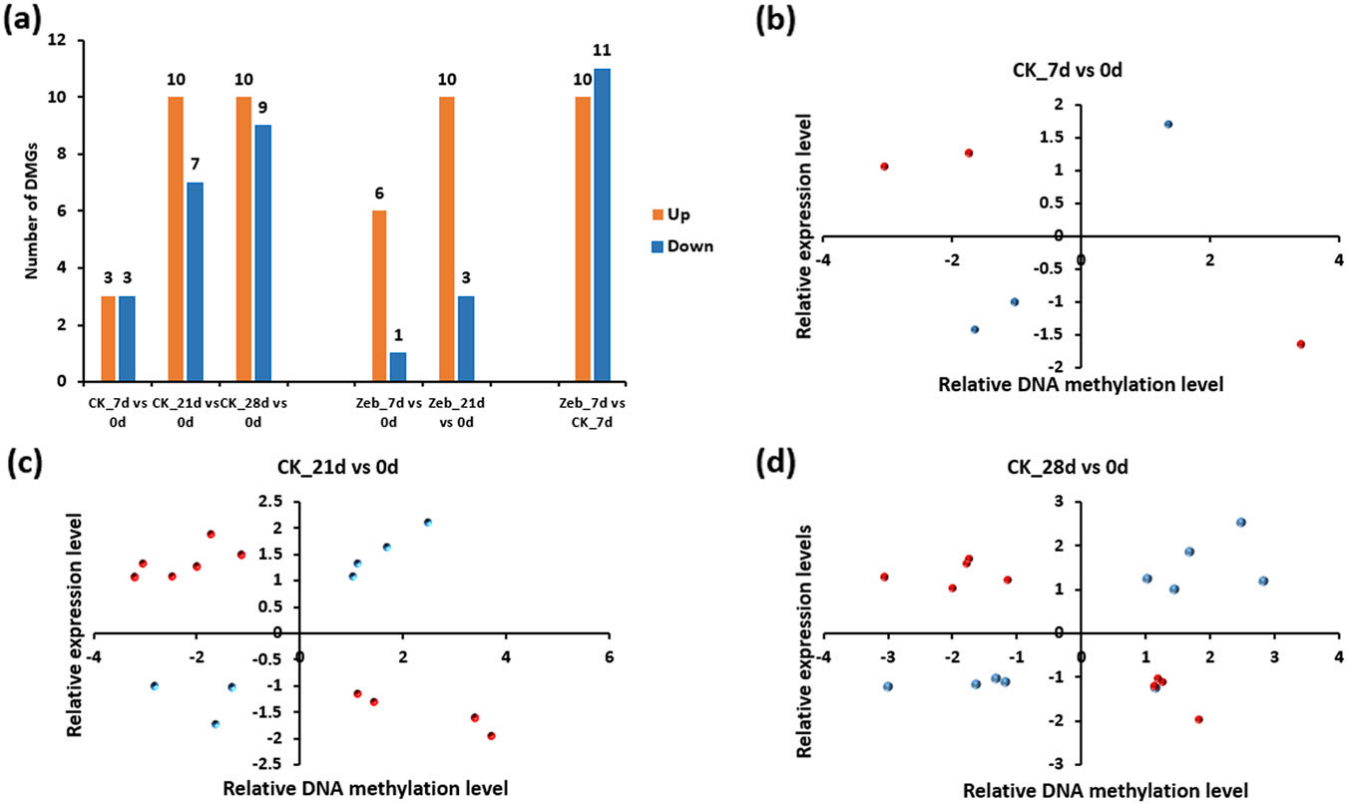
Relationship between DNA methylation and gene expression levels in the photoperiodic tuberization process. **a** Histogram shows the number of differentially methylated genes (DMGs) that were also differentially expressed in the Zeb treatment vs the control at each timepoint in E26. **b**–**d** The relationship between DNA methylation levels and their expression levels based on the genes overlapping with DMGs and DEGs in E26 at different timepoints. Red spots represent the genes that are negatively correlated with their DNA methylation levels (downregulated methylation levels accompanied by upregulated expression levels and upregulated methylation levels accompanied by downregulated expression levels), while blue spots represent genes with a positive correlation (downregulated methylation levels accompanied by downregulated expression levels and upregulated methylation levels accompanied by upregulated expression levels)

To investigate the association between the variations in DNA methylation and gene expression, we compared the DMGs and DEGs involved in the tuberization process of E26. A total of 52 DMGs (10.4%) were differentially expressed in the Zeb treatment and control at different timepoints (Supplementary Table S11). Relative to CK_0d, 6 DMGs were differentially expressed in CK_7d (3 upregulated and 3 downregulated genes in CK_7d), 17 were differentially expressed in CK_21d (10 upregulated and 7 downregulated genes in CK_21d), and 19 were differentially expressed in CK_28d (10 upregulated and 9 downregulated genes in CK_28d). Relative to Zeb_0d, 7 DMGs were differentially expressed in Zeb_7d (6 upregulated and 1 downregulated genes in Zeb_7d) and 13 were differentially expressed in Zeb_21d (10 upregulated and 3 downregulated genes in Zeb_21d). It was also found that 21 DMGs were differentially expressed in Zeb_7d compared to CK_7d (10 upregulated and 11 downregulated genes in Zeb_7d) (Fig. 6a). Among the DEGs that also exhibited differential methylation levels, most were upregulated in the Zeb group, indicating that DNA methylation inhibitor treatment activated the expression of many genes. In addition, most DMGs (89.6%) in E26 were not differentially expressed or had no expression in the treatment and control at different timepoints, which is similar to the results for floral development in Arabidopsis (19.1% of DMGs showed significant variations in gene expression)^16^ and fruit ripening in orange (29.4% of hyper-DMR-associated genes showed significant variations in gene expression)^18^. These findings confirm that the regulation of gene expression is just one of the ways that DNA methylation affects plant development, and there are also other important methods, such as genomic imprinting and transposon silencing^44,45^.

Based on the log_2_FoldChange values, we analyzed the relationships between the DNA methylation levels and their expression levels, focusing on the DMGs that were also differentially expressed at different timepoints in the control group (not including the DEGs in the Zeb group) because the DMGs were identified in the tuberization process under short-day conditions and not under Zeb treatment. In CK_7d relative to CK_0d, among the 6 DEGs that also exhibited differential methylation levels, 3 were negatively correlated (red spots) with their DNA methylation levels, and the other 3 showed a positive correlation (blue spots) (Fig. 6b). Similarly, in CK_21d, 10 of the 17 DEGs were negatively correlated with their DNA methylation levels, and 7 were positively correlated (Fig. 6c). In addition, in CK_28d, 10 of the 19 DEGs were negatively correlated with their DNA methylation levels, and 9 showed a positive relationship (Fig. 6d). In general, DNA methylation in promoters is normally associated with genes involved in transcriptional repression or silencing^46^. However, there is also evidence showing that highly methylated promoters could also occur in upregulated genes^47^. In addition, DNA methylation also occurs in the gene body, and little is known about its functions. Consistent with previous studies in fruit ripening^17,18^ and floral development^16^, our results show that DNA hypermethylation may have a positive role in regulating gene expression and may also cause transcriptional repression in potato tuber initiation, suggesting that DNA methylation patterns in plant development are complex.

## Discussion

The day-length (photoperiod) response is important for plants to perceive environmental cues and plays an essential role in plant development, including the regulation of flowering time^48^ and tuber formation^7^. DNA methylation has been reported to contribute to photoperiodic flowering regulation^15^ and plays significant roles in the domestication of important agricultural traits and adaptation to worldwide cultivation in crops. In the present study, we identified differentially methylated genes (DMGs) of potato during tuberization and dissected their relationship with photoperiodic tuberization genes. To our knowledge, it is complementary to systematically use epigenetic methods to approach the mechanism underlying potato adaptability to day length.

DNA methylomes for three floral periods (meristems, early flowers, and late flowers) in Arabidopsis have shown opposite changes in DNA methylation during floral development: many cytosine sites gained methylation from the meristem to the early flower stage, and many cytosine sites lost methylation from early to late flowering^16^. In our data, we found different DNA methylation modes in three tested clones: most DMGs gained DNA methylation in the photoperiod-insensitive clone E108 (tuberized well in both SD and LD photoperiods) and lost DNA methylation in the nontuberized clone E20 (no tuber formation under either SD or LD conditions), but in the photoperiod-sensitive clone E26 (only tuberized under SD conditions), the number of DMGs that gained and lost DNA methylation were nearly equal (Fig. 3a, Supplementary Table S6). Our results suggest that these 3 clones with totally different photoperiodic tuberization performances have distinct DNA methylation responses to short-day tuberization induction. In addition, few DMGs were shared by these clones (Fig. 3b), and genes involved in the photoperiodic tuberization-related pathway “circadian rhythm” (*Phytochrome F, Phytochrome B2, Flowering locus T JHL23J11.9 protein*, and *Transcription factor HY5*) were significantly enriched in the photoperiod-sensitive genotype E26 (Fig. 3c). Recently, *phytochrome F* (*StPHYF*) has been reported to play critical roles in potato photoperiod-dependent tuberization by stabilizing the StCOL1 protein to regulate the CO-FT tuberization pathway^8^. Our results may provide new insight into the role of *phytochrome F* in photoperiodic tuberization via epigenetic mechanisms.

Furthermore, DNA methylation influences flowering^15,16^ or the photoperiod sensitivity of plants^20^ mainly by modulating the expression of related genes. We also found that the DNA methylation inhibitor could advance tuber initiation in the photoperiod-sensitive genotype E26 by regulating the expression of the genes involved in the photoperiod and GA pathways (Fig. 5). In potato, StSP6A, an FT-like protein, was identified as a major component of tuberigen. Similar to florigen activation complex (FAC)-induced flowering in Arabidopsis^49,50^ and rice^51^, potato tuberization is regulated by the tuberigen activation complex (TAC), comprising StSP6A, St14-3-3s, and StFDL1^40^, through activation of the expression of downstream target genes, which might be MADS-box genes^41,47,48^. In our results, the TAC elements StSP6A and StFDL1^40^ and their potential downstream target genes StMADS1 and StMADS13^41^ were significantly upregulated in accordance with tuber initiation timepoints (Fig. 5a), indicating that these genes were regulated by DNA methylation and resulted in early tuber formation.

In addition to environmental cues (photoperiod), plant hormones, especially the action of gibberellins (GAs), have been implicated in different aspects of potato tuber formation. GA accumulation in the subapical stolon region will repress or delay tuber initiation^43^. Therefore, overexpression of *StGA20ox1*, a gene encoding a key enzyme (GA 20-oxidase1) in the GA biosynthetic pathway, resulted in late tuberization^42^. In contrast, overexpression of *StGA2ox1*, a gene encoding another key enzyme (GA 2-oxidase1) that can transform active GA into inactive GA, resulted in earlier tuberization than control plants^43^. In our data, two key genes involved in the GA biosynthetic pathway, *StGA20ox1* (repress/delay tuberization)^42^ and *StGA2ox1* (induce tuberization)^43^, were downregulated and upregulated, respectively, in the tuber initiation timepoint of the Zeb treatment group (Fig. 5a), suggesting that early tuber initiation promoted by DNA methylation inhibitors may also be regulated by the GA pathway.

There has long been crosstalk between the photoperiod and GA pathways in regulating tuberization, and powerful evidence for this consideration is that the photoperiodic regulation gene *StBEL5* and its protein partner POTH1 affect the tuberization of potato by negatively regulating the expression of *StGA20ox1* and then mediating GA levels in stolon tips^52,53^. In addition, induction of *StSP6A* activates *StGA2ox1* gene expression in the stolon^9^, and *StSP6A* and several induced genes, including *StGA2ox1*, are activated by overexpression of *StBEL5* in the stolon^11^. In our data, transcriptome levels of *StBEL5* or *StPOTH1* showed no difference in the Zeb treatment and control groups at all timepoints, but *StPOTH1* showed different methylation levels in the tuberization process in E26, suggesting that expression changes of GA pathway-related genes might be mediated by the DNA methylation modification of *StPOTH1*. Taken together, these results suggest that a key element (possibly *phytochrome F, StPOTH1*, or some other genes in the 52 DMGs/DEGs, Supplementary Table S11) involved in the crosstalk between the photoperiod and GA pathway was regulated by DNA methylation, which modulated the downstream target genes to influence tuberization in response to different day lengths.

The photoperiod insensitivity of tuberization is vital for the spread of potato (*Solanum tuberosum* L.) cultivars from their original area in the South American Andes (short-day) to worldwide geographical ranges. Therefore, photoperiodic tuberization has been regarded as a key issue for understanding the adaptability of potato to diverse day-length conditions, and the *StCDF1* gene, with natural variation in controlling tuberization under long days, has been considered to be an important allelic variant for long-day adaptation in potato^10,54^. Here, we report the effect of Zeb on the tuberization of three distinct potato genotypes (E26, E20, and E108) that have various photoperiodic sensitivities. However, neither different photoperiods nor Zeb treatment affected the tuberization of E108. Gene expression analysis of allelic CDF1 variants showed that there was no significant difference in the gene expression pattern among these three genotypes, and the genetic structure of the CDF1 variants was quite similar^55^. In other words, there could be other genes/pathways that contribute to long-day tuberization. Recently, Gutaker et al. traced the demographic and adaptive history of potato introduction to Europe by sequencing landraces, modern cultivars, and historical herbarium samples^39^. Their results showed that none of the long-day-adaptive alleles in the *StCDF1* gene were found in the oldest European samples of Andean descent or in Andean landraces, which means that the European individuals from 1650 to 1750 were not long-day adapted through allelic variants of the *StCDF1* gene. Instead, the authors identified 8 candidate genes putatively involved in long-day preadaptation, including 2 gibberellin 20-oxidase genes, a MADS-box gene targeted by SP6A florigen, and a MADS-box transcript factor *FBP28*. At the same time, Li et al. reported a comprehensive assessment of wild and cultivated potato species based on the genomic analysis of 201 accessions of *Solanum* section *Petota*^30^. They identified 609 genes under selection and associated with artificial selection or local adaptation, including two critical tuberization genes, *GA20ox-1* and *CONSTANS*. All these findings suggest that the photoperiodic tuberization and GA pathways play critical roles in potato domestication and adaptation for worldwide cultivation. In the present study, we provided lines of evidence of tuberization modulation by Zeb in a SD-sensitive genotype of potato through methylation of tuberization-related genes involved in the photoperiod and GA pathways. Our results provide a new perspective for understanding the relationship between photoperiod-dependent and GA-regulated tuberization. Uncovering the epigenomic signatures of these pathways will greatly promote the development of potato breeding and adaptation for new environments.

In summary, we found that a DNA methylation inhibitor (zebularine) could promote tuber initiation in strict short-day genotypes by regulating the expression of key genes involved in the photoperiod and GA pathways. Whole-genome DNA methylation sequencing showed that the photoperiod-sensitive and photoperiod-insensitive genotypes had distinct DNA methylation modes, sharing only a few differentially methylated genes. Comparison of the DNA methylation levels and transcriptome levels identified 52 candidate genes regulated by DNA methylation that were predicted to be involved in photoperiodic tuberization. We discussed the possibility that DNA methylation is involved in the crosstalk between the photoperiod and GA pathways in regulating tuberization, although the key genes remain to be exploited. To explain this result, in addition to DNA methylation, Zeb may also have an effect on histone methylation. In mammals and cancer research, Zeb is reported as a stable DNA demethylating agent and is the first drug that can reactivate an epigenetically silenced gene by oral administration compared to other similar types of drugs^56^. Recent research on cardiomyogenic differentiation of human amniotic fluid-derived mesenchymal stem cells confirmed that Zeb can potentially be used as a cardiomyogenic differentiation inducer in AF-MSCs, which could cause various genetic and epigenetic changes resulting in global chromatin remodeling^57^. In plants, Zeb treatment led to an increased spikelet number in bread wheat by repressing the floral promoter FT-B1^58^. Therefore, we speculate that Zeb treatment of potato in our study may cause a more relaxed chromatin conformation, resulting in a general induction of gene expression. However, further investigation with more potato genotypes will be needed to support this.

## Supplementary information

Supplementary Table S12

Supplementary Figure S1-S6

Supplementary Table S1-S7

Supplementary Table S8

Supplementary Table S9

Supplementary Table S10

Supplementary Table S11

Dataset 1

Dataset 2
