# Supplementary material for: DNA methylation affects photoperiodic tuberization in potato (Solanum tuberosum L.) by mediating the expression of genes related to the photoperiod and GA pathways
Source: Hortic Res. 2021 Sep 1;8:181. doi: 10.1038/s41438-021-00619-7 (PMC8408180; doi:10.1038/s41438-021-00619-7)
Supplement: Supplementary file 1 — Supplementary Table S12 [file 41438_2021_619_MOESM1_ESM.pdf]

Supplementary Table S12: List of differentially methylated genes shared by E26, E20 and/or E108 (genomic coordinates are based on Solanum tuberosum genome PGSC v.4.04).

Gene_ID	Note	PGSC_functional_annotation	nr_annotation	Swiss-Prot_annotation
PGSC0003DMG400029335	common in E26/E20/E108	Plant synaptotagmin	synaptotagmin-1-like	Synaptotagmin-1
PGSC0003DMG400034650	common in E26/E20/E108	Gene of unknown function	-	-
PGSC0003DMG400034715	common in E26/E20/E108	Conserved gene of unknown function	hypothetical protein	-
PGSC0003DMG401016979	common in E26/E20/E108	Leucine Rich Repeat family protein	TMV resistance protein N-like	TMV resistance protein N
PGSC0003DMG403016979	common in E26/E20/E108	NL27	TMV resistance protein N-like	TMV resistance protein N
PGSC0003DMG400001092	common in E26/E20	UDP-glucoronosyl/UDP-glucosyl transferase family protein	UDP-glycosyltransferase 89B1-like	UDP-glycosyltransferase 89B1
PGSC0003DMG400004632	common in E26/E20	UDP-glucuronic acid decarboxylase 2	UDP-glucuronic acid decarboxylase 4-like	UDP-glucuronic acid decarboxylase 4
PGSC0003DMG400005369	common in E26/E20	Conserved gene of unknown function	hypothetical protein	-
PGSC0003DMG400005991	common in E26/E20	Membrane associated ring finger 1,8	uncharacterized protein	-
PGSC0003DMG400007177	common in E26/E20	Peptidylprolyl isomerase	70 kDa peptidyl-prolyl isomerase-like isoform X1	Peptidyl-prolyl cis-trans isomerase FKBP65
PGSC0003DMG400010677	common in E26/E20	Heat shock 70 kDa protein, mitochondrial	heat shock 70 kDa protein, mitochondrial	Heat shock 70 kDa protein, mitochondrial
PGSC0003DMG400013680	common in E26/E20	Pectinesterase	probable pectinesterase/pectinesterase inhibitor 12	Probable pectinesterase/pectinesterase inhibitor 12
PGSC0003DMG400014616	common in E26/E20	Conserved gene of unknown function	protein N-lysine methyltransferase METTL21A	Protein N-lysine methyltransferase METTL21A
PGSC0003DMG400015576	common in E26/E20	Nuclease	uncharacterized protein	Uncharacterized protein Mb2253c
PGSC0003DMG400016726	common in E26/E20	Pyrophosphate--fructose 6-phosphate 1-phosphotransferase subunit beta	uncharacterized protein	Pyrophosphate--fructose 6-phosphate 1-phosphotransferase subunit beta
PGSC0003DMG400020151	common in E26/E20	SET domain protein	uncharacterized protein	Histone-lysine N-methyltransferase SUVR4
PGSC0003DMG400027020	common in E26/E20	Alpha-L-fucosidase 2	GDSL esterase/lipase	GDSL esterase/lipase At3g27950
PGSC0003DMG400035949	common in E26/E20	Gene of unknown function	uncharacterized protein	-
PGSC0003DMG400036476	common in E26/E20	Gene of unknown function	uncharacterized protein	-
PGSC0003DMG400036621	common in E26/E20	Conserved gene of unknown function	uncharacterized protein	-
PGSC0003DMG400039701	common in E26/E20	Gene of unknown function	-	-
PGSC0003DMG400040176	common in E26/E20	Gag-pol protein	uncharacterized protein	-
PGSC0003DMG400040880	common in E26/E20	Gene of unknown function	-	-
PGSC0003DMG400045568	common in E26/E20	Gene of unknown function	-	-
PGSC0003DMG400046143	common in E26/E20	Gene of unknown function	-	-

PGSC0003DMG401000361	common in E26/E20	RNA-dependent RNA polymerase family protein	probable RNA-dependent RNA polymerase 5	Probable RNA-dependent RNA polymerase 5
PGSC0003DMG401022603	common in E26/E20	ATP binding / protein binding / transmembrane receptor	TMV resistance protein N-like	TMV resistance protein N
PGSC0003DMG401028453	common in E26/E20	ATP binding protein	probable LRR receptor-like serine/threonine-protein kinase RFK1 isoform X1	Probable LRR receptor-like serine/threonine-protein kinase RFK1
PGSC0003DMG400000063	common in E26/E108	Heavy metal-associated domain containing protein	pollen-specific leucine-rich repeat extensin-like protein 1	Heavy metal-associated isoprenylated plant protein 39
PGSC0003DMG400000781	common in E26/E108	Extensin	extensin-3-like isoform X1	-
PGSC0003DMG400001077	common in E26/E108	Condensin complex subunit 2	condensin complex subunit 2	Condensin complex subunit 2
PGSC0003DMG400004236	common in E26/E108	Pentatricopeptide repeat-containing protein	pentatricopeptide repeat-containing protein	Pentatricopeptide repeat-containing protein At2g16880
PGSC0003DMG400004417	common in E26/E108	'chromo' domain containing protein	chromo' domain containing protein	-
PGSC0003DMG400005016	common in E26/E108	Pathogenesis-induced protein	abscisic acid receptor PYL8-like	Abscisic acid receptor PYL9
PGSC0003DMG400006257	common in E26/E108	Basic helix-loop-helix family protein	transcription factor bHLH140	Transcription factor bHLH140
PGSC0003DMG400007998	common in E26/E108	Organellar single-stranded	protein OSB1, mitochondrial-like	Protein OSB1, mitochondrial
PGSC0003DMG400009182	common in E26/E108	H+/Ca2+ exchanger 2	vacuolar cation/proton exchanger 3-like isoform X1	Vacuolar cation/proton exchanger 2
PGSC0003DMG400009620	common in E26/E108	Cytochrome P450 71D6	cytochrome P450 71D6	Cytochrome P450 71D6
PGSC0003DMG400012115	common in E26/E108	Calmodulin binding protein	protein IQ-DOMAIN 1-like	Protein IQ-DOMAIN 14
PGSC0003DMG400014622	common in E26/E108	2-isopropylmalate synthase A	2-isopropylmalate synthase A	2-isopropylmalate synthase A
PGSC0003DMG400019975	common in E26/E108	Ankyrin repeat-containing protein	ankyrin repeat-containing protein	Ankyrin repeat-containing protein At2g01680
PGSC0003DMG400022487	common in E26/E108	DNAJ heat shock N-terminal domain-containing protein	uncharacterized protein	Chaperone protein DnaJ
PGSC0003DMG400023490	common in E26/E108	Pleiotropic drug resistance protein 2	pleiotropic drug resistance protein 2-like	Pleiotropic drug resistance protein 2
PGSC0003DMG400024641	common in E26/E108	24 kDa seed maturation protein	reticulon-like protein B2	-
PGSC0003DMG400025230	common in E26/E108	Potassium transporter	potassium transporter 4-like	Potassium transporter 4
PGSC0003DMG400027288	common in E26/E108	Glycosyltransferase	EGF domain-specific O-linked N-acetylglucosamine transferase	-
PGSC0003DMG400029702	common in E26/E108	Smg-7	protein SMG7L	Protein SMG7L
PGSC0003DMG400031206	common in E26/E108	RAB7A	ras-related protein Rab7-like	Ras-related protein Rab7
PGSC0003DMG400034324	common in E26/E108	Conserved gene of unknown function	uncharacterized protein	-
PGSC0003DMG400036025	common in E26/E108	Gene of unknown function	mucin-5AC-like	-
PGSC0003DMG400036344	common in E26/E108	Ribonuclease H	uncharacterized protein	-
PGSC0003DMG400036669	common in E26/E108	Gene of unknown function	-	-
PGSC0003DMG400038760	common in E26/E108	Gene of unknown function	uncharacterized protein	-

PGSC0003DMG400041415	common in E26/E108	Conserved gene of unknown function	mucin-5AC-like	-
PGSC0003DMG400044663	common in E26/E108	Conserved gene of unknown function	uncharacterized protein	-
PGSC0003DMG400045142	common in E26/E108	Gene of unknown function	-	-
PGSC0003DMG401023696	common in E26/E108	NPR1-interactor protein 1	TGACG-sequence-specific DNA-binding protein TGA-2.1-like	TGACG-sequence-specific DNA-binding protein TGA-2.1
PGSC0003DMG402006875	common in E26/E108	Conserved gene of unknown function	probable carbohydrate esterase	Probable carbohydrate esterase At4g34215
PGSC0003DMG402012957	common in E26/E108	Protein CPR-5	protein CPR-5	Protein CPR-5
PGSC0003DMG402017733	common in E26/E108	Myb-transcription factor	transcription factor TFIIIB component B" isoform X2	Transcription factor TFIIIB component B"
PGSC0003DMG402025057	common in E26/E108	Short-chain dehydrogenase/reductase family protein	dehydrogenase/reductase SDR family member 12	Dehydrogenase/reductase SDR family member 12
PGSC0003DMG400000905	common in E20/E108	RNA binding protein	DIS3-like exonuclease 2	DIS3-like exonuclease 2
PGSC0003DMG400001768	common in E20/E108	Calmodulin binding protein	protein IQ-DOMAIN 1-like isoform X2	Protein IQ-DOMAIN 1
PGSC0003DMG400002301	common in E20/E108	Bifunctional lysine-ketoglutarate reductase/saccharopine dehydrogenase	alpha-aminoadipic semialdehyde synthase isoform X2	Alpha-aminoadipic semialdehyde synthase
PGSC0003DMG400002318	common in E20/E108	DNA binding protein	cell wall protein RBR3	-
PGSC0003DMG400002999	common in E20/E108	Conserved gene of unknown function	protein FAF-like, chloroplastic	Protein FAF-like, chloroplastic
PGSC0003DMG400003076	common in E20/E108	SH3 domain-containing protein 3	SH3 domain-containing protein 3	SH3 domain-containing protein 3
PGSC0003DMG400003357	common in E20/E108	CONSTANS interacting protein 2b	nuclear transcription factor Y subunit C-9-like	Nuclear transcription factor Y subunit C-9
PGSC0003DMG400004886	common in E20/E108	Threonine synthase, chloroplastic	threonine synthase, chloroplastic-like	Threonine synthase, chloroplastic
PGSC0003DMG400005092	common in E20/E108	F-box and wd40 domain protein	putative E3 ubiquitin-protein ligase LIN isoform X1	Putative E3 ubiquitin-protein ligase LIN-1
PGSC0003DMG400007843	common in E20/E108	Ethylene receptor 1	ethylene receptor 1 isoform X1	Ethylene receptor 1
PGSC0003DMG400008442	common in E20/E108	SCL domain class transcription factor	scarecrow-like protein 13	Scarecrow-like protein 13
PGSC0003DMG400008947	common in E20/E108	Desacetoxyvindoline 4-hydroxylase	1-aminocyclopropane-1-carboxylate oxidase homolog 1-like	1-aminocyclopropane-1-carboxylate oxidase homolog 1
PGSC0003DMG400011515	common in E20/E108	Gene of unknown function	-	-
PGSC0003DMG400011607	common in E20/E108	Conserved gene of unknown function	protein BIG GRAIN 1-like B	Protein BIG GRAIN 1-like A
PGSC0003DMG400012063	common in E20/E108	Conserved gene of unknown function	uncharacterized protein	-
PGSC0003DMG400012752	common in E20/E108	Forkhead-associated domain-containing protein	rootletin isoform X2	-
PGSC0003DMG400012960	common in E20/E108	Conserved gene of unknown function	tobamovirus multiplication protein 1-like isoform X2	-

PGSC0003DMG400013309	common in E20/E108	NADH-ubiquinone oxidoreductase 75 kDa subunit, mitochondrial	NADH dehydrogenase iron-sulfur protein 1, mitochondrial	NADH dehydrogenase [ubiquinone] iron-sulfur protein 1, mitochondrial
PGSC0003DMG400013960	common in E20/E108	Gene of unknown function	-	-
PGSC0003DMG400013970	common in E20/E108	3-dehydroquinate synthase	3-dehydroquinate synthase homolog	-
PGSC0003DMG400014893	common in E20/E108	U1 small nuclear ribonucleoprotein 70 kDa	U1 small nuclear ribonucleoprotein 70 kDa isoform X1	U1 small nuclear ribonucleoprotein 70 kDa
PGSC0003DMG400017219	common in E20/E108	Pentatricopeptide repeat-containing protein	putative pentatricopeptide repeat-containing protein	Putative pentatricopeptide repeat-containing protein At3g23330
PGSC0003DMG400018010	common in E20/E108	Poly(RC)-binding protein	RNA-binding KH domain-containing protein PEPPER-like	RNA-binding KH domain-containing protein PEPPER
PGSC0003DMG400019410	common in E20/E108	Heavy metal-associated domain containing protein	glutamic acid-rich protein-like	-
PGSC0003DMG400020086	common in E20/E108	26S proteasome subunit 4	26S proteasome regulatory subunit 4 homolog A	26S proteasome regulatory subunit 4 homolog A
PGSC0003DMG400021726	common in E20/E108	Furin	uncharacterized protein	Protein HEAT-STRESS-ASSOCIATED 32
PGSC0003DMG400022547	common in E20/E108	ATP binding protein	protein HYPER-SENSITIVITY-RELATED 4-like	Protein HYPER-SENSITIVITY-RELATED 4
PGSC0003DMG400024483	common in E20/E108	Structural constituent of ribosome	39S ribosomal protein L47, mitochondrial	39S ribosomal protein L47, mitochondrial
PGSC0003DMG400025780	common in E20/E108	Transcription factor bHLH95	transcription factor bHLH95-like	Transcription factor bHLH95
PGSC0003DMG400029400	common in E20/E108	Cysteine protease 14	xylem cysteine proteinase 1-like	Cysteine protease XCP1
PGSC0003DMG400030718	common in E20/E108	Conserved gene of unknown function	hypothetical protein	-
PGSC0003DMG400031136	common in E20/E108	Sur2 hydroxylase/desaturase	sphinganine C4-monooxygenase 1-like	Sphinganine C4-monooxygenase 2
PGSC0003DMG400038870	common in E20/E108	Gene of unknown function	-	-
PGSC0003DMG400038894	common in E20/E108	Gene of unknown function	MICOS complex subunit MIC60-like	-
PGSC0003DMG400039423	common in E20/E108	Gene of unknown function	-	-
PGSC0003DMG400039598	common in E20/E108	Gag-pol polyprotein	uncharacterized protein	-
PGSC0003DMG400040078	common in E20/E108	Polyprotein protein	uncharacterized protein	-
PGSC0003DMG400041847	common in E20/E108	Ubiquitin	uncharacterized protein	-
PGSC0003DMG400041940	common in E20/E108	Conserved gene of unknown function	uncharacterized protein	-
PGSC0003DMG400042636	common in E20/E108	Membrane protein	allergen Asp f 7 homolog	-
PGSC0003DMG400042643	common in E20/E108	Ankyrin repeat-rich protein	hypothetical protein	-
PGSC0003DMG400043253	common in E20/E108	Integrase core domain containing protein	cysteine proteinase 4-like	-
PGSC0003DMG400043902	common in E20/E108	Gene of unknown function	-	-

PGSC0003DMG400044516	common in E20/E108	Gene of unknown function	uncharacterized protein	-
PGSC0003DMG400044794	common in E20/E108	Gene of unknown function	-	-
PGSC0003DMG400045803	common in E20/E108	Conserved gene of unknown function	hypothetical protein	-
PGSC0003DMG400046137	common in E20/E108	'chromo' domain containing protein	uncharacterized protein	-
PGSC0003DMG400046863	common in E20/E108	Gene of unknown function	-	-
PGSC0003DMG401022324	common in E20/E108	Conserved gene of unknown function	uncharacterized protein	-
PGSC0003DMG402015935	common in E20/E108	Aminoadipic semialdehyde synthase	alpha-aminoadipic semialdehyde synthase-like isoform X2	Alpha-aminoadipic semialdehyde synthase
