# Supplementary material for: DNA methylation affects photoperiodic tuberization in potato (Solanum tuberosum L.) by mediating the expression of genes related to the photoperiod and GA pathways
Source: Hortic Res. 2021 Sep 1;8:181. doi: 10.1038/s41438-021-00619-7 (PMC8408180; doi:10.1038/s41438-021-00619-7)
Supplement: Supplementary file 2 — Supplementary Figure S1-S6 [file 41438_2021_619_MOESM2_ESM.pptx]

## Slide 1
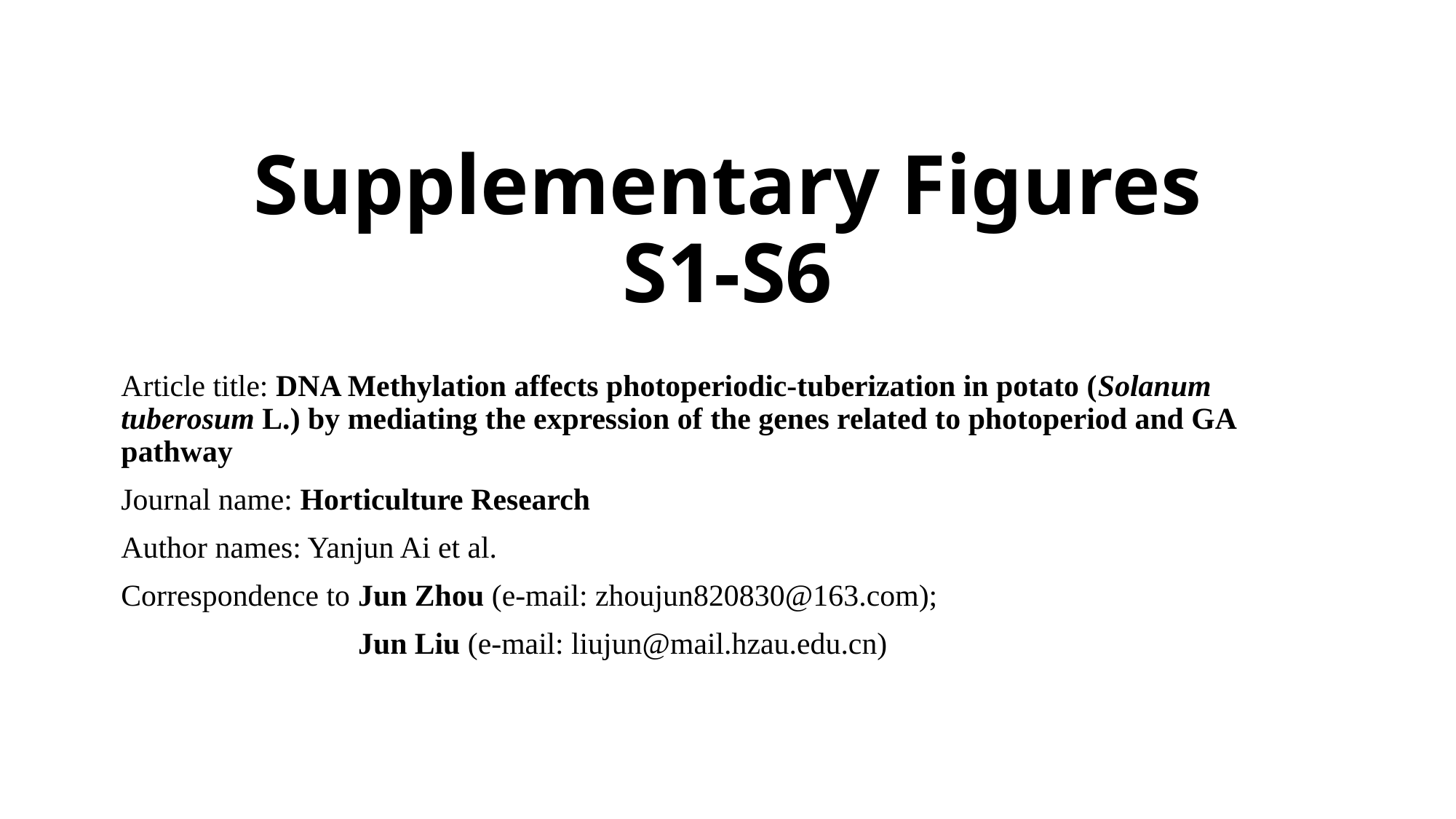

# Supplementary Figures S1-S6
Article title: DNA Methylation affects photoperiodic-tuberization in potato (Solanum tuberosum L.) by mediating the expression of the genes related to photoperiod and GA pathway
Journal name: Horticulture Research
Author names: Yanjun Ai et al.
Correspondence to Jun Zhou (e-mail: zhoujun820830@163.com);
 Jun Liu (e-mail: liujun@mail.hzau.edu.cn)

## Slide 2
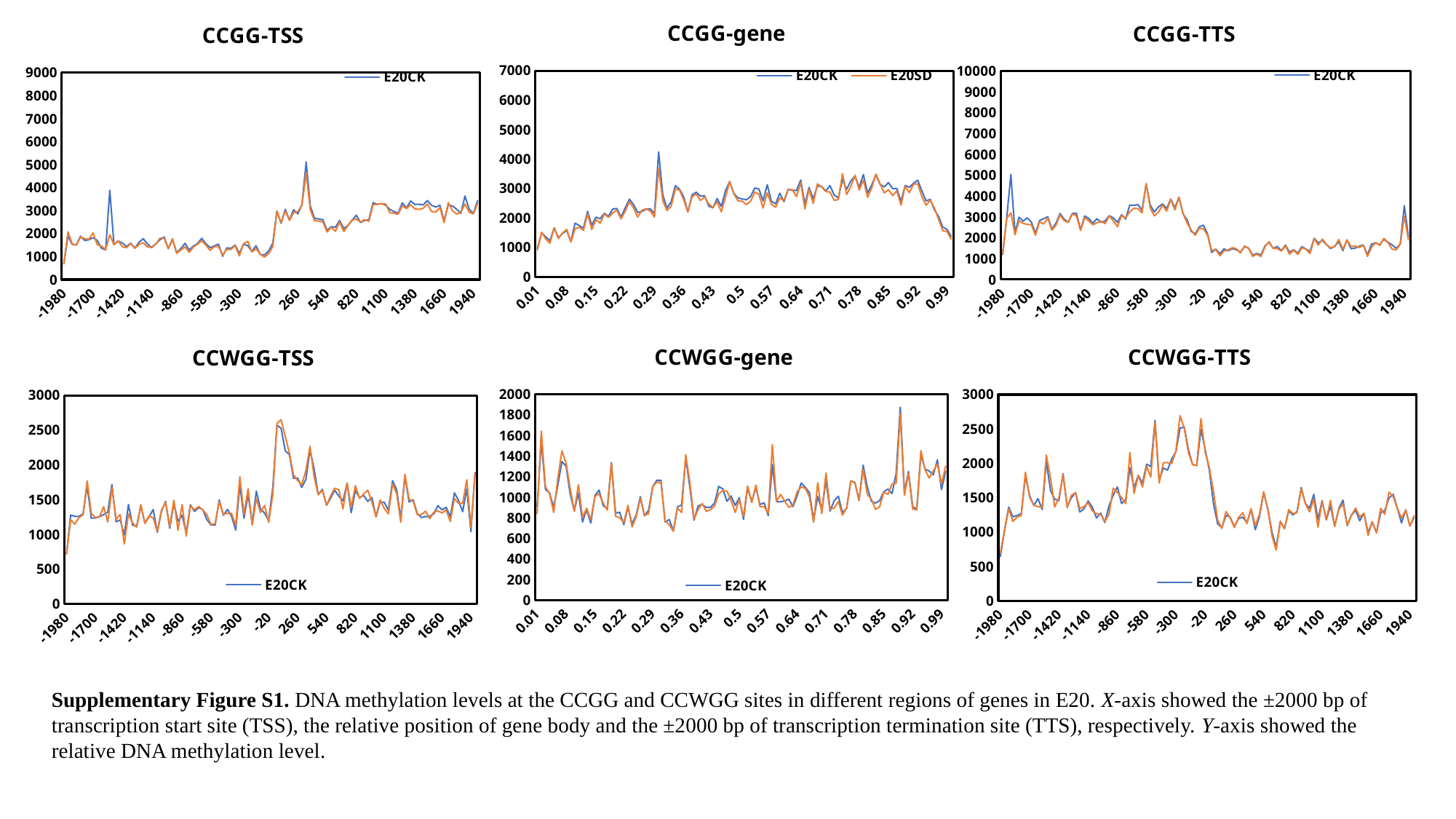

### Chart: CCGG-gene
| Category | E20CK | E20SD |
|---|---|---|
| 0.01 | 973.17 | 908.75 |
| 0.02 | 1485.35 | 1510.83 |
| 0.03 | 1350.74 | 1277.04 |
| 0.04 | 1218.55 | 1131.84 |
| 0.05 | 1657.23 | 1667.97 |
| 0.06 | 1323.41 | 1298.51 |
| 7.0000000000000007E-2 | 1467.16 | 1482.32 |
| 0.08 | 1569.58 | 1607.61 |
| 0.09 | 1200.71 | 1174.77 |
| 0.1 | 1817.07 | 1631.62 |
| 0.11 | 1736.81 | 1675.42 |
| 0.12 | 1644.6 | 1572.3 |
| 0.13 | 2217.42 | 2130.42 |
| 0.14000000000000001 | 1743.8 | 1602.01 |
| 0.15 | 2023.54 | 1941.74 |
| 0.16 | 1967.8 | 1813.2 |
| 0.17 | 2153.33 | 2128.19 |
| 0.18 | 2049.9 | 2014.35 |
| 0.19 | 2293.97 | 2165.54 |
| 0.2 | 2317.7 | 2255.21 |
| 0.21 | 2021.54 | 1963.71 |
| 0.22 | 2339.79 | 2224.89 |
| 0.23 | 2633.34 | 2547.99 |
| 0.24 | 2450.93 | 2335.56999999999 |
| 0.25 | 2178.14 | 2027.38 |
| 0.26 | 2216.08 | 2257.79 |
| 0.27 | 2291.44 | 2306.41 |
| 0.28000000000000003 | 2301.96 | 2226.66 |
| 0.28999999999999998 | 2149.78 | 2024.06 |
| 0.3 | 4240.22 | 3687.98999999999 |
| 0.31 | 2769.69 | 2576.45999999999 |
| 0.32 | 2327.5 | 2245.96 |
| 0.33 | 2541.07 | 2396.42 |
| 0.34 | 3086.99 | 2975.49999999999 |
| 0.35 | 2970.59 | 2941.84999999999 |
| 0.36 | 2711.82 | 2611.28 |
| 0.37 | 2196.1 | 2198.57 |
| 0.38 | 2782.97 | 2713.89 |
| 0.39 | 2868.64 | 2808.00999999999 |
| 0.4 | 2738.81 | 2588.74999999999 |
| 0.41 | 2748.62 | 2714.23 |
| 0.42 | 2394.93 | 2466.80999999999 |
| 0.43 | 2337.04 | 2347.43 |
| 0.44 | 2654.32 | 2549.09 |
| 0.45 | 2386.05 | 2204.74 |
| 0.46 | 2920.85 | 2704.28 |
| 0.47 | 3231.41 | 3225.23 |
| 0.48 | 2825.15 | 2813.94 |
| 0.49 | 2673.74 | 2571.62 |
| 0.5 | 2637.12 | 2561.88999999999 |
| 0.51 | 2611.17 | 2450.09999999999 |
| 0.52 | 2720.69 | 2552.7 |
| 0.53 | 3012.01 | 2871.87 |
| 0.54 | 2979.74 | 2801.72 |
| 0.55000000000000004 | 2571.93 | 2330.09 |
| 0.56000000000000005 | 3124.22 | 2848.09999999999 |
| 0.56999999999999995 | 2557.58 | 2458.98 |
| 0.57999999999999996 | 2477.3 | 2361.95999999999 |
| 0.59 | 2837.3 | 2672.04 |
| 0.6 | 2545.61 | 2583.85999999999 |
| 0.61 | 2963.88 | 2973.99999999999 |
| 0.62 | 2938.62 | 2949.0 |
| 0.63 | 2925.32 | 2719.87 |
| 0.64 | 3282.28 | 3198.88999999999 |
| 0.65 | 2464.68 | 2300.83 |
| 0.66 | 3031.89 | 2973.26 |
| 0.67 | 2642.04 | 2487.16 |
| 0.68 | 3080.28 | 3151.13 |
| 0.69 | 3065.59 | 3049.64 |
| 0.7 | 2907.87 | 2888.78 |
| 0.71 | 3092.88 | 2877.95 |
| 0.72 | 2780.12 | 2595.35 |
| 0.73 | 2671.79 | 2617.65999999999 |
| 0.74 | 3301.51 | 3496.77999999999 |
| 0.75 | 2968.8 | 2789.16999999999 |
| 0.76 | 3253.42 | 3068.71 |
| 0.77 | 3416.52 | 3442.28999999999 |
| 0.78 | 3028.76 | 2943.67999999999 |
| 0.79 | 3468.77 | 3278.97999999999 |
| 0.8 | 2842.87 | 2697.53999999999 |
| 0.81 | 3115.67 | 3012.96999999999 |
| 0.82 | 3457.22 | 3486.07999999999 |
| 0.83 | 3137.13 | 3150.68 |
| 0.84 | 3048.49 | 2847.29999999999 |
| 0.85 | 3195.31 | 2955.15 |
| 0.86 | 2988.72 | 2755.06999999999 |
| 0.87 | 2995.05999999999 | 2911.76999999999 |
| 0.88 | 2560.35 | 2430.76 |
| 0.89 | 3097.85 | 3065.44999999999 |
| 0.9 | 3035.06 | 2853.57999999999 |
| 0.91 | 3171.49 | 3142.26 |
| 0.92 | 3277.96 | 3152.59 |
| 0.93 | 2911.98 | 2723.61 |
| 0.94 | 2573.97 | 2427.47 |
| 0.95 | 2626.96 | 2598.29999999999 |
| 0.96 | 2271.03 | 2327.75999999999 |
| 0.97 | 2043.14 | 1942.46 |
| 0.98 | 1681.06 | 1561.99 |
| 0.99 | 1609.18 | 1535.63 |
| 1 | 1369.26 | 1271.86 |
### Chart: CCGG-TTS
| Category | E20CK | E20SD |
|---|---|---|
| -1980 | 1196.88 | 1241.89 |
| -1940 | 2999.99 | 2931.75999999999 |
| -1900 | 5028.04 | 3196.67999999999 |
| -1860 | 2242.71 | 2142.14999999999 |
| -1820 | 2985.91 | 2814.27999999999 |
| -1780 | 2793.08 | 2688.25999999999 |
| -1740 | 2952.29 | 2640.85999999999 |
| -1700 | 2752.1 | 2620.83999999999 |
| -1660 | 2216.34 | 2120.67999999999 |
| -1620 | 2824.3 | 2767.24999999999 |
| -1580 | 2911.85 | 2656.02 |
| -1540 | 3011.40999999999 | 2955.08999999999 |
| -1500 | 2407.53 | 2362.56999999999 |
| -1460 | 2695.68 | 2601.96 |
| -1420 | 3160.91 | 3092.21999999999 |
| -1380 | 2888.44 | 2815.10999999999 |
| -1340 | 2742.37 | 2737.99999999999 |
| -1300 | 3168.0 | 3143.36999999999 |
| -1260 | 3163.35 | 3035.05999999999 |
| -1220 | 2375.52 | 2345.51999999999 |
| -1180 | 3043.36 | 2963.18999999999 |
| -1140 | 2922.48 | 2807.3 |
| -1100 | 2687.89 | 2620.81999999999 |
| -1060 | 2911.88 | 2713.14999999999 |
| -1020 | 2738.08 | 2787.68999999999 |
| -980 | 2808.21 | 2676.14 |
| -940 | 3057.83 | 3046.93 |
| -900 | 2945.27999999999 | 2810.21 |
| -860 | 2740.8 | 2530.42999999999 |
| -820 | 3109.05 | 3071.05999999999 |
| -780 | 2898.21 | 2949.71999999999 |
| -740 | 3564.07 | 3241.81999999999 |
| -700 | 3557.1 | 3413.10999999999 |
| -660 | 3584.39 | 3394.43999999999 |
| -620 | 3315.55 | 3193.33999999999 |
| -580 | 4577.72 | 4603.10999999999 |
| -540 | 3562.24 | 3422.71999999999 |
| -500 | 3236.64 | 3048.40999999999 |
| -460 | 3478.15999999999 | 3230.31999999999 |
| -420 | 3621.92 | 3560.05999999999 |
| -380 | 3401.05 | 3281.86999999999 |
| -340 | 3855.26 | 3835.74999999999 |
| -300 | 3437.42999999999 | 3331.04999999999 |
| -260 | 3929.83999999999 | 3948.66999999999 |
| -220 | 3144.71 | 3168.11999999999 |
| -180 | 2856.89 | 2703.42 |
| -140 | 2301.41999999999 | 2368.49999999999 |
| -100 | 2188.61 | 2118.48 |
| -60 | 2525.86 | 2460.73999999999 |
| -20 | 2599.51999999999 | 2423.68999999999 |
| 20 | 2178.41 | 2109.93 |
| 60 | 1294.95 | 1385.25 |
| 100 | 1459.06 | 1430.89 |
| 140 | 1230.4 | 1132.88 |
| 180 | 1466.82 | 1370.76 |
| 220 | 1371.71 | 1411.46 |
| 260 | 1476.62 | 1523.45 |
| 300 | 1424.72 | 1477.05 |
| 340 | 1306.58 | 1263.71 |
| 380 | 1572.7 | 1602.71 |
| 420 | 1504.63 | 1497.2 |
| 460 | 1158.99 | 1103.98 |
| 500 | 1247.32 | 1213.32 |
| 540 | 1170.18 | 1099.93 |
| 580 | 1604.59 | 1563.46 |
| 620 | 1776.64 | 1809.85 |
| 660 | 1489.56 | 1487.12 |
| 700 | 1579.99 | 1486.33 |
| 740 | 1379.85 | 1359.27 |
| 780 | 1647.31 | 1607.18 |
| 820 | 1306.89 | 1216.18 |
| 860 | 1422.63 | 1400.06 |
| 900 | 1257.77 | 1201.87 |
| 940 | 1575.21 | 1516.75 |
| 980 | 1449.59 | 1448.95 |
| 1020 | 1349.35 | 1240.31 |
| 1060 | 1974.44 | 1952.87 |
| 1100 | 1753.61 | 1642.38 |
| 1140 | 1864.81 | 1937.72 |
| 1180 | 1670.88 | 1670.91 |
| 1220 | 1481.89 | 1519.76 |
| 1260 | 1587.42 | 1572.42 |
| 1300 | 1809.36 | 1917.34 |
| 1340 | 1378.09 | 1444.13 |
| 1380 | 1897.37 | 1890.66 |
| 1420 | 1470.37 | 1586.84 |
| 1460 | 1501.13 | 1593.84 |
| 1500 | 1591.67 | 1534.94 |
| 1540 | 1655.37 | 1640.13 |
| 1580 | 1175.81 | 1105.46 |
| 1620 | 1692.96 | 1528.27 |
| 1660 | 1746.95 | 1773.2 |
| 1700 | 1665.58 | 1631.66 |
| 1740 | 1923.35 | 1967.03 |
| 1780 | 1784.06 | 1769.78 |
| 1820 | 1658.19 | 1456.4 |
| 1860 | 1495.12 | 1418.98 |
| 1900 | 1694.67 | 1686.12 |
| 1940 | 3534.78 | 3045.17 |
| 1980 | 2037.95 | 1895.49 |
### Chart: CCGG-TSS
| Category | E20CK | E20SD |
|---|---|---|
| -1980 | 690.96 | 675.05 |
| -1940 | 1885.76 | 2073.04 |
| -1900 | 1527.16 | 1547.51 |
| -1860 | 1510.63 | 1489.39 |
| -1820 | 1882.57 | 1846.44 |
| -1780 | 1694.02 | 1770.19 |
| -1740 | 1727.36 | 1749.82 |
| -1700 | 1818.08 | 2031.04 |
| -1660 | 1700.36 | 1514.58 |
| -1620 | 1362.67 | 1449.77 |
| -1580 | 1283.42 | 1302.23 |
| -1540 | 3883.93 | 1947.27999999999 |
| -1500 | 1514.85 | 1511.23 |
| -1460 | 1670.83 | 1681.29 |
| -1420 | 1589.07 | 1424.85 |
| -1380 | 1432.05 | 1381.09 |
| -1340 | 1580.69 | 1560.13 |
| -1300 | 1368.88 | 1354.47 |
| -1260 | 1618.49 | 1528.92 |
| -1220 | 1773.61 | 1596.88 |
| -1180 | 1546.44 | 1414.88 |
| -1140 | 1379.86 | 1412.34 |
| -1100 | 1549.94 | 1531.62 |
| -1060 | 1728.38 | 1799.47 |
| -1020 | 1851.76 | 1790.24 |
| -980 | 1346.52 | 1336.04 |
| -940 | 1738.11 | 1780.11 |
| -900 | 1166.04 | 1140.7 |
| -860 | 1339.85 | 1276.29 |
| -820 | 1571.69 | 1418.25 |
| -780 | 1281.72 | 1178.51 |
| -740 | 1448.18 | 1409.71 |
| -700 | 1567.41 | 1536.38 |
| -660 | 1788.97 | 1686.45 |
| -620 | 1552.69 | 1490.42 |
| -580 | 1384.33 | 1267.04 |
| -540 | 1454.12 | 1427.11 |
| -500 | 1538.97 | 1440.4 |
| -460 | 1012.04 | 1066.93 |
| -420 | 1376.01 | 1309.27 |
| -380 | 1354.97 | 1306.3 |
| -340 | 1498.45 | 1474.17 |
| -300 | 1133.72 | 1027.67 |
| -260 | 1527.41 | 1552.71 |
| -220 | 1472.76 | 1659.43 |
| -180 | 1220.31 | 1200.04 |
| -140 | 1468.57 | 1333.42 |
| -100 | 1090.2 | 1115.15 |
| -60 | 1063.92 | 968.380000000001 |
| -20 | 1232.27 | 1122.6 |
| 20 | 1571.56 | 1424.11 |
| 60 | 2977.22 | 2959.09999999999 |
| 100 | 2443.49 | 2467.48999999999 |
| 140 | 3055.22 | 2977.64999999999 |
| 180 | 2584.03 | 2571.48999999999 |
| 220 | 3035.68 | 2908.71999999998 |
| 260 | 2854.0 | 2947.65999999999 |
| 300 | 3269.9 | 3236.61999999999 |
| 340 | 5112.48 | 4642.05999999999 |
| 380 | 3190.38 | 3025.89999999999 |
| 420 | 2656.47 | 2559.33999999999 |
| 460 | 2634.92 | 2541.93999999999 |
| 500 | 2600.21 | 2506.74999999999 |
| 540 | 2137.08 | 2061.68999999999 |
| 580 | 2296.63 | 2270.36999999999 |
| 620 | 2271.4 | 2095.49 |
| 660 | 2564.49 | 2493.36999999999 |
| 700 | 2219.09 | 2088.9 |
| 740 | 2346.76 | 2351.1 |
| 780 | 2571.86 | 2588.99999999999 |
| 820 | 2797.54999999999 | 2628.51999999999 |
| 860 | 2475.41 | 2498.17 |
| 900 | 2570.17 | 2604.15999999999 |
| 940 | 2593.54 | 2531.87999999999 |
| 980 | 3342.15 | 3252.62999999999 |
| 1020 | 3266.71 | 3276.67999999999 |
| 1060 | 3301.83 | 3299.59999999999 |
| 1100 | 3235.81 | 3293.66999999999 |
| 1140 | 3049.99 | 2906.16 |
| 1180 | 2959.28 | 2877.66 |
| 1220 | 2877.92 | 2840.6 |
| 1260 | 3332.77 | 3205.45999999999 |
| 1300 | 3108.35 | 3080.62999999999 |
| 1340 | 3415.12 | 3255.92999999999 |
| 1380 | 3265.56999999999 | 3066.55999999999 |
| 1420 | 3273.75 | 3051.74999999999 |
| 1460 | 3244.58 | 3094.38 |
| 1500 | 3434.0 | 3284.48 |
| 1540 | 3225.13 | 2953.94999999999 |
| 1580 | 3153.62 | 2932.08999999999 |
| 1620 | 3227.92 | 3131.93999999999 |
| 1660 | 2551.18 | 2475.72999999999 |
| 1700 | 3248.62 | 3344.16 |
| 1740 | 3198.02 | 2991.32999999999 |
| 1780 | 3059.24 | 2845.00999999999 |
| 1820 | 2879.43 | 2927.65999999999 |
| 1860 | 3632.48 | 3286.35999999999 |
| 1900 | 3064.14 | 2922.84 |
| 1940 | 2866.13 | 2857.66999999999 |
| 1980 | 3429.83 | 3301.19999999999 |
### Chart: CCWGG-gene
| Category | E20CK | E20SD |
|---|---|---|
| 0.01 | 907.870000000001 | 842.509999999999 |
| 0.02 | 1544.09 | 1642.71 |
| 0.03 | 1075.54 | 1100.82 |
| 0.04 | 1041.92 | 1034.77 |
| 0.05 | 901.970000000001 | 853.08 |
| 0.06 | 1115.36 | 1182.39 |
| 7.0000000000000007E-2 | 1345.9 | 1450.87 |
| 0.08 | 1302.31 | 1332.89 |
| 0.09 | 1031.59 | 1086.28 |
| 0.1 | 869.210000000001 | 863.19 |
| 0.11 | 1040.51 | 1121.58 |
| 0.12 | 760.080000000001 | 804.87 |
| 0.13 | 879.910000000001 | 892.759999999999 |
| 0.14000000000000001 | 748.12 | 783.71 |
| 0.15 | 1017.43 | 1008.8 |
| 0.16 | 1069.14 | 1031.53 |
| 0.17 | 918.650000000001 | 943.92 |
| 0.18 | 887.760000000001 | 874.549999999999 |
| 0.19 | 1336.55 | 1325.1 |
| 0.2 | 845.320000000001 | 814.97 |
| 0.21 | 853.850000000001 | 801.59 |
| 0.22 | 732.800000000001 | 753.4 |
| 0.23 | 905.630000000001 | 920.759999999999 |
| 0.24 | 740.610000000001 | 712.86 |
| 0.25 | 826.270000000001 | 809.69 |
| 0.26 | 1006.12 | 990.609999999999 |
| 0.27 | 819.570000000001 | 822.649999999999 |
| 0.28000000000000003 | 873.920000000001 | 839.659999999999 |
| 0.28999999999999998 | 1101.53 | 1101.22 |
| 0.3 | 1164.9 | 1147.1 |
| 0.31 | 1164.5 | 1139.01 |
| 0.32 | 759.610000000001 | 771.89 |
| 0.33 | 783.890000000001 | 733.45 |
| 0.34 | 672.430000000001 | 669.31 |
| 0.35 | 906.320000000001 | 896.489999999999 |
| 0.36 | 924.640000000001 | 851.89 |
| 0.37 | 1386.46 | 1412.76 |
| 0.38 | 1095.56 | 1144.64 |
| 0.39 | 777.14 | 787.38 |
| 0.4 | 916.400000000001 | 878.64 |
| 0.41 | 929.370000000001 | 939.889999999999 |
| 0.42 | 900.820000000001 | 861.24 |
| 0.43 | 901.900000000001 | 879.45 |
| 0.44 | 944.360000000001 | 914.349999999999 |
| 0.45 | 1105.32 | 1034.1 |
| 0.46 | 1079.55 | 1062.87 |
| 0.47 | 960.060000000001 | 1059.88 |
| 0.48 | 1012.86 | 969.869999999999 |
| 0.49 | 919.850000000001 | 852.729999999999 |
| 0.5 | 994.690000000002 | 977.199999999999 |
| 0.51 | 785.650000000001 | 812.079999999999 |
| 0.52 | 1091.39 | 1110.69 |
| 0.53 | 956.310000000001 | 948.029999999999 |
| 0.54 | 1096.68 | 1117.58 |
| 0.55000000000000004 | 928.990000000001 | 907.409999999999 |
| 0.56000000000000005 | 945.850000000001 | 909.85 |
| 0.56999999999999995 | 821.710000000001 | 845.41 |
| 0.57999999999999996 | 1320.17 | 1510.95 |
| 0.59 | 957.760000000001 | 960.159999999999 |
| 0.6 | 955.090000000001 | 1027.14 |
| 0.61 | 963.990000000001 | 962.209999999999 |
| 0.62 | 981.250000000001 | 902.239999999999 |
| 0.63 | 908.200000000001 | 922.91 |
| 0.64 | 1012.9 | 1051.13 |
| 0.65 | 1138.77 | 1101.34 |
| 0.66 | 1095.19 | 1085.08 |
| 0.67 | 1043.76 | 1001.89 |
| 0.68 | 779.650000000001 | 756.169999999999 |
| 0.69 | 1007.69 | 1141.46 |
| 0.7 | 874.280000000001 | 840.54 |
| 0.71 | 1165.57 | 1236.33 |
| 0.72 | 865.720000000001 | 884.309999999999 |
| 0.73 | 966.310000000001 | 895.679999999999 |
| 0.74 | 1009.91 | 958.13 |
| 0.75 | 850.730000000001 | 826.33 |
| 0.76 | 892.560000000001 | 898.569999999999 |
| 0.77 | 1158.9 | 1156.08 |
| 0.78 | 1144.0 | 1141.39 |
| 0.79 | 968.230000000001 | 973.079999999999 |
| 0.8 | 1311.58 | 1272.43 |
| 0.81 | 1099.66 | 1026.71 |
| 0.82 | 959.650000000001 | 980.839999999999 |
| 0.83 | 942.870000000001 | 882.26 |
| 0.84 | 966.320000000001 | 906.619999999999 |
| 0.85 | 1054.23 | 1049.84 |
| 0.86 | 1078.74 | 1027.89 |
| 0.87 | 1035.48 | 1128.5 |
| 0.88 | 1232.19 | 1136.28 |
| 0.89 | 1872.55 | 1810.23 |
| 0.9 | 1051.9 | 1018.58 |
| 0.91 | 1250.09 | 1230.29 |
| 0.92 | 909.280000000002 | 885.439999999999 |
| 0.93 | 882.910000000001 | 876.62 |
| 0.94 | 1416.68 | 1452.89 |
| 0.95 | 1274.03 | 1269.24 |
| 0.96 | 1254.97 | 1186.13 |
| 0.97 | 1216.9 | 1255.36 |
| 0.98 | 1364.19 | 1323.21 |
| 0.99 | 1073.97 | 1130.9 |
| 1 | 1251.33 | 1303.62 |
### Chart: CCWGG-TTS
| Category | E20CK | E20SD |
|---|---|---|
| -1980 | 642.75 | 699.68 |
| -1940 | 1027.39 | 1017.83 |
| -1900 | 1361.53 | 1335.33 |
| -1860 | 1221.02 | 1154.92 |
| -1820 | 1240.8 | 1215.33 |
| -1780 | 1264.59 | 1240.44 |
| -1740 | 1815.57 | 1869.11 |
| -1700 | 1527.91 | 1504.5 |
| -1660 | 1386.0 | 1391.28 |
| -1620 | 1485.15 | 1369.03 |
| -1580 | 1326.46 | 1358.84 |
| -1540 | 2025.23 | 2118.95 |
| -1500 | 1605.48 | 1787.94 |
| -1460 | 1477.36 | 1363.64 |
| -1420 | 1456.44 | 1497.69 |
| -1380 | 1847.51 | 1833.79 |
| -1340 | 1370.79 | 1352.81 |
| -1300 | 1500.01 | 1533.77 |
| -1260 | 1574.83 | 1569.7 |
| -1220 | 1290.27 | 1341.1 |
| -1180 | 1336.1 | 1368.63 |
| -1140 | 1451.99 | 1427.44 |
| -1100 | 1362.89 | 1305.74 |
| -1060 | 1203.25 | 1267.68 |
| -1020 | 1274.61 | 1253.34 |
| -980 | 1137.93 | 1152.05 |
| -940 | 1379.69 | 1258.75 |
| -900 | 1532.05 | 1637.98 |
| -860 | 1655.65 | 1575.88 |
| -820 | 1413.62 | 1498.83 |
| -780 | 1472.44 | 1412.88 |
| -740 | 1935.11 | 2155.85 |
| -700 | 1640.0 | 1561.62 |
| -660 | 1826.18999999999 | 1818.45 |
| -620 | 1706.71 | 1655.42 |
| -580 | 1987.18 | 1957.13 |
| -540 | 1952.44999999999 | 1800.24 |
| -500 | 2622.93999999999 | 2593.98 |
| -460 | 1752.11 | 1712.2 |
| -420 | 1929.72999999999 | 2007.88 |
| -380 | 1901.48 | 2009.58 |
| -340 | 2062.44 | 1995.35 |
| -300 | 2165.86999999999 | 2177.4 |
| -260 | 2511.60999999999 | 2690.7 |
| -220 | 2525.75999999999 | 2507.52 |
| -180 | 2194.96 | 2154.22 |
| -140 | 1980.81 | 1980.55 |
| -100 | 1966.65 | 1969.28 |
| -60 | 2492.21999999999 | 2648.21 |
| -20 | 2203.58 | 2163.88 |
| 20 | 1904.13 | 1942.83 |
| 60 | 1415.19 | 1591.21 |
| 100 | 1117.83 | 1175.16 |
| 140 | 1063.97 | 1050.55 |
| 180 | 1241.27 | 1297.59 |
| 220 | 1217.04 | 1207.15 |
| 260 | 1073.6 | 1063.94 |
| 300 | 1195.41 | 1209.98 |
| 340 | 1215.57 | 1282.15 |
| 380 | 1140.29 | 1121.06 |
| 420 | 1329.14 | 1340.15 |
| 460 | 1034.05 | 1089.12 |
| 500 | 1221.06 | 1255.05 |
| 540 | 1567.81 | 1585.57 |
| 580 | 1335.42 | 1339.38 |
| 620 | 998.030000000001 | 945.479999999999 |
| 660 | 774.110000000001 | 736.649999999999 |
| 700 | 1154.15 | 1152.73 |
| 740 | 1053.86 | 1045.23 |
| 780 | 1305.34 | 1324.26 |
| 820 | 1247.16 | 1266.27 |
| 860 | 1298.23 | 1283.37 |
| 900 | 1646.51 | 1627.78 |
| 940 | 1412.55 | 1412.8 |
| 980 | 1345.57 | 1293.14 |
| 1020 | 1547.36 | 1478.13 |
| 1060 | 1180.52 | 1070.42 |
| 1100 | 1456.89 | 1446.5 |
| 1140 | 1176.13 | 1179.95 |
| 1180 | 1379.75 | 1458.63 |
| 1220 | 1085.93 | 1081.82 |
| 1260 | 1341.06 | 1326.8 |
| 1300 | 1462.48 | 1409.06 |
| 1340 | 1092.96 | 1098.83 |
| 1380 | 1243.4 | 1242.06 |
| 1420 | 1316.86 | 1347.79 |
| 1460 | 1163.74 | 1220.11 |
| 1500 | 1271.81 | 1270.83 |
| 1540 | 989.050000000001 | 951.15 |
| 1580 | 1145.54 | 1149.56 |
| 1620 | 990.580000000001 | 988.059999999999 |
| 1660 | 1279.92 | 1343.81 |
| 1700 | 1314.65 | 1257.38 |
| 1740 | 1493.82 | 1579.39 |
| 1780 | 1547.76 | 1515.46 |
| 1820 | 1346.91 | 1339.31 |
| 1860 | 1132.07 | 1202.29 |
| 1900 | 1322.81 | 1316.27 |
| 1940 | 1089.94 | 1084.26 |
| 1980 | 1209.55 | 1237.51 |
### Chart: CCWGG-TSS
| Category | E20CK | E20SD |
|---|---|---|
| -1980 | 719.52 | 715.53 |
| -1940 | 1276.97 | 1209.15 |
| -1900 | 1256.9 | 1145.91 |
| -1860 | 1257.25 | 1240.0 |
| -1820 | 1294.53 | 1279.37 |
| -1780 | 1695.61 | 1768.47 |
| -1740 | 1231.9 | 1299.19 |
| -1700 | 1234.87 | 1240.76 |
| -1660 | 1251.22 | 1255.4 |
| -1620 | 1285.14 | 1396.95 |
| -1580 | 1319.41 | 1172.71 |
| -1540 | 1716.88 | 1671.14 |
| -1500 | 1180.54 | 1209.94 |
| -1460 | 1205.42 | 1285.0 |
| -1420 | 990.650000000001 | 857.93 |
| -1380 | 1427.1 | 1292.31 |
| -1340 | 1132.11 | 1162.52 |
| -1300 | 1114.91 | 1102.52 |
| -1260 | 1408.41 | 1425.71 |
| -1220 | 1156.66 | 1160.1 |
| -1180 | 1250.21 | 1266.78 |
| -1140 | 1355.53 | 1232.8 |
| -1100 | 1026.36 | 1041.6 |
| -1060 | 1329.54 | 1345.81 |
| -1020 | 1472.57 | 1458.21 |
| -980 | 1086.3 | 1111.0 |
| -940 | 1448.75 | 1489.8 |
| -900 | 1185.68 | 1057.83 |
| -860 | 1273.73 | 1426.14 |
| -820 | 1017.79 | 973.549999999999 |
| -780 | 1423.73 | 1407.91 |
| -740 | 1328.75 | 1351.94 |
| -700 | 1382.04 | 1401.0 |
| -660 | 1355.92 | 1349.83 |
| -620 | 1210.31 | 1282.55 |
| -580 | 1133.97 | 1141.45 |
| -540 | 1130.61 | 1154.05 |
| -500 | 1493.37 | 1469.11 |
| -460 | 1273.96 | 1288.68 |
| -420 | 1357.02 | 1300.41 |
| -380 | 1257.22 | 1299.6 |
| -340 | 1060.18 | 1126.84 |
| -300 | 1711.36 | 1828.07 |
| -260 | 1230.44 | 1286.17 |
| -220 | 1570.15 | 1657.69 |
| -180 | 1154.73 | 1132.11 |
| -140 | 1620.03 | 1512.51 |
| -100 | 1367.5 | 1312.97 |
| -60 | 1304.17 | 1414.42 |
| -20 | 1197.68 | 1171.82 |
| 20 | 1681.75 | 1578.7 |
| 60 | 2571.22999999999 | 2597.17 |
| 100 | 2524.61999999999 | 2649.4 |
| 140 | 2203.23999999999 | 2410.49 |
| 180 | 2146.53999999999 | 2174.52 |
| 220 | 1805.85 | 1855.7 |
| 260 | 1810.96 | 1775.93 |
| 300 | 1674.3 | 1711.82 |
| 340 | 1787.99 | 1915.4 |
| 380 | 2216.27 | 2267.03 |
| 420 | 1941.28 | 1826.51 |
| 460 | 1569.93 | 1569.73 |
| 500 | 1646.03 | 1633.07 |
| 540 | 1415.84 | 1418.07 |
| 580 | 1520.93 | 1552.73 |
| 620 | 1633.38 | 1663.01 |
| 660 | 1546.94 | 1639.89 |
| 700 | 1481.34 | 1365.04 |
| 740 | 1732.44 | 1738.1 |
| 780 | 1309.26 | 1386.12 |
| 820 | 1630.77 | 1699.58 |
| 860 | 1525.68 | 1515.9 |
| 900 | 1559.56 | 1578.34 |
| 940 | 1472.74 | 1633.83 |
| 980 | 1528.66 | 1465.89 |
| 1020 | 1251.3 | 1252.17 |
| 1060 | 1463.15 | 1492.33 |
| 1100 | 1458.71 | 1371.43 |
| 1140 | 1346.93 | 1292.38 |
| 1180 | 1771.15 | 1725.13 |
| 1220 | 1641.81 | 1585.24 |
| 1260 | 1224.4 | 1172.68 |
| 1300 | 1842.65 | 1863.46 |
| 1340 | 1465.06 | 1505.34 |
| 1380 | 1496.29 | 1476.48 |
| 1420 | 1298.51 | 1279.87 |
| 1460 | 1241.42 | 1284.23 |
| 1500 | 1254.24 | 1331.71 |
| 1540 | 1258.96 | 1223.54 |
| 1580 | 1292.2 | 1317.93 |
| 1620 | 1412.46 | 1335.4 |
| 1660 | 1351.43 | 1307.37 |
| 1700 | 1387.49 | 1351.24 |
| 1740 | 1251.93 | 1182.84 |
| 1780 | 1595.08 | 1515.77 |
| 1820 | 1488.38 | 1442.03 |
| 1860 | 1325.31 | 1448.52 |
| 1900 | 1658.63 | 1785.11 |
| 1940 | 1037.76 | 1103.36 |
| 1980 | 1886.98 | 1870.33 |Supplementary Figure S1. DNA methylation levels at the CCGG and CCWGG sites in different regions of genes in E20. X-axis showed the ±2000 bp of transcription start site (TSS), the relative position of gene body and the ±2000 bp of transcription termination site (TTS), respectively. Y-axis showed the relative DNA methylation level.

## Slide 3
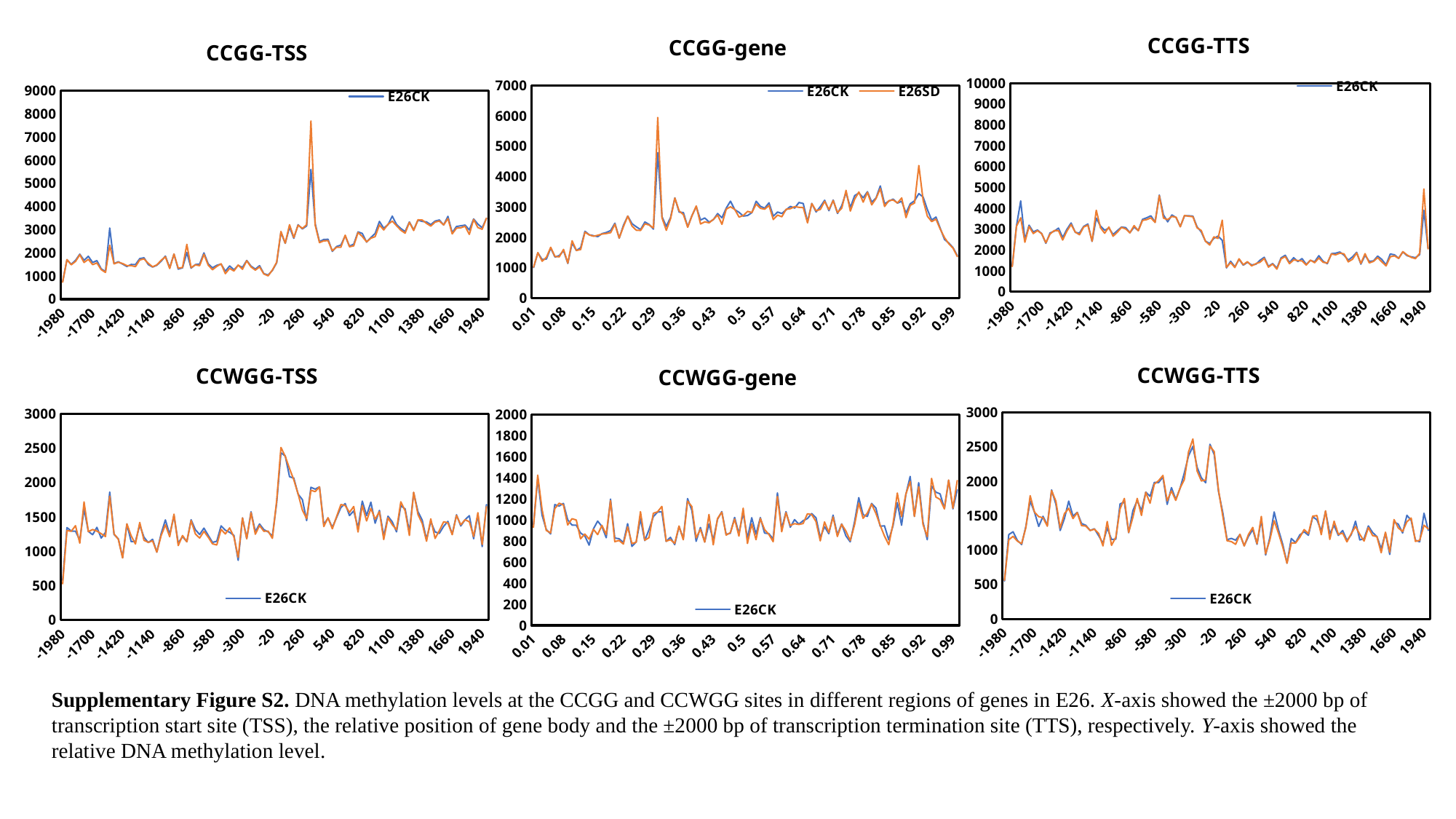

### Chart: CCGG-TTS
| Category | E26CK | E26SD |
|---|---|---|
| -1980 | 1250.68 | 1202.59 |
| -1940 | 3150.40999999999 | 3133.23 |
| -1900 | 4353.78999999999 | 3543.11 |
| -1860 | 2489.30999999999 | 2371.78 |
| -1820 | 3187.66999999999 | 3127.83 |
| -1780 | 2866.41999999999 | 2787.93 |
| -1740 | 2958.47999999999 | 2926.37 |
| -1700 | 2785.03999999999 | 2782.76 |
| -1660 | 2320.61 | 2349.77 |
| -1620 | 2816.52999999999 | 2768.44 |
| -1580 | 2899.14999999999 | 2930.99000000001 |
| -1540 | 3048.24999999999 | 2926.75 |
| -1500 | 2595.82999999999 | 2476.79 |
| -1460 | 2987.42 | 2904.88000000001 |
| -1420 | 3296.71999999999 | 3222.29 |
| -1380 | 2844.32999999999 | 2857.13 |
| -1340 | 2811.75999999999 | 2730.52 |
| -1300 | 3131.57 | 3102.85 |
| -1260 | 3245.47999999999 | 3190.53000000001 |
| -1220 | 2410.61 | 2417.28 |
| -1180 | 3526.18999999999 | 3903.57000000001 |
| -1140 | 3146.26 | 3063.16000000001 |
| -1100 | 2947.55999999999 | 2803.52 |
| -1060 | 3049.71999999999 | 3099.91 |
| -1020 | 2742.53999999999 | 2658.3 |
| -980 | 2924.78 | 2849.1 |
| -940 | 3097.57999999999 | 3081.72 |
| -900 | 3072.46 | 3012.35 |
| -860 | 2827.60999999999 | 2820.82 |
| -820 | 3098.90999999999 | 3169.03 |
| -780 | 2934.84999999999 | 2922.94 |
| -740 | 3468.37999999999 | 3416.9 |
| -700 | 3542.78999999999 | 3455.70000000001 |
| -660 | 3638.65 | 3540.94 |
| -620 | 3332.94999999999 | 3321.51000000001 |
| -580 | 4633.36999999999 | 4608.10000000001 |
| -540 | 3684.90999999998 | 3526.91 |
| -500 | 3349.40999999999 | 3457.70000000001 |
| -460 | 3680.34999999999 | 3609.11000000001 |
| -420 | 3553.66999999999 | 3569.08000000001 |
| -380 | 3121.88999999999 | 3110.50000000001 |
| -340 | 3639.02999999999 | 3654.93000000001 |
| -300 | 3640.82999999999 | 3616.85000000001 |
| -260 | 3622.83999999999 | 3595.72000000001 |
| -220 | 3107.57999999999 | 3060.99000000001 |
| -180 | 2867.04999999999 | 2938.03000000001 |
| -140 | 2422.34999999999 | 2420.44 |
| -100 | 2313.64 | 2238.32 |
| -60 | 2558.96999999999 | 2629.08 |
| -20 | 2666.75999999999 | 2553.52 |
| 20 | 2465.55 | 3429.09 |
| 60 | 1142.09 | 1167.02 |
| 100 | 1458.27 | 1395.85 |
| 140 | 1193.61 | 1158.08 |
| 180 | 1554.95 | 1579.04 |
| 220 | 1271.85 | 1293.23 |
| 260 | 1410.64 | 1440.11 |
| 300 | 1283.7 | 1235.38 |
| 340 | 1313.63 | 1343.06 |
| 380 | 1510.95 | 1413.52 |
| 420 | 1648.6 | 1593.33 |
| 460 | 1213.35 | 1178.89 |
| 500 | 1350.3 | 1325.27 |
| 540 | 1131.83 | 1082.19 |
| 580 | 1622.52 | 1576.06 |
| 620 | 1744.45 | 1682.8 |
| 660 | 1393.06 | 1344.3 |
| 700 | 1630.67 | 1523.75 |
| 740 | 1444.41 | 1492.05 |
| 780 | 1588.45 | 1475.48 |
| 820 | 1305.97 | 1276.75 |
| 860 | 1497.67 | 1513.92 |
| 900 | 1433.68 | 1389.55 |
| 940 | 1724.36 | 1616.16 |
| 980 | 1460.05 | 1411.1 |
| 1020 | 1341.45 | 1375.14 |
| 1060 | 1822.81 | 1806.12 |
| 1100 | 1846.22 | 1767.31 |
| 1140 | 1901.86 | 1848.29 |
| 1180 | 1749.54 | 1813.33 |
| 1220 | 1513.08 | 1429.63 |
| 1260 | 1667.96 | 1554.51 |
| 1300 | 1887.87 | 1857.71 |
| 1340 | 1321.35 | 1353.26 |
| 1380 | 1744.48 | 1823.02 |
| 1420 | 1455.37 | 1380.52 |
| 1460 | 1476.61 | 1452.81 |
| 1500 | 1706.33 | 1634.69 |
| 1540 | 1546.04 | 1429.98 |
| 1580 | 1281.8 | 1235.9 |
| 1620 | 1808.77 | 1674.43 |
| 1660 | 1767.58 | 1717.89 |
| 1700 | 1603.25 | 1596.76 |
| 1740 | 1907.73 | 1918.77 |
| 1780 | 1726.87 | 1759.67 |
| 1820 | 1672.05 | 1643.63 |
| 1860 | 1632.11 | 1583.59 |
| 1900 | 1776.27 | 1842.8 |
| 1940 | 3904.34999999999 | 4926.87 |
| 1980 | 2068.41 | 2053.45 |
### Chart: CCGG-gene
| Category | E26CK | E26SD |
|---|---|---|
| 0.01 | 1004.33 | 1019.23 |
| 0.02 | 1490.5 | 1499.59 |
| 0.03 | 1272.44 | 1215.49 |
| 0.04 | 1287.4 | 1344.4 |
| 0.05 | 1648.25 | 1673.63 |
| 0.06 | 1354.61 | 1370.47 |
| 7.0000000000000007E-2 | 1402.29 | 1359.99 |
| 0.08 | 1558.51 | 1604.35 |
| 0.09 | 1142.03 | 1154.72 |
| 0.1 | 1816.96 | 1886.75 |
| 0.11 | 1567.0 | 1570.61 |
| 0.12 | 1661.48 | 1600.14 |
| 0.13 | 2200.84 | 2169.5 |
| 0.14000000000000001 | 2084.12 | 2080.92 |
| 0.15 | 2055.21 | 2036.61 |
| 0.16 | 2024.81 | 2069.49 |
| 0.17 | 2122.89 | 2109.2 |
| 0.18 | 2161.4 | 2127.8 |
| 0.19 | 2224.28 | 2154.35 |
| 0.2 | 2467.32999999999 | 2432.98 |
| 0.21 | 1971.44 | 1978.78 |
| 0.22 | 2402.9 | 2358.61 |
| 0.23 | 2699.66 | 2698.89 |
| 0.24 | 2448.01 | 2368.82 |
| 0.25 | 2346.95 | 2234.95 |
| 0.26 | 2255.76 | 2228.26 |
| 0.27 | 2506.04 | 2444.73 |
| 0.28000000000000003 | 2424.86 | 2406.79 |
| 0.28999999999999998 | 2271.9 | 2312.18 |
| 0.3 | 4788.21999999999 | 5945.31000000001 |
| 0.31 | 2679.65999999999 | 2640.18 |
| 0.32 | 2361.58 | 2231.53 |
| 0.33 | 2655.03 | 2611.46 |
| 0.34 | 3298.85 | 3293.65 |
| 0.35 | 2830.75999999999 | 2868.13000000001 |
| 0.36 | 2815.04 | 2748.15 |
| 0.37 | 2349.89 | 2332.84 |
| 0.38 | 2718.87 | 2712.35 |
| 0.39 | 3008.67999999999 | 3034.05 |
| 0.4 | 2569.84999999999 | 2441.79 |
| 0.41 | 2638.32 | 2510.55 |
| 0.42 | 2500.18 | 2477.73 |
| 0.43 | 2584.1 | 2592.45 |
| 0.44 | 2783.56 | 2731.01 |
| 0.45 | 2644.13 | 2428.32 |
| 0.46 | 2961.83999999999 | 2928.59 |
| 0.47 | 3191.30999999999 | 3005.71 |
| 0.48 | 2911.66 | 2919.66 |
| 0.49 | 2823.75999999999 | 2667.99 |
| 0.5 | 2700.49 | 2719.7 |
| 0.51 | 2718.85 | 2855.04 |
| 0.52 | 2813.92 | 2817.92 |
| 0.53 | 3188.76 | 3094.81 |
| 0.54 | 3020.99999999999 | 2956.35 |
| 0.55000000000000004 | 2958.48 | 2927.28 |
| 0.56000000000000005 | 3137.85 | 3048.65000000001 |
| 0.56999999999999995 | 2696.90999999999 | 2587.04 |
| 0.57999999999999996 | 2832.57 | 2733.51 |
| 0.59 | 2784.61999999999 | 2678.23 |
| 0.6 | 2905.49 | 2923.87 |
| 0.61 | 3017.87 | 2945.01 |
| 0.62 | 2963.4 | 3011.01 |
| 0.63 | 3147.72999999999 | 2988.83 |
| 0.64 | 3113.30999999999 | 2979.14 |
| 0.65 | 2511.63999999999 | 2474.84 |
| 0.66 | 3114.42999999999 | 3111.72 |
| 0.67 | 2835.51 | 2874.47 |
| 0.68 | 3012.29999999999 | 2920.63 |
| 0.69 | 3220.61 | 3183.85 |
| 0.7 | 2877.60999999999 | 2914.47 |
| 0.71 | 3228.33 | 3212.21 |
| 0.72 | 2786.48 | 2818.27 |
| 0.73 | 3046.66999999999 | 2961.31 |
| 0.74 | 3468.39 | 3546.09 |
| 0.75 | 2979.84999999999 | 2864.74 |
| 0.76 | 3373.50999999999 | 3255.62 |
| 0.77 | 3459.59999999999 | 3492.92000000001 |
| 0.78 | 3301.49999999999 | 3158.95 |
| 0.79 | 3501.72999999999 | 3481.47 |
| 0.8 | 3157.25999999999 | 3069.79 |
| 0.81 | 3297.16999999999 | 3280.15000000001 |
| 0.82 | 3692.06999999999 | 3602.94 |
| 0.83 | 3103.88 | 3016.21 |
| 0.84 | 3191.63999999999 | 3207.88 |
| 0.85 | 3258.24999999999 | 3229.94 |
| 0.86 | 3127.23 | 3131.22 |
| 0.87 | 3186.55999999999 | 3295.34 |
| 0.88 | 2797.47 | 2649.07 |
| 0.89 | 3106.35999999999 | 3054.75 |
| 0.9 | 3202.62999999999 | 3130.76 |
| 0.91 | 3437.97 | 4364.13 |
| 0.92 | 3326.39 | 3217.34000000001 |
| 0.93 | 2914.95 | 2696.7 |
| 0.94 | 2569.03999999999 | 2519.38 |
| 0.95 | 2667.99999999999 | 2597.97 |
| 0.96 | 2298.22 | 2270.04 |
| 0.97 | 1929.28 | 1990.16 |
| 0.98 | 1814.41 | 1790.09 |
| 0.99 | 1657.45 | 1641.89 |
| 1 | 1394.49 | 1364.99 |
### Chart: CCGG-TSS
| Category | E26CK | E26SD |
|---|---|---|
| -1980 | 755.110000000001 | 730.61 |
| -1940 | 1698.75 | 1705.62 |
| -1900 | 1503.96 | 1481.1 |
| -1860 | 1670.23 | 1625.01 |
| -1820 | 1939.51 | 1913.32 |
| -1780 | 1672.36 | 1581.96 |
| -1740 | 1849.52 | 1717.84 |
| -1700 | 1579.37 | 1486.59 |
| -1660 | 1661.38 | 1563.41 |
| -1620 | 1307.38 | 1272.56 |
| -1580 | 1188.55 | 1152.28 |
| -1540 | 3067.92 | 2323.1 |
| -1500 | 1532.98 | 1523.73 |
| -1460 | 1613.81 | 1591.7 |
| -1420 | 1511.0 | 1535.93 |
| -1380 | 1406.7 | 1444.36 |
| -1340 | 1495.94 | 1446.45 |
| -1300 | 1489.77 | 1402.09 |
| -1260 | 1747.36 | 1689.13 |
| -1220 | 1784.03 | 1736.07 |
| -1180 | 1491.62 | 1546.9 |
| -1140 | 1385.68 | 1395.35 |
| -1100 | 1460.83 | 1476.45 |
| -1060 | 1633.1 | 1674.36 |
| -1020 | 1856.11 | 1827.28 |
| -980 | 1353.2 | 1321.07 |
| -940 | 1940.69 | 1948.35 |
| -900 | 1295.8 | 1333.75 |
| -860 | 1350.73 | 1368.29 |
| -820 | 2014.25 | 2363.68 |
| -780 | 1334.5 | 1359.77 |
| -740 | 1491.62 | 1475.61 |
| -700 | 1515.24 | 1443.98 |
| -660 | 1995.44 | 1933.59 |
| -620 | 1521.64 | 1465.92 |
| -580 | 1350.67 | 1269.19 |
| -540 | 1458.58 | 1413.99 |
| -500 | 1511.33 | 1525.0 |
| -460 | 1203.95 | 1099.08 |
| -420 | 1431.5 | 1325.71 |
| -380 | 1266.88 | 1214.74 |
| -340 | 1455.97 | 1471.37 |
| -300 | 1368.66 | 1291.54 |
| -260 | 1668.39 | 1654.49 |
| -220 | 1422.76 | 1389.86 |
| -180 | 1296.05 | 1251.41 |
| -140 | 1442.94 | 1390.77 |
| -100 | 1099.0 | 1085.62 |
| -60 | 1040.7 | 1004.9 |
| -20 | 1239.38 | 1255.21 |
| 20 | 1569.21 | 1593.33 |
| 60 | 2883.17999999999 | 2920.78 |
| 100 | 2408.4 | 2412.51 |
| 140 | 3114.78999999999 | 3211.24000000001 |
| 180 | 2625.55999999999 | 2657.75000000001 |
| 220 | 3210.80999999999 | 3188.93000000001 |
| 260 | 3029.35999999999 | 3051.54 |
| 300 | 3159.51999999999 | 3213.74000000001 |
| 340 | 5601.59999999999 | 7691.63 |
| 380 | 3258.40999999999 | 3190.96 |
| 420 | 2480.62999999999 | 2436.62 |
| 460 | 2573.84999999999 | 2517.35 |
| 500 | 2581.61 | 2521.1 |
| 540 | 2060.01 | 2088.89 |
| 580 | 2272.99 | 2241.24 |
| 620 | 2332.33999999999 | 2240.72 |
| 660 | 2730.18999999999 | 2760.88 |
| 700 | 2288.01 | 2259.46 |
| 740 | 2373.2 | 2300.01 |
| 780 | 2902.84999999999 | 2873.22 |
| 820 | 2834.15999999999 | 2703.42000000001 |
| 860 | 2461.78999999999 | 2472.52 |
| 900 | 2659.85 | 2629.15 |
| 940 | 2833.37 | 2695.83 |
| 980 | 3351.40999999999 | 3200.25000000001 |
| 1020 | 3058.87999999999 | 2987.11000000001 |
| 1060 | 3211.49 | 3230.22 |
| 1100 | 3582.99999999999 | 3367.42 |
| 1140 | 3218.37999999999 | 3169.02 |
| 1180 | 3048.46999999999 | 2973.22 |
| 1220 | 2914.60999999999 | 2845.78 |
| 1260 | 3331.55999999999 | 3313.43 |
| 1300 | 2986.74 | 2961.37000000001 |
| 1340 | 3409.70999999999 | 3422.86 |
| 1380 | 3353.41999999999 | 3412.62000000001 |
| 1420 | 3331.95999999999 | 3268.18000000001 |
| 1460 | 3215.13999999999 | 3151.8 |
| 1500 | 3368.87999999999 | 3322.26000000001 |
| 1540 | 3415.34 | 3371.31000000001 |
| 1580 | 3201.97999999999 | 3202.01 |
| 1620 | 3570.57999999999 | 3476.79 |
| 1660 | 2861.54999999999 | 2816.61000000001 |
| 1700 | 3134.23 | 3063.22 |
| 1740 | 3164.19 | 3078.7 |
| 1780 | 3193.77999999999 | 3144.21 |
| 1820 | 2985.96999999999 | 2792.99 |
| 1860 | 3459.68999999999 | 3437.18000000001 |
| 1900 | 3238.93999999999 | 3088.79 |
| 1940 | 3080.09999999999 | 3014.46 |
| 1980 | 3488.53999999999 | 3495.30000000001 |
### Chart: CCWGG-TTS
| Category | E26CK | E26SD |
|---|---|---|
| -1980 | 548.95 | 560.26 |
| -1940 | 1224.38 | 1148.82 |
| -1900 | 1265.15 | 1198.94 |
| -1860 | 1140.28 | 1130.46 |
| -1820 | 1079.79 | 1092.64 |
| -1780 | 1344.15 | 1326.54 |
| -1740 | 1713.24 | 1790.46 |
| -1700 | 1550.97 | 1551.45 |
| -1660 | 1342.97 | 1484.65 |
| -1620 | 1490.49 | 1468.4 |
| -1580 | 1361.02 | 1346.64 |
| -1540 | 1873.91 | 1863.9 |
| -1500 | 1667.78 | 1712.0 |
| -1460 | 1283.66 | 1319.01 |
| -1420 | 1457.66 | 1527.59 |
| -1380 | 1712.01 | 1606.87 |
| -1340 | 1492.58 | 1457.11 |
| -1300 | 1548.02 | 1543.55 |
| -1260 | 1385.02 | 1354.87 |
| -1220 | 1358.88 | 1348.24 |
| -1180 | 1284.98 | 1283.18 |
| -1140 | 1308.43 | 1302.33 |
| -1100 | 1207.57 | 1242.3 |
| -1060 | 1098.7 | 1059.6 |
| -1020 | 1323.12 | 1414.95 |
| -980 | 1153.79 | 1068.7 |
| -940 | 1158.02 | 1185.99 |
| -900 | 1670.8 | 1599.37 |
| -860 | 1698.5 | 1750.4 |
| -820 | 1251.74 | 1256.07 |
| -780 | 1573.22 | 1477.5 |
| -740 | 1720.77 | 1751.2 |
| -700 | 1572.37 | 1503.45 |
| -660 | 1842.28 | 1839.61 |
| -620 | 1781.26 | 1680.77 |
| -580 | 1983.12 | 1959.02 |
| -540 | 1980.96 | 2009.14 |
| -500 | 2062.56 | 2083.76 |
| -460 | 1663.56 | 1715.77 |
| -420 | 1907.89 | 1860.48 |
| -380 | 1735.09 | 1722.01 |
| -340 | 1894.01 | 1900.58 |
| -300 | 2120.52 | 2024.45 |
| -260 | 2370.68 | 2428.55 |
| -220 | 2506.39 | 2614.64 |
| -180 | 2202.12 | 2147.9 |
| -140 | 2049.9 | 2004.89 |
| -100 | 1979.32 | 2019.01 |
| -60 | 2537.56 | 2511.7 |
| -20 | 2388.3 | 2431.22 |
| 20 | 1849.44 | 1870.01 |
| 60 | 1545.94 | 1494.31 |
| 100 | 1149.56 | 1135.1 |
| 140 | 1169.36 | 1122.12 |
| 180 | 1142.92 | 1082.93 |
| 220 | 1224.79 | 1229.37 |
| 260 | 1063.39 | 1059.13 |
| 300 | 1198.31 | 1226.53 |
| 340 | 1297.11 | 1328.61 |
| 380 | 1083.19 | 1092.18 |
| 420 | 1446.35 | 1488.4 |
| 460 | 926.78 | 944.399999999999 |
| 500 | 1182.75 | 1147.67 |
| 540 | 1553.54 | 1427.07 |
| 580 | 1295.47 | 1245.23 |
| 620 | 1083.13 | 1046.2 |
| 660 | 809.459999999999 | 807.609999999999 |
| 700 | 1167.74 | 1104.16 |
| 740 | 1107.5 | 1101.73 |
| 780 | 1220.2 | 1186.8 |
| 820 | 1266.04 | 1296.84 |
| 860 | 1213.87 | 1238.97 |
| 900 | 1483.75 | 1492.09 |
| 940 | 1439.66 | 1500.87 |
| 980 | 1276.97 | 1223.04 |
| 1020 | 1562.34 | 1569.76 |
| 1060 | 1238.34 | 1154.65 |
| 1100 | 1356.8 | 1419.15 |
| 1140 | 1212.68 | 1228.49 |
| 1180 | 1283.63 | 1245.2 |
| 1220 | 1139.11 | 1119.23 |
| 1260 | 1222.29 | 1238.55 |
| 1300 | 1418.98 | 1341.97 |
| 1340 | 1147.06 | 1227.68 |
| 1380 | 1163.91 | 1130.06 |
| 1420 | 1350.57 | 1326.55 |
| 1460 | 1254.69 | 1209.71 |
| 1500 | 1195.82 | 1195.52 |
| 1540 | 1019.26 | 961.159999999999 |
| 1580 | 1244.99 | 1259.0 |
| 1620 | 935.229999999999 | 969.049999999999 |
| 1660 | 1408.54 | 1442.17 |
| 1700 | 1375.71 | 1320.3 |
| 1740 | 1247.1 | 1269.83 |
| 1780 | 1503.92 | 1411.66 |
| 1820 | 1432.03 | 1464.99 |
| 1860 | 1142.42 | 1125.06 |
| 1900 | 1119.32 | 1141.72 |
| 1940 | 1533.76 | 1360.34 |
| 1980 | 1285.38 | 1307.32 |
### Chart: CCWGG-TSS
| Category | E26CK | E26SD |
|---|---|---|
| -1980 | 579.18 | 527.32 |
| -1940 | 1344.24 | 1300.3 |
| -1900 | 1293.82 | 1291.1 |
| -1860 | 1292.06 | 1371.58 |
| -1820 | 1161.75 | 1117.95 |
| -1780 | 1614.49 | 1716.97 |
| -1740 | 1289.1 | 1285.28 |
| -1700 | 1240.82 | 1315.71 |
| -1660 | 1347.59 | 1292.77 |
| -1620 | 1192.01 | 1255.66 |
| -1580 | 1274.06 | 1214.34 |
| -1540 | 1861.22 | 1801.71 |
| -1500 | 1244.58 | 1252.71 |
| -1460 | 1180.73 | 1173.84 |
| -1420 | 908.689999999999 | 903.089999999999 |
| -1380 | 1395.92 | 1398.33 |
| -1340 | 1141.16 | 1224.38 |
| -1300 | 1141.6 | 1105.84 |
| -1260 | 1381.63 | 1419.17 |
| -1220 | 1203.37 | 1159.27 |
| -1180 | 1126.03 | 1130.48 |
| -1140 | 1173.89 | 1151.7 |
| -1100 | 988.169999999999 | 986.95 |
| -1060 | 1255.13 | 1228.02 |
| -1020 | 1455.66 | 1384.56 |
| -980 | 1242.05 | 1211.48 |
| -940 | 1518.34 | 1539.68 |
| -900 | 1117.61 | 1082.06 |
| -860 | 1213.88 | 1228.93 |
| -820 | 1139.09 | 1137.97 |
| -780 | 1458.06 | 1445.88 |
| -740 | 1312.68 | 1251.39 |
| -700 | 1243.74 | 1192.17 |
| -660 | 1336.27 | 1288.99 |
| -620 | 1230.7 | 1203.92 |
| -580 | 1128.96 | 1108.34 |
| -540 | 1145.37 | 1091.66 |
| -500 | 1369.87 | 1318.58 |
| -460 | 1306.33 | 1251.42 |
| -420 | 1276.04 | 1339.1 |
| -380 | 1231.1 | 1208.91 |
| -340 | 868.36 | 911.9 |
| -300 | 1484.62 | 1483.79 |
| -260 | 1185.72 | 1180.97 |
| -220 | 1574.11 | 1561.0 |
| -180 | 1284.11 | 1248.12 |
| -140 | 1396.28 | 1374.89 |
| -100 | 1314.75 | 1290.67 |
| -60 | 1281.14 | 1292.71 |
| -20 | 1222.34 | 1187.67 |
| 20 | 1705.71 | 1737.83 |
| 60 | 2432.08 | 2509.66 |
| 100 | 2385.79 | 2381.13 |
| 140 | 2084.81 | 2203.0 |
| 180 | 2063.77 | 2039.95 |
| 220 | 1830.93 | 1835.43 |
| 260 | 1751.54 | 1598.59 |
| 300 | 1446.31 | 1474.69 |
| 340 | 1930.17 | 1887.23 |
| 380 | 1904.07 | 1868.09 |
| 420 | 1936.45 | 1937.23 |
| 460 | 1389.14 | 1359.15 |
| 500 | 1483.73 | 1487.2 |
| 540 | 1346.32 | 1326.58 |
| 580 | 1492.96 | 1505.57 |
| 620 | 1636.32 | 1679.94 |
| 660 | 1693.91 | 1665.65 |
| 700 | 1519.04 | 1565.14 |
| 740 | 1587.15 | 1651.58 |
| 780 | 1338.74 | 1280.28 |
| 820 | 1730.5 | 1666.59 |
| 860 | 1519.11 | 1441.21 |
| 900 | 1714.09 | 1623.18 |
| 940 | 1407.68 | 1466.32 |
| 980 | 1593.81 | 1574.82 |
| 1020 | 1216.8 | 1170.54 |
| 1060 | 1509.83 | 1486.72 |
| 1100 | 1427.77 | 1385.77 |
| 1140 | 1282.4 | 1313.62 |
| 1180 | 1663.59 | 1719.46 |
| 1220 | 1615.27 | 1588.57 |
| 1260 | 1284.05 | 1229.8 |
| 1300 | 1851.91 | 1857.55 |
| 1340 | 1576.2 | 1534.73 |
| 1380 | 1451.36 | 1402.89 |
| 1420 | 1171.45 | 1145.94 |
| 1460 | 1432.01 | 1470.94 |
| 1500 | 1280.65 | 1186.83 |
| 1540 | 1260.06 | 1301.12 |
| 1580 | 1363.07 | 1428.33 |
| 1620 | 1434.6 | 1413.35 |
| 1660 | 1248.74 | 1240.65 |
| 1700 | 1530.32 | 1521.78 |
| 1740 | 1367.73 | 1385.38 |
| 1780 | 1458.96 | 1457.96 |
| 1820 | 1517.8 | 1429.93 |
| 1860 | 1181.94 | 1214.71 |
| 1900 | 1526.63 | 1560.25 |
| 1940 | 1067.61 | 1098.86 |
| 1980 | 1679.66 | 1646.91 |
### Chart: CCWGG-gene
| Category | E26CK | E26SD |
|---|---|---|
| 0.01 | 954.16 | 929.009999999999 |
| 0.02 | 1395.54 | 1425.84 |
| 0.03 | 1045.79 | 1100.85 |
| 0.04 | 908.7 | 900.59 |
| 0.05 | 867.139999999999 | 878.07 |
| 0.06 | 1146.24 | 1107.95 |
| 7.0000000000000007E-2 | 1132.38 | 1159.26 |
| 0.08 | 1157.57 | 1143.01 |
| 0.09 | 1002.04 | 951.99 |
| 0.1 | 951.569999999999 | 1012.04 |
| 0.11 | 950.33 | 999.859999999999 |
| 0.12 | 876.35 | 821.389999999999 |
| 0.13 | 848.169999999999 | 866.45 |
| 0.14000000000000001 | 761.18 | 818.05 |
| 0.15 | 915.099999999999 | 911.1 |
| 0.16 | 989.02 | 861.429999999999 |
| 0.17 | 938.539999999999 | 948.669999999999 |
| 0.18 | 831.38 | 861.919999999999 |
| 0.19 | 1198.77 | 1183.91 |
| 0.2 | 828.429999999999 | 794.25 |
| 0.21 | 821.7 | 805.519999999999 |
| 0.22 | 785.529999999999 | 771.359999999999 |
| 0.23 | 965.069999999999 | 934.39 |
| 0.24 | 748.989999999999 | 775.97 |
| 0.25 | 791.43 | 788.909999999999 |
| 0.26 | 1011.7 | 1078.74 |
| 0.27 | 804.439999999999 | 808.019999999999 |
| 0.28000000000000003 | 908.699999999999 | 831.839999999999 |
| 0.28999999999999998 | 1031.51 | 1063.36 |
| 0.3 | 1075.64 | 1077.92 |
| 0.31 | 1076.47 | 1128.86 |
| 0.32 | 796.43 | 798.09 |
| 0.33 | 835.099999999999 | 813.1 |
| 0.34 | 765.8 | 778.089999999999 |
| 0.35 | 937.339999999999 | 942.799999999999 |
| 0.36 | 825.09 | 812.21 |
| 0.37 | 1202.94 | 1179.34 |
| 0.38 | 1090.32 | 1127.09 |
| 0.39 | 797.35 | 829.28 |
| 0.4 | 928.44 | 911.889999999999 |
| 0.41 | 791.86 | 789.66 |
| 0.42 | 962.079999999999 | 1052.11 |
| 0.43 | 809.91 | 764.31 |
| 0.44 | 1007.12 | 1013.72 |
| 0.45 | 1078.97 | 1074.16 |
| 0.46 | 863.769999999999 | 856.019999999999 |
| 0.47 | 873.82 | 879.409999999999 |
| 0.48 | 1023.93 | 1008.71 |
| 0.49 | 870.859999999999 | 847.259999999999 |
| 0.5 | 1068.02 | 1111.3 |
| 0.51 | 825.02 | 777.189999999999 |
| 0.52 | 1020.91 | 962.429999999999 |
| 0.53 | 864.6 | 812.67 |
| 0.54 | 1022.67 | 1014.09 |
| 0.55000000000000004 | 875.869999999999 | 909.039999999999 |
| 0.56000000000000005 | 870.0 | 862.729999999999 |
| 0.56999999999999995 | 820.009999999999 | 793.479999999999 |
| 0.57999999999999996 | 1257.2 | 1223.54 |
| 0.59 | 916.689999999999 | 888.09 |
| 0.6 | 1078.94 | 1069.99 |
| 0.61 | 931.4 | 947.369999999999 |
| 0.62 | 1003.29 | 965.289999999999 |
| 0.63 | 959.98 | 957.38 |
| 0.64 | 992.759999999999 | 966.189999999999 |
| 0.65 | 1013.74 | 1058.73 |
| 0.66 | 1060.46 | 1051.7 |
| 0.67 | 1021.79 | 985.679999999999 |
| 0.68 | 833.059999999999 | 801.8 |
| 0.69 | 937.36 | 981.179999999999 |
| 0.7 | 869.219999999999 | 881.409999999999 |
| 0.71 | 1045.8 | 1027.9 |
| 0.72 | 859.559999999999 | 843.109999999999 |
| 0.73 | 962.079999999999 | 959.459999999999 |
| 0.74 | 848.61 | 894.83 |
| 0.75 | 792.25 | 803.86 |
| 0.76 | 981.02 | 951.129999999999 |
| 0.77 | 1212.65 | 1160.17 |
| 0.78 | 1050.38 | 1016.15 |
| 0.79 | 1033.63 | 1064.57 |
| 0.8 | 1157.19 | 1151.35 |
| 0.81 | 1113.42 | 1062.05 |
| 0.82 | 940.669999999999 | 949.869999999999 |
| 0.83 | 944.86 | 847.699999999999 |
| 0.84 | 810.769999999999 | 765.0 |
| 0.85 | 945.28 | 963.22 |
| 0.86 | 1165.12 | 1256.07 |
| 0.87 | 949.93 | 1032.08 |
| 0.88 | 1242.43 | 1246.03 |
| 0.89 | 1413.54 | 1366.19 |
| 0.9 | 1033.91 | 1034.12 |
| 0.91 | 1354.67 | 1316.49 |
| 0.92 | 978.02 | 956.61 |
| 0.93 | 812.88 | 842.249999999999 |
| 0.94 | 1322.73 | 1394.95 |
| 0.95 | 1264.33 | 1218.56 |
| 0.96 | 1248.32 | 1195.1 |
| 0.97 | 1109.17 | 1105.1 |
| 0.98 | 1377.04 | 1379.54 |
| 0.99 | 1105.08 | 1112.12 |
| 1 | 1286.19 | 1374.86 |Supplementary Figure S2. DNA methylation levels at the CCGG and CCWGG sites in different regions of genes in E26. X-axis showed the ±2000 bp of transcription start site (TSS), the relative position of gene body and the ±2000 bp of transcription termination site (TTS), respectively. Y-axis showed the relative DNA methylation level.

## Slide 4
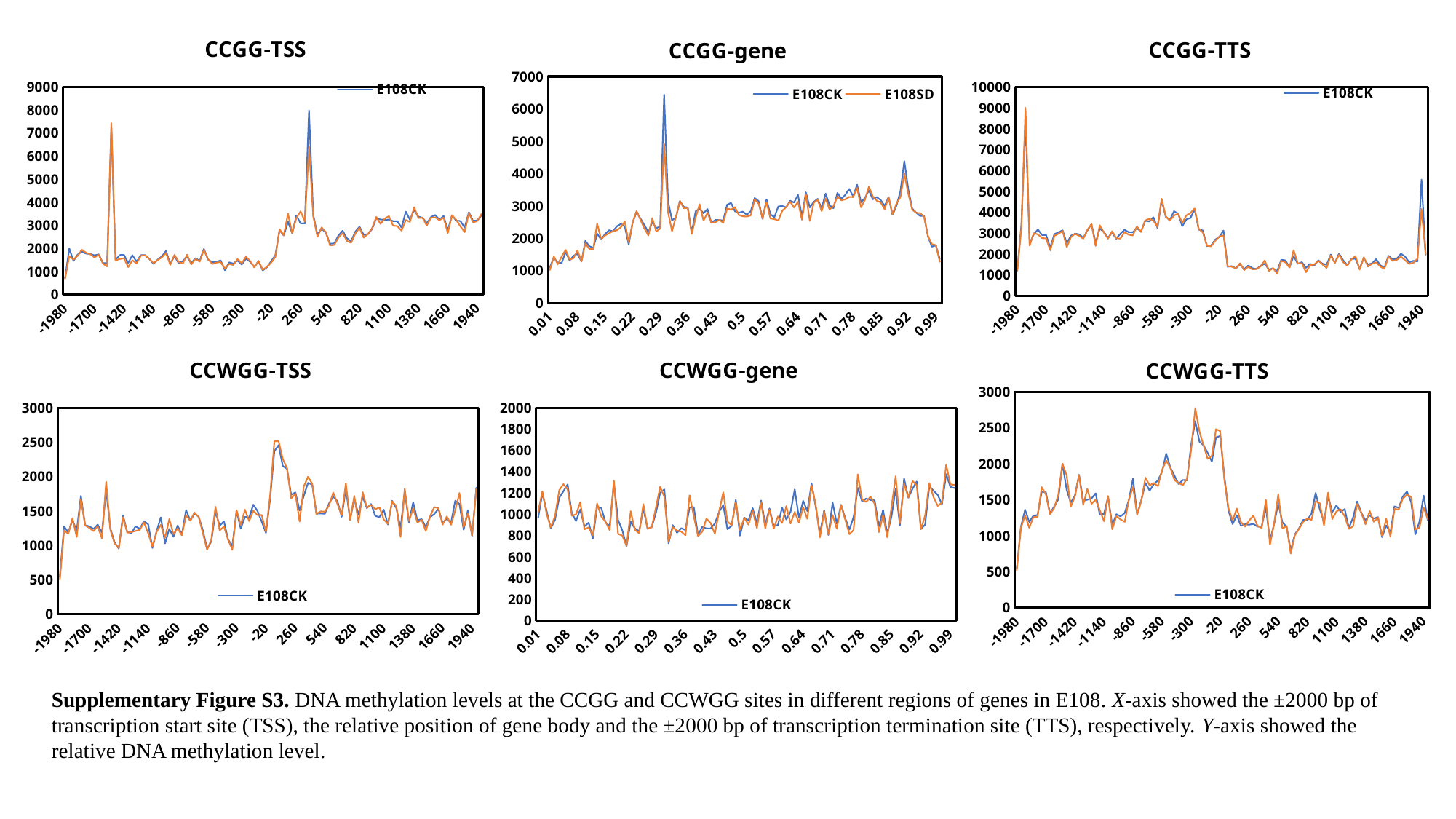

### Chart: CCGG-TSS
| Category | E108CK | E108SD |
|---|---|---|
| -1980 | 701.5 | 687.65 |
| -1940 | 1996.92 | 1667.26 |
| -1900 | 1458.09 | 1515.24 |
| -1860 | 1735.1 | 1690.16 |
| -1820 | 1852.52 | 1943.42 |
| -1780 | 1774.79 | 1811.69 |
| -1740 | 1757.66 | 1744.87 |
| -1700 | 1701.23 | 1613.65 |
| -1660 | 1742.75 | 1730.64 |
| -1620 | 1370.86 | 1344.95 |
| -1580 | 1351.37 | 1221.98 |
| -1540 | 7260.97000000002 | 7430.55000000001 |
| -1500 | 1504.61 | 1483.33 |
| -1460 | 1713.43 | 1539.5 |
| -1420 | 1721.44 | 1576.67 |
| -1380 | 1370.06 | 1190.33 |
| -1340 | 1705.88 | 1487.49 |
| -1300 | 1425.23 | 1345.67 |
| -1260 | 1711.58 | 1682.1 |
| -1220 | 1710.38 | 1707.0 |
| -1180 | 1552.64 | 1550.32 |
| -1140 | 1330.81 | 1364.57 |
| -1100 | 1514.54 | 1494.88 |
| -1060 | 1651.35 | 1613.84 |
| -1020 | 1893.99 | 1804.98 |
| -980 | 1317.5 | 1286.21 |
| -940 | 1674.91 | 1721.27 |
| -900 | 1358.7 | 1409.77 |
| -860 | 1452.76 | 1346.34 |
| -820 | 1634.14 | 1729.35 |
| -780 | 1362.06 | 1311.89 |
| -740 | 1578.92 | 1543.6 |
| -700 | 1459.33 | 1426.09 |
| -660 | 1975.6 | 1936.64 |
| -620 | 1516.74 | 1523.26 |
| -580 | 1400.14 | 1326.75 |
| -540 | 1416.95 | 1386.16 |
| -500 | 1480.58 | 1407.9 |
| -460 | 1053.85 | 1123.52 |
| -420 | 1396.02 | 1329.51 |
| -380 | 1343.02 | 1281.45 |
| -340 | 1477.19 | 1537.51 |
| -300 | 1302.27 | 1358.52 |
| -260 | 1553.77 | 1643.49 |
| -220 | 1425.0 | 1451.67 |
| -180 | 1194.46 | 1178.81 |
| -140 | 1453.17 | 1458.43 |
| -100 | 1049.28 | 1076.44 |
| -60 | 1180.34 | 1201.77 |
| -20 | 1442.11 | 1383.47 |
| 20 | 1706.87 | 1632.79 |
| 60 | 2795.59 | 2826.24 |
| 100 | 2560.37000000001 | 2570.33 |
| 140 | 3154.42 | 3503.87 |
| 180 | 2662.78000000001 | 2664.42 |
| 220 | 3418.87 | 3322.05 |
| 260 | 3089.91 | 3607.80999999999 |
| 300 | 3081.01 | 3170.22 |
| 340 | 7988.71000000003 | 6406.81 |
| 380 | 3460.37000000001 | 3392.34 |
| 420 | 2598.85 | 2509.62 |
| 460 | 2855.38 | 2911.47 |
| 500 | 2679.35 | 2700.72 |
| 540 | 2201.11 | 2128.64 |
| 580 | 2218.3 | 2150.82 |
| 620 | 2559.63 | 2470.28 |
| 660 | 2766.57000000001 | 2666.98 |
| 700 | 2443.81 | 2327.86 |
| 740 | 2304.7 | 2246.12 |
| 780 | 2723.13 | 2644.02 |
| 820 | 2942.53000000001 | 2891.16 |
| 860 | 2585.96000000001 | 2466.14 |
| 900 | 2621.87 | 2623.6 |
| 940 | 2840.47000000001 | 2879.73 |
| 980 | 3302.77 | 3365.38 |
| 1020 | 3253.55000000001 | 3062.68 |
| 1060 | 3239.08000000001 | 3294.41 |
| 1100 | 3248.97 | 3398.4 |
| 1140 | 3179.6 | 2986.42 |
| 1180 | 3176.64 | 2966.1 |
| 1220 | 2904.93000000001 | 2771.64 |
| 1260 | 3595.78 | 3231.5 |
| 1300 | 3241.2 | 3149.75 |
| 1340 | 3672.21000000001 | 3785.37 |
| 1380 | 3375.85 | 3314.54 |
| 1420 | 3327.92000000001 | 3328.85 |
| 1460 | 3089.41 | 2989.74 |
| 1500 | 3360.71000000001 | 3329.25 |
| 1540 | 3446.25000000001 | 3347.7 |
| 1580 | 3244.78000000001 | 3223.44 |
| 1620 | 3403.27000000001 | 3333.51 |
| 1660 | 2783.75 | 2662.87 |
| 1700 | 3432.62 | 3440.2 |
| 1740 | 3200.52000000001 | 3236.99 |
| 1780 | 3187.65 | 2953.94 |
| 1820 | 2903.37 | 2708.76 |
| 1860 | 3571.3 | 3534.74 |
| 1900 | 3192.93000000001 | 3126.13 |
| 1940 | 3215.37 | 3186.91 |
| 1980 | 3430.40000000001 | 3494.28 |
### Chart: CCGG-TTS
| Category | E108CK | E108SD |
|---|---|---|
| -1980 | 1193.76 | 1226.01 |
| -1940 | 3404.47 | 3250.42 |
| -1900 | 8410.96000000002 | 9017.88000000002 |
| -1860 | 2495.98 | 2405.29 |
| -1820 | 2940.28 | 3003.95 |
| -1780 | 3176.91 | 2938.42 |
| -1740 | 2909.18 | 2764.19 |
| -1700 | 2908.77 | 2742.54 |
| -1660 | 2310.12 | 2175.85 |
| -1620 | 2954.85 | 2868.77 |
| -1580 | 3034.05 | 2970.81 |
| -1540 | 3138.41 | 3100.57 |
| -1500 | 2517.49 | 2329.89 |
| -1460 | 2870.22 | 2807.99 |
| -1420 | 2974.20000000001 | 2970.19999999999 |
| -1380 | 2943.35 | 2872.2 |
| -1340 | 2773.09000000001 | 2733.14 |
| -1300 | 3124.08 | 3153.24 |
| -1260 | 3428.03 | 3434.87 |
| -1220 | 2539.29 | 2386.51 |
| -1180 | 3219.24 | 3376.83 |
| -1140 | 3056.95 | 3026.04 |
| -1100 | 2778.02 | 2736.47 |
| -1060 | 3029.41 | 3086.98 |
| -1020 | 2731.07000000001 | 2753.36 |
| -980 | 2973.55 | 2737.73 |
| -940 | 3157.58 | 3046.53 |
| -900 | 3046.07 | 2927.61 |
| -860 | 3042.15 | 2889.68 |
| -820 | 3241.29 | 3328.94 |
| -780 | 3073.32 | 3055.27 |
| -740 | 3587.63000000001 | 3607.71 |
| -700 | 3562.02000000001 | 3681.37 |
| -660 | 3760.31 | 3600.4 |
| -620 | 3248.58 | 3297.14 |
| -580 | 4574.64000000001 | 4645.31999999999 |
| -540 | 3785.50000000001 | 3804.43 |
| -500 | 3624.31000000001 | 3593.73999999999 |
| -460 | 4050.68000000001 | 3848.97 |
| -420 | 3937.64000000001 | 3923.89 |
| -380 | 3332.73000000001 | 3487.59 |
| -340 | 3658.91 | 3852.37 |
| -300 | 3726.76000000001 | 3967.74 |
| -260 | 4151.55 | 4182.74 |
| -220 | 3166.29 | 3204.59 |
| -180 | 3129.32 | 3030.98 |
| -140 | 2378.21000000001 | 2396.12 |
| -100 | 2402.99 | 2365.12 |
| -60 | 2688.06 | 2636.56 |
| -20 | 2823.76000000001 | 2831.05 |
| 20 | 3124.37 | 2895.92 |
| 60 | 1389.17 | 1413.88 |
| 100 | 1415.53 | 1390.24 |
| 140 | 1304.95 | 1313.32 |
| 180 | 1515.72 | 1556.45 |
| 220 | 1270.24 | 1227.51 |
| 260 | 1451.25 | 1389.56 |
| 300 | 1317.83 | 1260.56 |
| 340 | 1278.26 | 1278.77 |
| 380 | 1438.73 | 1403.74 |
| 420 | 1530.5 | 1687.42 |
| 460 | 1253.99 | 1186.84 |
| 500 | 1308.76 | 1322.64 |
| 540 | 1176.55 | 1061.33 |
| 580 | 1722.55 | 1669.93 |
| 620 | 1694.4 | 1602.3 |
| 660 | 1357.9 | 1351.74 |
| 700 | 1903.95 | 2181.21 |
| 740 | 1528.98 | 1548.88 |
| 780 | 1607.14 | 1556.37 |
| 820 | 1336.6 | 1126.84 |
| 860 | 1526.49 | 1469.23 |
| 900 | 1445.51 | 1495.6 |
| 940 | 1695.19 | 1671.2 |
| 980 | 1533.22 | 1500.31 |
| 1020 | 1485.56 | 1334.87 |
| 1060 | 1974.05 | 1931.25 |
| 1100 | 1575.58 | 1565.82 |
| 1140 | 2017.14 | 1970.44 |
| 1180 | 1698.51 | 1611.15 |
| 1220 | 1469.17 | 1442.17 |
| 1260 | 1765.63 | 1740.78 |
| 1300 | 1771.85 | 1900.17 |
| 1340 | 1318.3 | 1252.37 |
| 1380 | 1790.37 | 1842.78 |
| 1420 | 1496.22 | 1397.65 |
| 1460 | 1546.84 | 1528.8 |
| 1500 | 1752.29 | 1600.34 |
| 1540 | 1457.71 | 1396.99 |
| 1580 | 1353.41 | 1288.26 |
| 1620 | 1911.56 | 1863.81 |
| 1660 | 1740.47 | 1669.87 |
| 1700 | 1767.17 | 1717.84 |
| 1740 | 2011.47 | 1866.47 |
| 1780 | 1877.24 | 1704.35 |
| 1820 | 1601.38 | 1520.61 |
| 1860 | 1671.55 | 1573.97 |
| 1900 | 1645.99 | 1768.49 |
| 1940 | 5573.77000000002 | 4156.02999999999 |
| 1980 | 1963.03 | 1989.38 |
### Chart: CCGG-gene
| Category | E108CK | E108SD |
|---|---|---|
| 0.01 | 1104.61 | 1020.05 |
| 0.02 | 1408.62 | 1441.56 |
| 0.03 | 1236.33 | 1199.14 |
| 0.04 | 1240.17 | 1440.21 |
| 0.05 | 1582.34 | 1642.8 |
| 0.06 | 1314.5 | 1339.59 |
| 7.0000000000000007E-2 | 1460.03 | 1386.24 |
| 0.08 | 1519.5 | 1623.97 |
| 0.09 | 1284.35 | 1284.92 |
| 0.1 | 1917.33 | 1843.49 |
| 0.11 | 1759.96 | 1676.58 |
| 0.12 | 1698.61 | 1667.11 |
| 0.13 | 2141.69 | 2453.94 |
| 0.14000000000000001 | 1961.15 | 1979.13 |
| 0.15 | 2131.79 | 2085.91 |
| 0.16 | 2249.09 | 2154.91 |
| 0.17 | 2216.82 | 2219.56 |
| 0.18 | 2368.8 | 2249.5 |
| 0.19 | 2441.09 | 2338.27 |
| 0.2 | 2368.48 | 2520.9 |
| 0.21 | 1808.29 | 1874.56 |
| 0.22 | 2479.73 | 2487.8 |
| 0.23 | 2819.42 | 2841.09 |
| 0.24 | 2605.08 | 2568.21 |
| 0.25 | 2409.14 | 2299.4 |
| 0.26 | 2187.38 | 2087.3 |
| 0.27 | 2504.8 | 2622.99 |
| 0.28000000000000003 | 2319.23 | 2211.51 |
| 0.28999999999999998 | 2356.28 | 2300.71 |
| 0.3 | 6438.68000000002 | 4916.01 |
| 0.31 | 3140.05 | 2801.94 |
| 0.32 | 2556.91 | 2221.7 |
| 0.33 | 2642.42 | 2662.76 |
| 0.34 | 3150.7 | 3147.03 |
| 0.35 | 2965.03 | 2928.22 |
| 0.36 | 2952.92 | 2939.04 |
| 0.37 | 2173.29 | 2133.89 |
| 0.38 | 2831.76 | 2635.73 |
| 0.39 | 2920.55 | 3053.24999999999 |
| 0.4 | 2772.27 | 2543.88 |
| 0.41 | 2902.26 | 2786.37 |
| 0.42 | 2476.34 | 2474.9 |
| 0.43 | 2565.44000000001 | 2494.53 |
| 0.44 | 2567.73 | 2568.97 |
| 0.45 | 2547.51 | 2480.38 |
| 0.46 | 3038.27 | 2921.49 |
| 0.47 | 3092.05 | 2885.7 |
| 0.48 | 2832.45 | 2959.3 |
| 0.49 | 2789.83 | 2721.38 |
| 0.5 | 2822.31 | 2678.32 |
| 0.51 | 2726.59 | 2673.59 |
| 0.52 | 2835.18 | 2704.6 |
| 0.53 | 3241.8 | 3198.45 |
| 0.54 | 3149.1 | 3072.23 |
| 0.55000000000000004 | 2600.86 | 2612.79 |
| 0.56000000000000005 | 3199.85 | 3123.34 |
| 0.56999999999999995 | 2739.93 | 2611.31 |
| 0.57999999999999996 | 2654.52 | 2591.8 |
| 0.59 | 2983.8 | 2548.68 |
| 0.6 | 2996.76 | 2849.22 |
| 0.61 | 2953.71 | 2964.06 |
| 0.62 | 3158.69 | 3129.42 |
| 0.63 | 3103.76 | 2952.35 |
| 0.64 | 3337.73 | 3126.79 |
| 0.65 | 2666.82 | 2572.27 |
| 0.66 | 3418.44000000001 | 3356.44 |
| 0.67 | 2951.09 | 2533.72 |
| 0.68 | 3122.4 | 3099.15 |
| 0.69 | 3217.74 | 3186.87 |
| 0.7 | 2922.08 | 2839.64 |
| 0.71 | 3381.24 | 3232.22 |
| 0.72 | 3020.04 | 2892.46 |
| 0.73 | 2929.71 | 2990.33 |
| 0.74 | 3401.82 | 3288.82 |
| 0.75 | 3219.71 | 3174.71 |
| 0.76 | 3340.77 | 3203.14 |
| 0.77 | 3525.58 | 3277.54 |
| 0.78 | 3293.95 | 3271.34 |
| 0.79 | 3657.01 | 3558.45 |
| 0.8 | 3110.70000000001 | 2956.01 |
| 0.81 | 3247.91 | 3188.96 |
| 0.82 | 3480.65 | 3600.95 |
| 0.83 | 3204.33 | 3290.98 |
| 0.84 | 3271.93 | 3161.99 |
| 0.85 | 3180.37 | 3107.2 |
| 0.86 | 2997.16 | 2899.17 |
| 0.87 | 3272.23 | 3260.65 |
| 0.88 | 2721.57 | 2745.78 |
| 0.89 | 3006.94 | 3070.12 |
| 0.9 | 3449.42 | 3274.48 |
| 0.91 | 4385.62 | 3993.44 |
| 0.92 | 3516.13 | 3376.71 |
| 0.93 | 2917.88 | 2885.07 |
| 0.94 | 2795.87 | 2785.75 |
| 0.95 | 2688.88 | 2777.66 |
| 0.96 | 2697.34 | 2670.28 |
| 0.97 | 2049.94 | 2071.81 |
| 0.98 | 1741.26 | 1815.7 |
| 0.99 | 1783.08 | 1790.16 |
| 1 | 1355.32 | 1273.37 |
### Chart: CCWGG-TSS
| Category | E108CK | E108SD |
|---|---|---|
| -1980 | 555.03 | 505.15 |
| -1940 | 1278.06 | 1218.32 |
| -1900 | 1190.47 | 1169.99 |
| -1860 | 1370.54 | 1394.72 |
| -1820 | 1212.96 | 1122.27 |
| -1780 | 1721.55 | 1674.49 |
| -1740 | 1296.24 | 1292.0 |
| -1700 | 1277.28 | 1255.87 |
| -1660 | 1241.31 | 1208.85 |
| -1620 | 1303.01 | 1277.99 |
| -1580 | 1183.97 | 1104.14 |
| -1540 | 1805.71 | 1926.77 |
| -1500 | 1247.77 | 1226.29 |
| -1460 | 1045.0 | 1029.06 |
| -1420 | 954.89 | 962.689999999999 |
| -1380 | 1438.87 | 1411.5 |
| -1340 | 1196.53 | 1186.09 |
| -1300 | 1178.26 | 1195.45 |
| -1260 | 1278.42 | 1210.99 |
| -1220 | 1243.26 | 1227.01 |
| -1180 | 1354.38 | 1336.35 |
| -1140 | 1306.32 | 1175.97 |
| -1100 | 963.39 | 984.11 |
| -1060 | 1210.94 | 1198.17 |
| -1020 | 1408.14 | 1301.47 |
| -980 | 1029.65 | 1111.62 |
| -940 | 1240.56 | 1382.68 |
| -900 | 1126.55 | 1164.62 |
| -860 | 1289.35 | 1247.8 |
| -820 | 1157.63 | 1147.18 |
| -780 | 1514.73 | 1437.67 |
| -740 | 1361.67 | 1360.51 |
| -700 | 1460.68 | 1478.62 |
| -660 | 1419.43 | 1412.19 |
| -620 | 1217.82 | 1176.66 |
| -580 | 953.659999999999 | 937.84 |
| -540 | 1057.88 | 1084.07 |
| -500 | 1488.87 | 1562.42 |
| -460 | 1285.66 | 1216.92 |
| -420 | 1353.13 | 1277.91 |
| -380 | 1086.14 | 1082.09 |
| -340 | 994.479999999999 | 936.499999999999 |
| -300 | 1503.85 | 1513.49 |
| -260 | 1245.33 | 1298.13 |
| -220 | 1416.2 | 1519.55 |
| -180 | 1407.33 | 1354.47 |
| -140 | 1592.75 | 1506.14 |
| -100 | 1500.24 | 1442.38 |
| -60 | 1346.34 | 1442.36 |
| -20 | 1181.94 | 1202.22 |
| 20 | 1684.06 | 1730.83 |
| 60 | 2371.53 | 2514.48 |
| 100 | 2459.96 | 2517.78 |
| 140 | 2155.78 | 2258.79 |
| 180 | 2115.43 | 2122.54 |
| 220 | 1736.47 | 1683.83 |
| 260 | 1772.69 | 1752.91 |
| 300 | 1508.7 | 1349.78 |
| 340 | 1721.94 | 1859.01 |
| 380 | 1910.18 | 1999.14 |
| 420 | 1886.87 | 1897.93 |
| 460 | 1462.13 | 1459.79 |
| 500 | 1465.85 | 1495.4 |
| 540 | 1460.67 | 1491.37 |
| 580 | 1611.67 | 1579.88 |
| 620 | 1711.75 | 1770.43 |
| 660 | 1642.37 | 1604.01 |
| 700 | 1417.38 | 1438.99 |
| 740 | 1824.49 | 1904.63 |
| 780 | 1373.39 | 1369.14 |
| 820 | 1677.55 | 1720.7 |
| 860 | 1448.85 | 1330.23 |
| 900 | 1709.39 | 1774.38 |
| 940 | 1543.03 | 1547.72 |
| 980 | 1601.93 | 1591.94 |
| 1020 | 1428.67 | 1526.81 |
| 1060 | 1413.35 | 1552.3 |
| 1100 | 1520.37 | 1377.94 |
| 1140 | 1304.64 | 1314.83 |
| 1180 | 1649.16 | 1640.95 |
| 1220 | 1542.55 | 1570.49 |
| 1260 | 1253.81 | 1124.25 |
| 1300 | 1794.23 | 1823.36 |
| 1340 | 1332.11 | 1354.46 |
| 1380 | 1628.97 | 1538.89 |
| 1420 | 1366.06 | 1334.94 |
| 1460 | 1383.82 | 1373.2 |
| 1500 | 1267.1 | 1208.89 |
| 1540 | 1411.64 | 1422.06 |
| 1580 | 1456.61 | 1557.66 |
| 1620 | 1533.71 | 1543.01 |
| 1660 | 1329.33 | 1302.76 |
| 1700 | 1391.96 | 1422.15 |
| 1740 | 1329.6 | 1300.74 |
| 1780 | 1652.48 | 1510.81 |
| 1820 | 1603.58 | 1763.69 |
| 1860 | 1228.21 | 1268.56 |
| 1900 | 1511.44 | 1464.48 |
| 1940 | 1138.98 | 1147.76 |
| 1980 | 1840.26 | 1816.06 |
### Chart: CCWGG-gene
| Category | E108CK | E108SD |
|---|---|---|
| 0.01 | 966.63 | 1023.71 |
| 0.02 | 1198.44 | 1216.29 |
| 0.03 | 1033.24 | 1004.94 |
| 0.04 | 868.12 | 878.139999999999 |
| 0.05 | 947.629999999999 | 981.509999999999 |
| 0.06 | 1155.65 | 1226.38 |
| 7.0000000000000007E-2 | 1217.07 | 1283.37 |
| 0.08 | 1281.3 | 1233.74 |
| 0.09 | 1009.46 | 986.179999999999 |
| 0.1 | 937.47 | 999.54 |
| 0.11 | 1044.51 | 1112.98 |
| 0.12 | 885.779999999999 | 858.7 |
| 0.13 | 920.869999999999 | 876.779999999999 |
| 0.14000000000000001 | 771.51 | 803.0 |
| 0.15 | 1071.24 | 1101.5 |
| 0.16 | 1059.47 | 980.089999999999 |
| 0.17 | 926.609999999999 | 930.459999999999 |
| 0.18 | 888.219999999999 | 851.269999999999 |
| 0.19 | 1293.8 | 1316.91 |
| 0.2 | 946.329999999999 | 815.059999999999 |
| 0.21 | 850.23 | 800.289999999999 |
| 0.22 | 699.93 | 703.61 |
| 0.23 | 929.069999999999 | 1029.07 |
| 0.24 | 867.559999999999 | 854.58 |
| 0.25 | 839.719999999999 | 821.739999999999 |
| 0.26 | 1054.26 | 1096.19 |
| 0.27 | 863.949999999999 | 863.369999999999 |
| 0.28000000000000003 | 879.329999999999 | 879.449999999999 |
| 0.28999999999999998 | 1014.64 | 1064.01 |
| 0.3 | 1202.02 | 1258.57 |
| 0.31 | 1234.37 | 1164.64 |
| 0.32 | 726.989999999999 | 743.96 |
| 0.33 | 898.279999999999 | 878.1 |
| 0.34 | 826.479999999999 | 847.229999999999 |
| 0.35 | 869.239999999999 | 837.87 |
| 0.36 | 852.209999999999 | 803.6 |
| 0.37 | 1064.35 | 1178.64 |
| 0.38 | 1066.71 | 982.78 |
| 0.39 | 806.169999999999 | 794.939999999999 |
| 0.4 | 882.419999999999 | 836.48 |
| 0.41 | 867.199999999999 | 959.28 |
| 0.42 | 867.229999999999 | 917.629999999999 |
| 0.43 | 913.249999999999 | 815.78 |
| 0.44 | 1023.47 | 1025.05 |
| 0.45 | 1087.37 | 1206.92 |
| 0.46 | 860.339999999999 | 931.79 |
| 0.47 | 891.369999999999 | 897.539999999999 |
| 0.48 | 1135.43 | 1111.63 |
| 0.49 | 799.339999999999 | 853.289999999999 |
| 0.5 | 969.329999999999 | 966.699999999999 |
| 0.51 | 942.759999999999 | 902.939999999999 |
| 0.52 | 1057.81 | 1036.41 |
| 0.53 | 915.259999999999 | 870.05 |
| 0.54 | 1129.75 | 1110.88 |
| 0.55000000000000004 | 903.939999999999 | 869.379999999999 |
| 0.56000000000000005 | 1055.06 | 1045.85 |
| 0.56999999999999995 | 889.379999999999 | 865.27 |
| 0.57999999999999996 | 898.64 | 980.15 |
| 0.59 | 1064.24 | 918.359999999999 |
| 0.6 | 950.399999999999 | 1077.39 |
| 0.61 | 1022.34 | 913.009999999999 |
| 0.62 | 1234.75 | 1022.34 |
| 0.63 | 967.77 | 920.379999999999 |
| 0.64 | 1126.15 | 1066.62 |
| 0.65 | 1029.57 | 957.29 |
| 0.66 | 1289.38 | 1272.61 |
| 0.67 | 1070.42 | 1090.13 |
| 0.68 | 819.539999999999 | 782.829999999999 |
| 0.69 | 1039.16 | 1028.4 |
| 0.7 | 805.389999999999 | 812.33 |
| 0.71 | 1110.79 | 992.829999999999 |
| 0.72 | 910.819999999999 | 863.329999999999 |
| 0.73 | 1089.35 | 1086.75 |
| 0.74 | 965.349999999999 | 951.3 |
| 0.75 | 856.649999999999 | 812.999999999999 |
| 0.76 | 975.469999999998 | 852.609999999999 |
| 0.77 | 1249.34 | 1375.3 |
| 0.78 | 1122.15 | 1140.38 |
| 0.79 | 1147.48 | 1116.31 |
| 0.8 | 1134.68 | 1166.63 |
| 0.81 | 1130.97 | 1098.11 |
| 0.82 | 885.819999999999 | 832.5 |
| 0.83 | 1040.45 | 990.179999999999 |
| 0.84 | 819.049999999999 | 783.51 |
| 0.85 | 978.289999999999 | 1067.32 |
| 0.86 | 1238.49 | 1358.51 |
| 0.87 | 896.259999999999 | 916.97 |
| 0.88 | 1335.8 | 1287.36 |
| 0.89 | 1155.6 | 1159.22 |
| 0.9 | 1239.66 | 1314.2 |
| 0.91 | 1309.05 | 1271.95 |
| 0.92 | 861.559999999999 | 858.65 |
| 0.93 | 902.309999999999 | 989.5 |
| 0.94 | 1263.03 | 1293.4 |
| 0.95 | 1221.02 | 1161.91 |
| 0.96 | 1182.6 | 1078.72 |
| 0.97 | 1094.33 | 1109.63 |
| 0.98 | 1376.6 | 1465.88 |
| 0.99 | 1257.39 | 1282.0 |
| 1 | 1246.97 | 1276.62 |
### Chart: CCWGG-TTS
| Category | E108CK | E108SD |
|---|---|---|
| -1980 | 546.96 | 520.56 |
| -1940 | 1124.94 | 1112.26 |
| -1900 | 1362.46 | 1284.72 |
| -1860 | 1192.73 | 1111.57 |
| -1820 | 1278.45 | 1258.46 |
| -1780 | 1289.71 | 1263.86 |
| -1740 | 1611.25 | 1676.53 |
| -1700 | 1610.01 | 1578.46 |
| -1660 | 1309.1 | 1299.42 |
| -1620 | 1406.82 | 1385.39 |
| -1580 | 1506.66 | 1571.11 |
| -1540 | 2004.3 | 2003.84 |
| -1500 | 1644.83 | 1844.88 |
| -1460 | 1461.87 | 1406.15 |
| -1420 | 1557.88 | 1542.97 |
| -1380 | 1850.08 | 1842.22 |
| -1340 | 1485.29 | 1430.89 |
| -1300 | 1502.66 | 1652.3 |
| -1260 | 1519.63 | 1445.03 |
| -1220 | 1590.35 | 1503.43 |
| -1180 | 1292.53 | 1355.76 |
| -1140 | 1304.97 | 1202.81 |
| -1100 | 1540.99 | 1553.72 |
| -1060 | 1151.51 | 1090.74 |
| -1020 | 1299.79 | 1274.58 |
| -980 | 1270.73 | 1228.31 |
| -940 | 1317.52 | 1194.14 |
| -900 | 1500.59 | 1502.69 |
| -860 | 1795.94 | 1680.49 |
| -820 | 1297.79 | 1294.68 |
| -780 | 1496.12 | 1488.59 |
| -740 | 1734.43 | 1809.34 |
| -700 | 1628.27 | 1701.9 |
| -660 | 1716.7 | 1734.83 |
| -620 | 1770.81 | 1690.54 |
| -580 | 1891.96 | 1916.69 |
| -540 | 2143.61 | 2046.76 |
| -500 | 1948.63 | 1944.19 |
| -460 | 1836.97 | 1775.79 |
| -420 | 1720.71 | 1733.45 |
| -380 | 1777.3 | 1705.35 |
| -340 | 1770.34 | 1793.17 |
| -300 | 2269.73 | 2193.74 |
| -260 | 2595.19 | 2777.45 |
| -220 | 2310.11 | 2451.35 |
| -180 | 2259.63 | 2254.75 |
| -140 | 2154.65 | 2070.92 |
| -100 | 2033.43 | 2116.04 |
| -60 | 2371.49 | 2484.21 |
| -20 | 2388.09 | 2457.39 |
| 20 | 1837.44 | 1781.81 |
| 60 | 1349.52 | 1383.4 |
| 100 | 1162.87 | 1215.55 |
| 140 | 1285.7 | 1378.64 |
| 180 | 1139.07 | 1192.06 |
| 220 | 1159.7 | 1127.72 |
| 260 | 1154.43 | 1213.58 |
| 300 | 1166.14 | 1282.01 |
| 340 | 1127.76 | 1137.81 |
| 380 | 1128.54 | 1108.28 |
| 420 | 1398.04 | 1500.74 |
| 460 | 935.92 | 876.739999999999 |
| 500 | 1165.76 | 1169.98 |
| 540 | 1450.17 | 1576.56 |
| 580 | 1186.79 | 1100.9 |
| 620 | 1126.62 | 1136.45 |
| 660 | 796.86 | 753.3 |
| 700 | 1019.87 | 1006.26 |
| 740 | 1103.55 | 1098.17 |
| 780 | 1221.41 | 1192.76 |
| 820 | 1222.27 | 1241.11 |
| 860 | 1310.4 | 1221.57 |
| 900 | 1597.21 | 1483.29 |
| 940 | 1368.97 | 1453.16 |
| 980 | 1197.25 | 1147.22 |
| 1020 | 1549.9 | 1600.56 |
| 1060 | 1329.43 | 1233.02 |
| 1100 | 1422.26 | 1338.32 |
| 1140 | 1333.35 | 1370.59 |
| 1180 | 1370.96 | 1272.64 |
| 1220 | 1106.77 | 1096.16 |
| 1260 | 1253.42 | 1131.08 |
| 1300 | 1478.4 | 1444.4 |
| 1340 | 1320.83 | 1316.22 |
| 1380 | 1216.31 | 1161.23 |
| 1420 | 1285.73 | 1345.08 |
| 1460 | 1240.11 | 1194.77 |
| 1500 | 1259.85 | 1255.84 |
| 1540 | 981.949999999999 | 1000.88 |
| 1580 | 1144.75 | 1236.37 |
| 1620 | 1042.12 | 986.799999999999 |
| 1660 | 1410.18 | 1374.65 |
| 1700 | 1388.31 | 1363.21 |
| 1740 | 1546.66 | 1519.56 |
| 1780 | 1614.87 | 1572.54 |
| 1820 | 1466.7 | 1540.3 |
| 1860 | 1019.92 | 1087.51 |
| 1900 | 1196.06 | 1114.32 |
| 1940 | 1561.21 | 1394.75 |
| 1980 | 1214.32 | 1229.68 |Supplementary Figure S3. DNA methylation levels at the CCGG and CCWGG sites in different regions of genes in E108. X-axis showed the ±2000 bp of transcription start site (TSS), the relative position of gene body and the ±2000 bp of transcription termination site (TTS), respectively. Y-axis showed the relative DNA methylation level.

## Slide 5
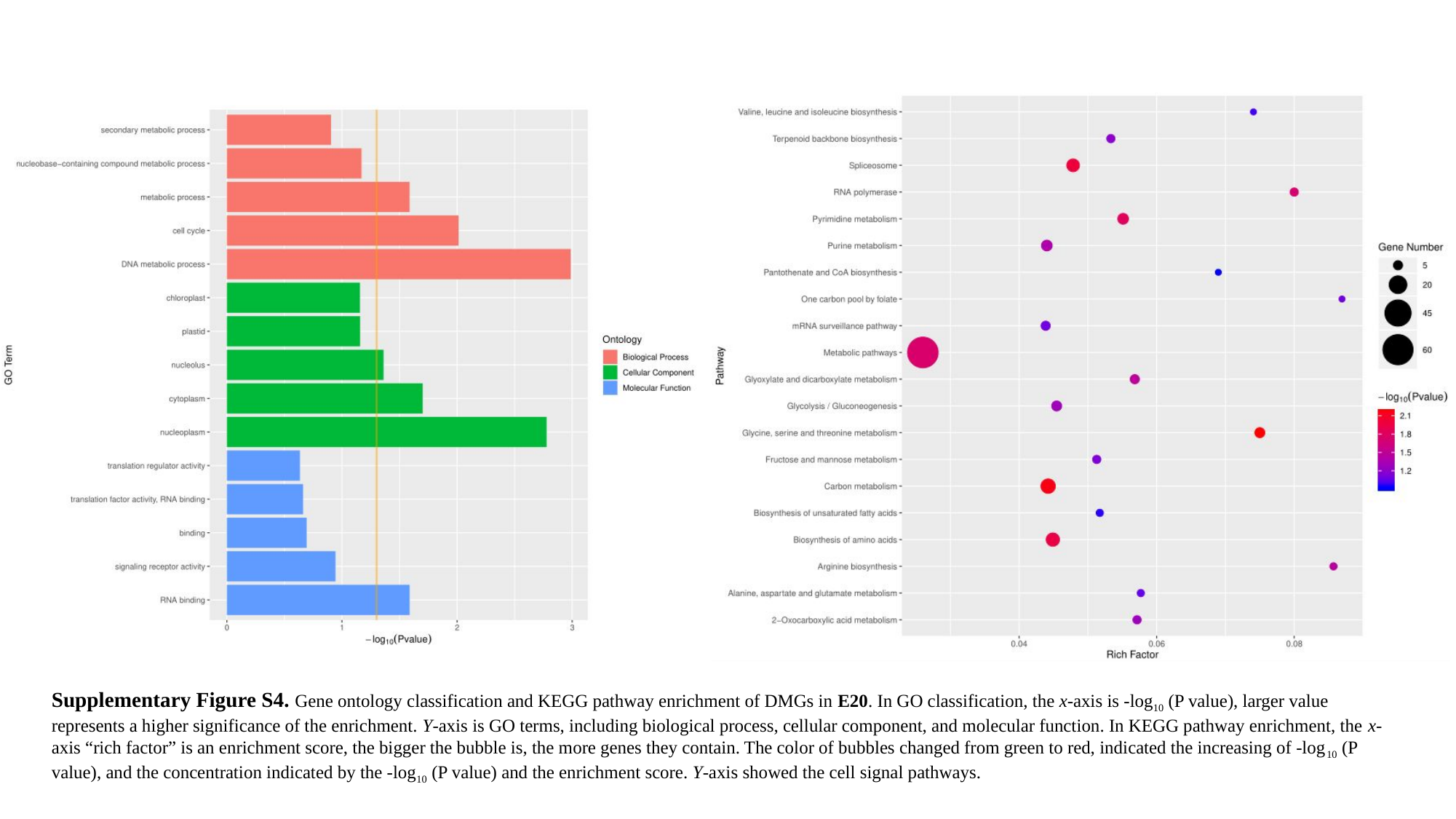

Supplementary Figure S4. Gene ontology classification and KEGG pathway enrichment of DMGs in E20. In GO classification, the x-axis is -log10 (P value), larger value represents a higher significance of the enrichment. Y-axis is GO terms, including biological process, cellular component, and molecular function. In KEGG pathway enrichment, the x-axis “rich factor” is an enrichment score, the bigger the bubble is, the more genes they contain. The color of bubbles changed from green to red, indicated the increasing of -log10 (P value), and the concentration indicated by the -log10 (P value) and the enrichment score. Y-axis showed the cell signal pathways.

## Slide 6
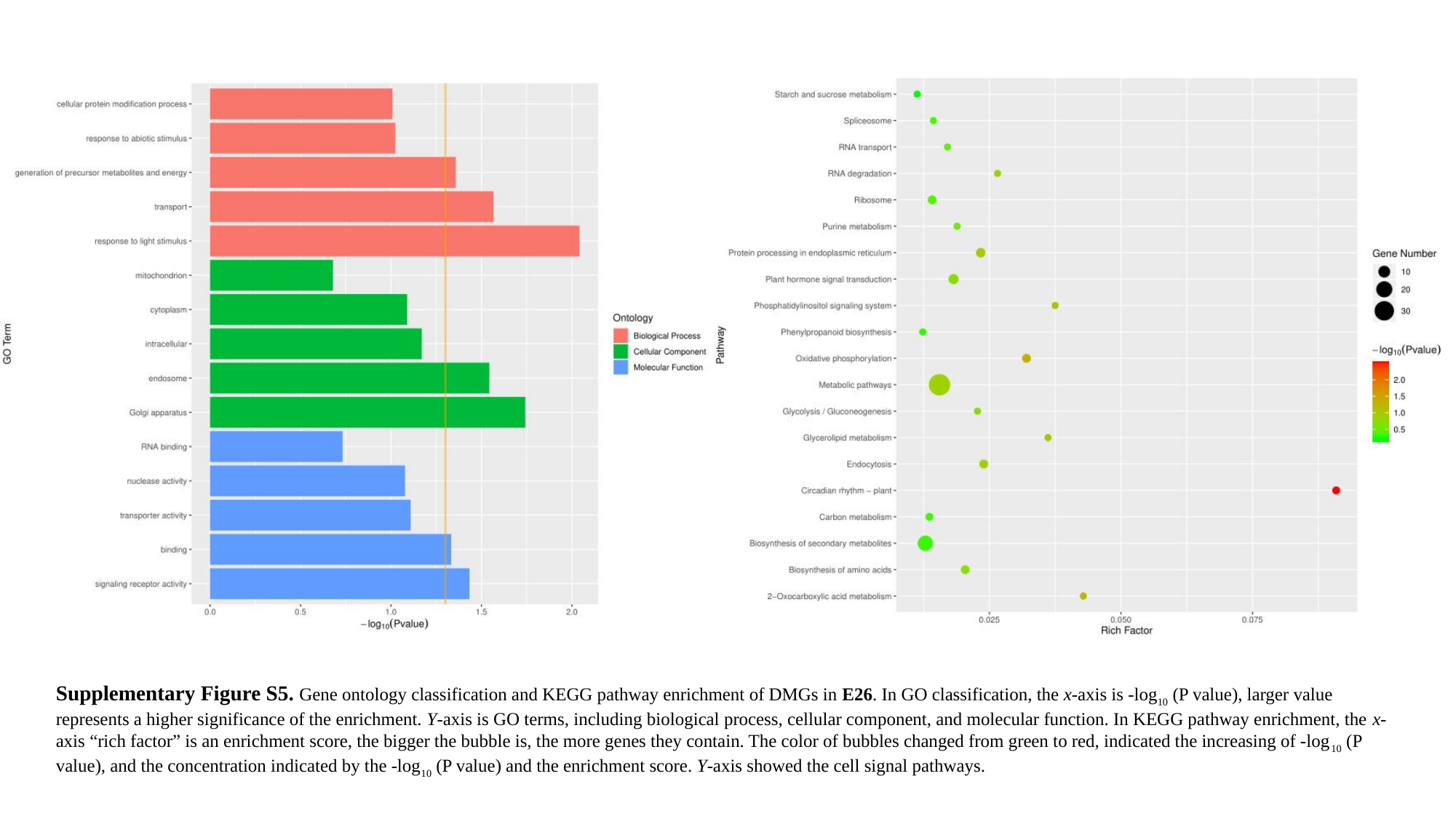

Supplementary Figure S5. Gene ontology classification and KEGG pathway enrichment of DMGs in E26. In GO classification, the x-axis is -log10 (P value), larger value represents a higher significance of the enrichment. Y-axis is GO terms, including biological process, cellular component, and molecular function. In KEGG pathway enrichment, the x-axis “rich factor” is an enrichment score, the bigger the bubble is, the more genes they contain. The color of bubbles changed from green to red, indicated the increasing of -log10 (P value), and the concentration indicated by the -log10 (P value) and the enrichment score. Y-axis showed the cell signal pathways.

## Slide 7
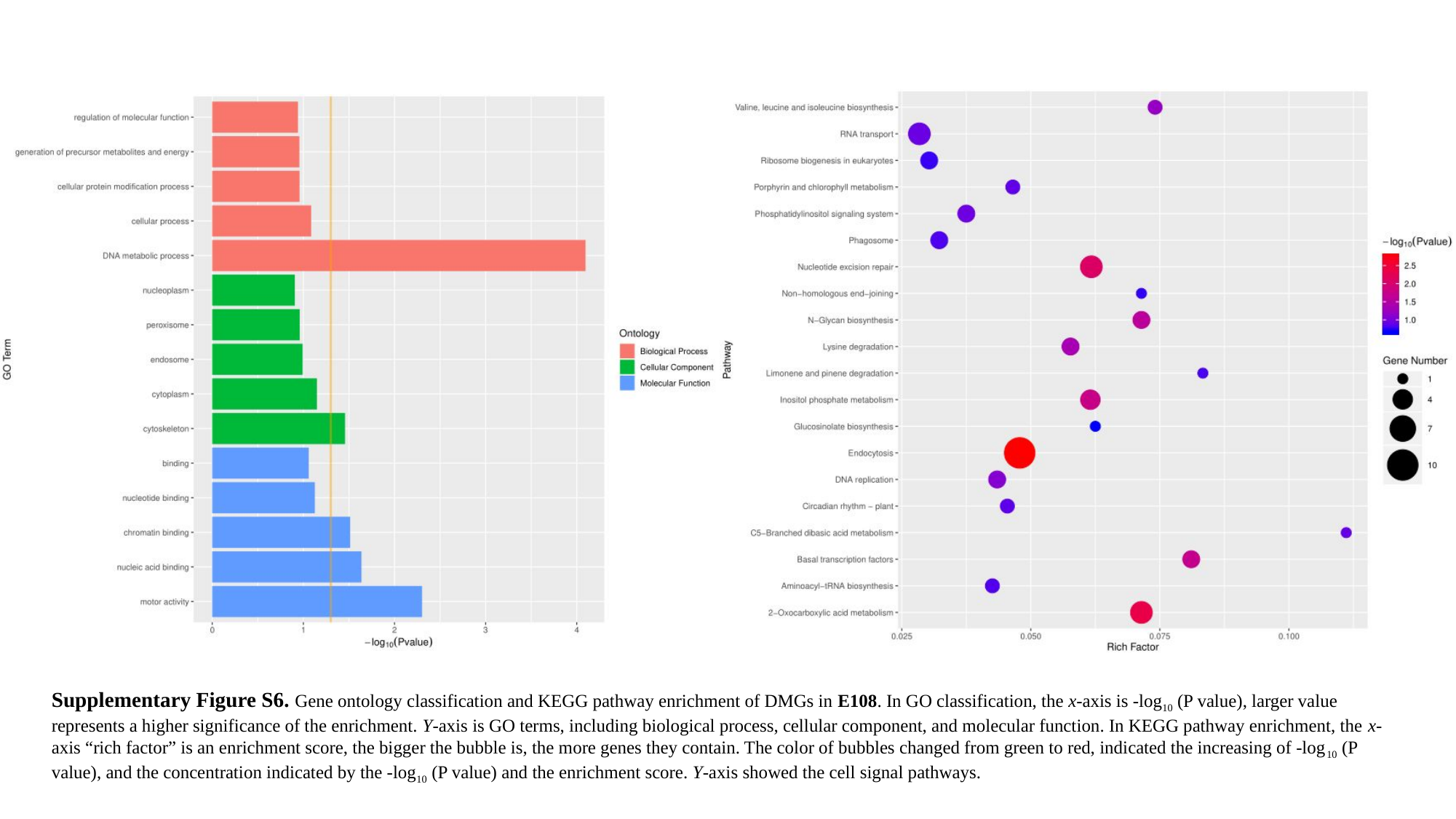

Supplementary Figure S6. Gene ontology classification and KEGG pathway enrichment of DMGs in E108. In GO classification, the x-axis is -log10 (P value), larger value represents a higher significance of the enrichment. Y-axis is GO terms, including biological process, cellular component, and molecular function. In KEGG pathway enrichment, the x-axis “rich factor” is an enrichment score, the bigger the bubble is, the more genes they contain. The color of bubbles changed from green to red, indicated the increasing of -log10 (P value), and the concentration indicated by the -log10 (P value) and the enrichment score. Y-axis showed the cell signal pathways.
