# Supplementary material for: DNA methylation affects photoperiodic tuberization in potato (Solanum tuberosum L.) by mediating the expression of genes related to the photoperiod and GA pathways
Source: Hortic Res. 2021 Sep 1;8:181. doi: 10.1038/s41438-021-00619-7 (PMC8408180; doi:10.1038/s41438-021-00619-7)
Supplement: Supplementary file 3 — Supplementary Table S1-S7 [file 41438_2021_619_MOESM3_ESM.docx]

Article title: **DNA Methylation affects photoperiodic-tuberization in potato (*Solanum tuberosum* L.) by mediating the expression of the genes related to photoperiod and GA pathway**

Journal name: **Horticulture Research**

Author names: Yanjun Ai et al.

Correspondence to **Jun Zhou** (e-mail: zhoujun820830@163.com); **Jun Liu** (e-mail: liujun@mail.hzau.edu.cn)

**Supplementary Table S1: Primer sequences of tuberization marker genes used in qRT-PCR analysis.**

| **Primer name** | **Target gene** | **Sequence (5'-3')** | **Length** | **Description** |
| --- | --- | --- | --- | --- |
| qStSP6A-s | PGSC0003DMT400060057 | AGGTTGGCGTCAAAATTTCAA | 101 | Flowering locus T protein |
| qStSP6A-a | PGSC0003DMT400060057 | TGCCACTCTCCCTATGGGAA | 101 | Flowering locus T protein |
| qStCO-s | PGSC0003DMT400026065 | GTCTCATGGCTTCTCGCCAC | 100 | CONSTANS |
| qStCO-a | PGSC0003DMT400026065 | GCACAAAGTGAGGCAGCATC | 100 | CONSTANS |
| qStSP5G-N-F | StSP5G | GGTGTGTAGACTTTGGTGTGGTTT | 64 | Flowering locus T protein |
| qStSP5G-N-R | StSP5G | GGCCTCAAGGCACATCCAT | 64 | Flowering locus T protein |
| qStCDF1-F | PGSC0003DMT400047370 | TGCAGACTCGTCGATTGAAC | 130 | Zinc finger protein |
| qStCDF1-R | PGSC0003DMT400047370 | GAGTGCCTTTTCCTCACTCG | 130 | Zinc finger protein |
| RTef1αF | Elongation factor 1-alpha | ATTGGAAACGGATATGCTCCA | 101 | Internal reference gene |
| RTef1αR | Elongation factor 1-alpha | TCCTTACCTGAACGCCTGTCA | 101 | Internal reference gene |

**Supplementary Table S2: Quantity of sequencing data and mapping ratios.**

| **Samples** | **MethylRAD tags** | **Quantity of data** | **Mapping tags** | **Ratio%** |
| --- | --- | --- | --- | --- |
| E20CK | 16,099,973 | 507,424,995 | 5,802,300 | 36.04% |
| E20SD | 15,424,714 | 486,341,370 | 5,424,189 | 35.17% |
| E26CK | 16,450,845 | 518,208,346 | 5,359,096 | 32.58% |
| E26SD | 15,213,368 | 479,233,323 | 4,979,447 | 32.73% |
| E108CK | 15,190,732 | 478,727,069 | 5,340,003 | 35.15% |
| E108SD | 12,909,077 | 405,420,909 | 3,110,736 | 24.10% |

**Supplementary Table S3: Summary of methylation site coverage.**

| **Samples** | **CCGG** | | **CCWGG** | |
| --- | --- | --- | --- | --- |
|  | **Number of sites** | **Average depth** | **Number of sites** | **Average depth** |
| E20CK | 115,856 | 24.17 | 99,544 | 26.54 |
| E20SD | 108,137 | 24.48 | 91,432 | 26.51 |
| E26CK | 117,196 | 22.87 | 99,046 | 23.59 |
| E26SD | 108,753 | 21.29 | 96,231 | 24.48 |
| E108CK | 109,013 | 23.63 | 93,133 | 26.12 |
| E108SD | 86,635 | 16.45 | 77,908 | 18.58 |

**Supplementary Table S4: Summary of methylation ratios of CCGG/CCWGG sites.**

| **Samples** | **CCGG** | | **CCWGG** | | **Total (CCGG+CCWGG)** | |
| --- | --- | --- | --- | --- | --- | --- |
|  | **Sites** | **Ratio%** | **Sites** | **Ratio%** | **Sites** | **Ratio%** |
| E20CK | 115,856 | 12.65 | 99,544 | 10.87 | 215,400 | 23.52 |
| E20SD | 108,137 | 11.80 | 91,432 | 9.98 | 199,569 | 21.78 |
| E26CK | 117,196 | 12.79 | 99,046 | 10.81 | 216,242 | 23.60 |
| E26SD | 108,753 | 11.87 | 96,231 | 10.50 | 204,984 | 22.37 |
| E108CK | 109,013 | 11.90 | 93,133 | 10.17 | 202,146 | 22.07 |
| E108SD | 86,635 | 9.46 | 77,908 | 9.46 | 164,543 | 17.96 |
| Genome | 496,160 | - | 420,010 | - | 916,170 | - |

**Supplementary Table S5: Summary of distributions of CCGG/CCWGG sites on different gene function components.**

| **Samples** | | **E20CK** | **E20SD** | **E26CK** | **E26SD** | **E108CK** | **E108SD** |
| --- | --- | --- | --- | --- | --- | --- | --- |
| **CCGG** | **Intergenic** | 78165 | 72863 | 78990 | 72329 | 72828 | 55180 |
|  | **Upstream** | 9416 | 8725 | 9622 | 8872 | 8991 | 6830 |
|  | **Exon** | 14916 | 13709 | 15062 | 14125 | 14558 | 11780 |
|  | **Intron** | 10364 | 9508 | 10497 | 9640 | 9891 | 7745 |
|  | **Utr3prime** | 1930 | 1750 | 1991 | 1839 | 1896 | 1504 |
|  | **Utr5prime** | 1245 | 1165 | 1265 | 1185 | 1241 | 967 |
| **CCWGG** | **Intergenic** | 78997 | 72133 | 78616 | 76362 | 73752 | 60187 |
|  | **Upstream** | 6830 | 6209 | 6737 | 6482 | 6370 | 5046 |
|  | **Exon** | 4904 | 4515 | 4630 | 4556 | 4548 | 3534 |
|  | **Intron** | 5916 | 5374 | 5632 | 5466 | 5613 | 4389 |
|  | **Utr3prime** | 527 | 467 | 472 | 452 | 465 | 329 |
|  | **Utr5prime** | 339 | 313 | 301 | 283 | 307 | 221 |

**Note:** Upstream, Exon and Intron indicated the regions of upstream 2000 bp of the transcription start site (TSS), the whole exons of genes and the whole introns of genes, respectively. Utr3prime and Utr5prime indicated the regions at the 3' end and 5' end of a mature transcript that is not translated into a protein. “Intergenic” indicated the intergenic regions.

**Supplementary Table S6: Numbers of differentially methylated genes (DMGs) in SD group relative to CK group of each genotype.**

| **Name** | **Compare** | **CCGG** | | | **CCWGG** | | | **CCGG+CCWGG** | | |
| --- | --- | --- | --- | --- | --- | --- | --- | --- | --- | --- |
|  |  | **Total** | **hyper** | **hypo** | **Total** | **hyper** | **hypo** | **Total** | **hyper** | **hypo** |
| **E20** | E20SD vs E20CK | 514 | 132 (25.7%) | 382 (74.3%) | 276 | 73 (26.4%) | 203 (73.6%) | 785 | 202 (25.7%) | 583 (74.3%) |
| **E26** | E26SD vs E26CK | 332 | 148 (44.6%) | 184 (55.4%) | 168 | 68 (40.5%) | 100 (59.5%) | 500 | 216 (43.2%) | 284 (56.8%) |
| **E108** | E108SD vs E108CK | 431 | 264 (61.3%) | 167 (38.7%) | 204 | 121 (59.3%) | 83 (40.7%) | 631 | 382 (60.5%) | 249 (39.5%) |

**Note:** Hyper indicates hypermethylated and hypo indicates hypomethylated.

**Supplementary Table S7: Numbers of differentially expressed genes (DEGs) in the tuberization induced process of the treatment (Zeb) and control (CK) of E26.**

| **Name** | **Compare** | **DEGs** | | |
| --- | --- | --- | --- | --- |
|  |  | **Total** | **Up-regulated** | **Down-regulated** |
| **Zeb_7d** | Zeb_7d vs Zeb_0d | 761 | 509 (66.9%) | 252 (33.1%) |
| **Zeb_21d** | Zeb_21d vs Zeb_0d | 1523 | 644 (42.3%) | 879 (57.7%) |
| **CK_7d** | CK_7d vs CK_0d | 611 | 347 (56.8%) | 264 (43.2%) |
| **CK_21d** | CK_21d vs CK_0d | 1395 | 713 (51.1%) | 682 (48.9%) |
| **0d** | Zeb_0d vs CK_0d | 651 | 275 (42.2%) | 376 (57.8%) |
| **7d** | Zeb_7d vs CK_7d | 1284 | 520 (40.5%) | 764 (59.5%) |
| **21d** | Zeb_21d vs CK_21d | 490 | 175 (35.7%) | 315 (64.3%) |
