# Supplementary material for: DNA methylation affects photoperiodic tuberization in potato (Solanum tuberosum L.) by mediating the expression of genes related to the photoperiod and GA pathways
Source: Hortic Res. 2021 Sep 1;8:181. doi: 10.1038/s41438-021-00619-7 (PMC8408180; doi:10.1038/s41438-021-00619-7)
Supplement: Supplementary file 6 — Supplementary Table S10 [file 41438_2021_619_MOESM6_ESM.pdf]

Supplementary Table S10: 68 of the 332 photoperiod response genes differentially expressed in treatment and control at different timepoints of E26 (included 8 reported tuberization regulation genes).

Gene ID	Chromsome	Start position	End position	Annotation	Zeb_7 d vs 0d	Zeb_21 d vs 0d	Zeb_28 d vs 0d	CK_7d vs 0d	CK_21 d vs 0d	CK_28 d vs 0d	Zeb vs CK_0d	Zeb vs CK_7d
PGSC0003DMG400026779	ST4.03ch01	43191763	43197447	PAS/LOV protein A		up	up		up	up		
PGSC0003DMG400011345	ST4.03ch01	58148287	58150947	Flowering promoting factor-like 1	up	up	up					up
PGSC0003DMG400031111	ST4.03ch01	67244732	67252341	SIGB (SIGMA FACTOR B)					up			
PGSC0003DMG400000136	ST4.03ch01	71420222	71427490	PMADS4 protein			down		up			
PGSC0003DMG400000011	ST4.03ch01	71462509	71466702	Gibberellin 20-oxidase 4		down						
PGSC0003DMG400027518	ST4.03ch01	74822199	74827610	GRF zinc finger family protein					down	down		
PGSC0003DMG400013099	ST4.03ch01	79599667	79615846	Conserved gene of unknown function			up					
PGSC0003DMG400001667	ST4.03ch01	86950296	86953032	SAUR family protein		up		up				down
PGSC0003DMG400023897	ST4.03ch02	19962326	19965689	Self-pruning G-box protein (StFDL1)		up	up			up		up
PGSC0003DMG400021095	ST4.03ch02	29584314	29588298	Gibberellin 2-oxidase 1 (StGA2ox1)	up		up					up
PGSC0003DMG400003652	ST4.03ch02	39090264	39093172	Self-pruning G-box protein			down					
PGSC0003DMG400021403	ST4.03ch02	42509285	42515913	GRF domain class transcription factor					down	down		
PGSC0003DMG401010056	ST4.03ch02	45097374	45102578	CONSTANS 3 (CO3 or COL3)	down		down					
PGSC0003DMG400001330	ST4.03ch02	46142998	46148444	Zinc finger protein					down			up
PGSC0003DMG400014823	ST4.03ch03	38777112	38794840	Xyloglucan endotransglucosylase-hydrolase XTH3		down				up	up	
PGSC0003DMG400014322	ST4.03ch03	42490175	42493430	StCEN 1			up			up		up
PGSC0003DMG400003220	ST4.03ch03	46247039	46251632	AtGRF7					down			
PGSC0003DMG400024625	ST4.03ch03	54878384	54887641	MADS box protein (StMADS13)	up	up	up		up	up		
PGSC0003DMG400005745	ST4.03ch03	58415831	58418650	Calcium-binding allergen Ole e 8		down				up		
PGSC0003DMG400005654	ST4.03ch03	58657224	58660750	MFT		up	up					
PGSC0003DMG400000791	ST4.03ch04	58873191	58878822	Photoperiod responsive protein (StPHOR1)						up		
PGSC0003DMG400007951	ST4.03ch04	67891131	67893922	DRE binding protein 1			up	up	up	up		
PGSC0003DMG400030511	ST4.03ch05	3854942	3862025	Repressor of RNA polymerase III transcription MAF1		up				up		
PGSC0003DMG400018408	ST4.03ch05	4537880	4542736	Zinc finger protein (StCDF1)			up					
PGSC0003DMG400034307	ST4.03ch05	14356614	14365165	Histidine kinase 3B						up		
PGSC0003DMG400027206	ST4.03ch05	48612702	48621309	Gibberellin 2-oxidase		up						
PGSC0003DMG400013116	ST4.03ch05	49043450	49046718	Tom	up	up	up					up
PGSC0003DMG400023365	ST4.03ch05	51318128	51321774	Flowering locus T protein (SP6A)	up	up	up		up	up		
PGSC0003DMG400002144	ST4.03ch06	4434580	4438135	NbPCL1 protein		down	down			down		down
PGSC0003DMG400027963	ST4.03ch06	36541515	36545842	GA20 oxidase				up		up		down
PGSC0003DMG400016516	ST4.03ch06	49522127	49526846	Gibberellin 3-oxidase			up					

PGSC0003DMG400004081	ST4.03ch06	51190112	51199207	Agamous-like MADS-box protein AGL8 homolog (StMADS1)	up	up	up		up	up		up
PGSC0003DMG400033046	ST4.03ch06	51597497	51602151	Dof zinc finger protein (CDF2)		up				up		
PGSC0003DMG400033102	ST4.03ch06	51711261	51715053	Phytochrome kinase substrate						up		down
PGSC0003DMG400020053	ST4.03ch06	59029077	59037300	Copalyl diphosphate synthase						down		down
PGSC0003DMG400027475	ST4.03ch07	2274710	2278328	CONSTANS (CO)		up						
PGSC0003DMG400030928	ST4.03ch07	4621738	4627267	EIN3-binding F-box protein 1						down	up	up
PGSC0003DMG400019570	ST4.03ch07	38046167	38049451	ELF4						up		
PGSC0003DMG400029247	ST4.03ch08	1648642	1655633	Patatin group O		up		down	down		down	
PGSC0003DMG400020999	ST4.03ch08	6044342	6051086	Lipoxygenase (StPOTLX-1)					down			up
PGSC0003DMG400007375	ST4.03ch08	40835499	40838631	Chlorophyll a/b-binding protein PS II-Type I		up						down
PGSC0003DMG400022748	ST4.03ch08	53767699	53777641	MADS box transcription factor				up				
PGSC0003DMG400002679	ST4.03ch09	3725996	3730484	GRF domain class transcription factor					down			
PGSC0003DMG400042481	ST4.03ch09	46707109	46709912	Circadian clock coupling factor ZGT		up			up	up		
PGSC0003DMG400017198	ST4.03ch09	56905821	56910285	Proton gradient regulation 5		up			up	up		
PGSC0003DMG402011297	ST4.03ch10	123380	132053	Pseudo-response regulator 9		up	up			up		
PGSC0003DMG400011294	ST4.03ch10	146113	149195	MYB transcription factor MYB114		up	up			up		
PGSC0003DMG400021287	ST4.03ch10	2669819	2673833	Chlorophyll a-b binding protein 8, chloroplastic		up						down
PGSC0003DMG400041963	ST4.03ch10	54070993	54073592	Soul heme-binding family protein			up					
PGSC0003DMG400028259	ST4.03ch10	55686381	55692676	GRF domain class transcription factor		down			down	down		
PGSC0003DMG400016180	ST4.03ch11	3248174	3252562	Flowering locus T (SP5G-like)			down					
PGSC0003DMG400027332	ST4.03ch11	4294001	4296491	Self-pruning interacting protein 1					up			
PGSC0003DMG400019635	ST4.03ch11	40770945	40779427	BEL5 protein (StBEL11)		up	up			up		
PGSC0003DMG400042340	ST4.03ch12	18943287	18946393	CONSTANS		up	up			up		
PGSC0003DMG400028632	ST4.03ch12	49622830	49626982	Flavonoid		down			up		up	
PGSC0003DMG400008545	ST4.03ch12	55308460	55314424	Cryptochrome 1b						up		down
PGSC0003DMG400029365	ST4.03ch12	58152725	58156118	CONSTANS (CO)						up		
PGSC0003DMG400004597	ST4.03ch12	59704934	59707982	Conserved gene of unknown function		up						down
PGSC0003DMG400025129	ST4.03ch02	25587000	25592776	Zinc finger protein								up
PGSC0003DMG400024249	ST4.03ch03	41462794	41466887	Gibberellin 20-oxidase-1 (StGA20ox1)								down
PGSC0003DMG400005698	ST4.03ch03	59434795	59438223	Gibberellin 3-oxidase								down
PGSC0003DMG400003316	ST4.03ch04	67629129	67632587	Tuber-specific and sucrose-responsive element binding factor								up
PGSC0003DMG400023461	ST4.03ch05	51591739	51596108	Chlorophyll a-b binding protein 6A, chloroplastic								down
PGSC0003DMG400021991	ST4.03ch06	773965	779115	GID1-like gibberellin receptor								up
PGSC0003DMG400026500	ST4.03ch06	48019310	48023190	Type I (26 kD) CP29 polypeptide								down
PGSC0003DMG400012329	ST4.03ch08	55112010	55118955	BEL14 protein								down
PGSC0003DMG400003849	ST4.03ch09	51439682	51444078	GID1-like gibberellin receptor								up

PGSC0003DMG400017759	ST4.03ch12	55491383	55494047	MADS-box protein 17	up
